# Identification of Peptidoglycan-Associated Proteins as Vaccine Candidates for Enterococcal Infections

**DOI:** 10.1371/journal.pone.0111880

**Published:** 2014-11-04

**Authors:** Felipe Romero-Saavedra, Diana Laverde, Dominique Wobser, Charlotte Michaux, Aurélie Budin-Verneuil, Benoit Bernay, Abdellah Benachour, Axel Hartke, Johannes Huebner

**Affiliations:** 1 Division of Infectious Diseases, Department of Medicine, University Medical Center Freiburg, Freiburg, Germany; 2 EA4655 U2RM Stress/Virulence, University of Caen Lower-Normandy, Caen, France; 3 Division of Pediatric Infectious Diseases, Dr. von Hauner Children's Hospital, Ludwig-Maximilians-University, Munich, Germany; 4 Proteogen platform SFR ICORE 4206, University of Caen Lower-Normandy, Caen, France; 5 German Center for Infection Research (DZIF), Partnersite Munich, Munich, Germany; The University of Hong Kong, Hong Kong

## Abstract

Infections by opportunistic bacteria have significant contributions to morbidity and mortality of hospitalized patients and also lead to high expenses in healthcare. In this setting, one of the major clinical problems is caused by Gram-positive bacteria such as enterococci and staphylococci. In this study we extract, purify, identify and characterize immunogenic surface-exposed proteins present in the vancomycin resistant enterococci (VRE) strain *Enterococcus faecium* E155 using three different extraction methods: trypsin shaving, biotinylation and elution at high pH. Proteomic profiling was carried out by gel-free and gel-nanoLC-MS/MS analyses. The total proteins found with each method were 390 by the trypsin shaving, 329 by the elution at high pH, and 45 using biotinylation. An exclusively extracytoplasmic localization was predicted in 39 (10%) by trypsin shaving, in 47 (15%) by elution at high pH, and 27 (63%) by biotinylation. Comparison between the three extraction methods by Venn diagram and subcellular localization predictors (CELLO v.2.5 and Gpos-mPLoc) allowed us to identify six proteins that are most likely surface-exposed: the SCP-like extracellular protein, a low affinity penicillin-binding protein 5 (PBP5), a basic membrane lipoprotein, a peptidoglycan-binding protein LysM (LysM), a D-alanyl-D-alanine carboxypeptidase (DdcP) and the peptidyl-prolyl cis-trans isomerase (PpiC). Due to their close relationship with the peptidoglycan, we chose PBP5, LysM, DdcP and PpiC to test their potential as vaccine candidates. These putative surface-exposed proteins were overexpressed in *Escherichia coli* and purified. Rabbit polyclonal antibodies raised against the purified proteins were able to induce specific opsonic antibodies that mediated killing of the homologous strain *E. faecium* E155 as well as clinical strains *E. faecium* E1162, *Enterococcus faecalis* 12030, type 2 and type 5. Passive immunization with rabbit antibodies raised against these proteins reduced significantly the colony counts *of E. faecium E155* in mice, indicating the effectiveness of these surface-related proteins as promising vaccine candidates to target different enterococcal pathogens.

## Introduction

Enterococci have emerged as important nosocomial pathogens due to their multiple antibiotic resistances [1]. *E. faecalis* and *E. faecium* are the third and fourth most commonly isolated nosocomial pathogens worldwide, causing up to 14% and 9,6% of hospital acquired infections in the US and Europe, respectively [2]–[4]. Especially *E. faecium* infections have become a major concern, since resistance to vancomycin and ampicillin have increased to almost 100% in some institutions in the US, and a similar rise of resistances has been observed recently also in Europe [5]–[7]. The ability of this species to survive under a range of adverse environmental conditions, and its dramatic increase in antibiotic resistance worldwide highlights the need for the development of alternative treatment and prevention strategies [8], [9]. To date, many different surface antigens have been identified in *E. faecalis* and *E. faecium*, but only a few of these may be promising vaccine candidates [10].

In Gram-positive bacteria, the cell wall is composed of a peptidoglycan macromolecule that protects bacteria against environmental conditions and serves as anchor for the attachment of capsular polysaccharides, teichoic acids, and proteins that are covalently or non-covalently attached to peptidoglycan [11]. Surface proteins have an important role in the interactions between the bacterial cell and its environment. They are involved in adhesion and invasion of the host cell, sensing the physicochemical conditions of the environment and sending signals to the cytoplasm, in mounting defenses against the host responses and toxicity [12]–[15]. Therefore, surface proteins have become attractive targets for drug development [16]–[18]. Their ability to interact with the host immune system makes them interesting vaccine candidates, since protein based vaccines may overcome some of the challenges encountered by polysaccharide-based vaccines, like serotype-dependent coverage, high production costs, and low immunogenicity [19], [20]. Despite these advantages, only few surface and secreted proteins have been studied in clinically relevant enterococci. Aggregation substance (AS) protein and the collagen adhesin Ace have been examined in *E. faecalis* [21], [22] and enterococcal surface protein Esp, secreted antigen protein SagA and two ABC transporters have been tested for antigenicity in *E. faecium* [23]–[25]. Using appropriate *in vitro* and *in vivo* models to confirm protective efficacy, only SagA, Ace and an ABC transporter were identified as potential vaccine candidates [10], [23].

There are several strategies for the identification of surface proteins. The most widely used techniques are *in silico* analysis of the genome (“reverse vaccinology”), bacterial cell wall fraction analysis by Two-dimensional gel electrophoresis (2-DE) coupled to mass spectrometry, partial enzymatic digestion of cell wall proteins by trypsin (trypsin shaving) and biotinylation [26], [27]. New bioinformatic approaches have been developed and these strategies have significantly improved the prediction of bacterial protein localization. These include the pipelines SLEP (Surface Localization Extracellular Proteins), developed by Giombini *et al* [28], LocateP developed by Zhou *et al* [29], and SurfG+ developed by Barinov *et al* [30]. However, these *in silico* approaches are still not fully reliable and do not provide detailed surface protein localization in the bacterial cell wall [26]. Separations of the membrane and cell wall fractions are analyzed by 2-DE, gel excision of the protein spots and analysis by mass- spectrometry (MS). This strategy has been used in other Gram-positive bacteria [31], [32] and is fairly well established. However, the preparations are usually contaminated with cytoplasmic proteins and often give insufficient information regarding surface exposure, similar to the *in silico* approach [26]. Recently, trypsin shaving has been used in *E. faecalis*, group A *Streptococci*, *Bacillus subtilis* and *Staphylococcus aureus* [26], [27], [33], [34]. This strategy is based on the proteolytic digestion of surface-exposed proteins from intact cells and the analysis of the resulting peptides by liquid chromatography/tandem-mass-spectrometry (LC-MS/MS). The principal advantage of this technique is that it allows a rapid and more selective identification of the surface-exposed proteins. However, it leads to the identification of many cytoplasmic proteins and further verification of location of the identified proteins is necessary [26], [27], [34]. Using biotinylation, intact cells are treated with Sulfo-NHS-SS-Biotin, to which the cell membrane is impermeable. It reacts specifically with the ε-amino-group of lysine residues of surface-exposed proteins. Subsequently, labeled proteins can be separated by affinity chromatography with streptavidin from whole-cell lysates and these can be analyzed by LC-MS/MS or 2-DE [27], [32]. Despite the advantages of being relatively simple to use and facilitating the identification of more predicted surface-exposed proteins compared to cytoplasmic proteins, this method has the major disadvantage that biotin has poor affinity to sortase-attached surface proteins leading to low detection for these important protein antigens [27].

In the current study, we compared the above-described gel-free methods, i.e. trypsin shaving and biotinylation, to correlate the results between them and identify surface protein candidates with greater accuracy. We describe the subsequent overexpression, purification and immunological characterization of surface protein candidates present in hospital-associated vancomycin-resistant *E. faecium* E155 [35] to evaluate their potential role as targets for immunotherapy.

## Materials and Methods

### Bacterial strains and sera

The bacterial strains and sera used for the present study are listed in the Table 1. For the production of polyclonal antibodies against the recombinant proteins, New Zealand white rabbits were immunized with two subcutaneous injections of 10 µg protein given 2 weeks apart; in the third week, three injections of 5 µg were given intravenously. Finally, in the fifth week two injections of 5 µg were given intravenously and the terminal bleeding was collected in the seventh week. Serum from terminal bleedings was heat inactivated at 56°C for 30 min and frozen at −20°C before being used in experiments.

**Table 1:** Bacterial strains and sera used for this study.

Strain or serum	Description*	Reference or source
**Strains**		
*E. faecium* E155	ARE, VRE strain isolated from a patient in the USA (Chicago), CC17	[35]
*E. faecium* E1162	ARE strain isolated from blood in the Netherlands, CC17	[72]
*E. faecalis* 12030	isolated from a patient in the USA (Cleveland)	[44]
*E. faecalis* type 2	isolated from a patient in Japan (Sapporo)	[73]
*E. faecalis* type 5	isolated from a patient in Japan (Kobe)	[73]
*E coli* M15pRep4	M15 harboring pREP4 plasmid	(INVITROGEN)
*E. coli M15*/pQE30LysM	M15 harboring pREP4 and pQE30LysM plasmids	This study
*E. coli M15*/pQE30PpiC	M15 harboring pREP4 and pQE30PpiC plasmids	This study
*E. coli M15*/pQE30DdcP	M15 harboring pREP4 and pQE30DdcP plasmids	This study
*E. coli M15*/pQE30PBP5	M15 harboring pREP4 and pQE30PBP5 plasmids	This study
**Sera**		
NRS	Preimmune sera from rabbit	This study
αSagA	Rabbit serum raised against the recombinant SagA	[23]
αLysM	Rabbit serum raised against the recombinant LysM	This study
αPpiC	Rabbit serum raised against the recombinant PpiC	This study
αDdcP	Rabbit serum raised against the recombinant DdcP	This study
αPBP5	Rabbit serum raised against the recombinant PBP5	This study

*ARE, ampicillin resistant enterococci; CC17, clonal linage complex 17; DdcP, D-alanyl-D-alanine carboxypeptidase; PBP5, low affinity penicillin-binding protein 5; PpiC, peptidyl-prolyl cis-trans isomerase; SagA; major secreted antigen; VRE, vancomycin resistant enterococci.

### Protein extraction by trypsin shaving

Extractions were performed as described by Tjalsma *et al.* [34]. Briefly, two aliquots of 50 mL of bacterial cultures of *E. faecium* E155 grown in brain heart infusion (BHI) were harvested at an OD_600 nm_ = 0.4 by centrifugation (10.000 r.p.m., 2 min) and washed twice with 4 mL Bicam (triethylammonium bicarbonate buffer 100 mM pH 8.0). Then the cells were resuspended in 600 µL of Bicam. The first aliquot was mixed with trypsin (Promega) at a final concentration of 10 µg/mL in Bicam. The second aliquot was resuspended in Bicam without any trypsin. All the samples were incubated for 1 h at 37°C with gentle shaking. After centrifugation (7500 r.p.m., 5 min), the cell pellets were removed and the supernatants were treated with 1 mM dithiothreitol (DTT) for 30 min, followed by 1 mM iodoacetamide (IAA), also for 30 min at room temperature. Finally, fresh trypsin (0.5 µg) was added to all samples and tryptic cleavage was continued for 18 h at 37°C. Proteins identified from the extraction of the second aliquot were digested with trypsin overnight and considered as ‘controls’ to be subtracted from the proteins identified in the cells treated with trypsin after mass spectrometry identification.

### Protein extraction by biotinylation

Surface-exposed proteins were labeled and extracted by exposure of cells to Sulfo-NHS-SS-Biotin using a protocol described by Hempel *et al.* [36] with the following modifications: 100 mL of bacterial cultures of *E. faecium* E155 grown in BHI at OD_600 nm_ = 0.5 harvested at 8000× g for 5 min at 4°C. About 0.2 g of wet cell pellet was resuspended in 5 mL ice-cold phosphate buffered saline (PBS pH 8.0) with 1 mM phenylmethylsulfonyl fluoride (PMSF) and mixed with 0.6 mg of sulfo-NHS-SS-Biotin (Thermo Scientific) previously dissolved in 100 µL of PBS. The mixture was incubated by gentle shaking for 1 h on ice. Unbound biotinylation reagent was removed by centrifugation at 8000× g for 1 min at 4°C and washed three times with ice cold PBS (pH 8.0)/500 mM glycine. Disruption of cells was performed mechanically in a FastPrep cell disrupter (Zymo Research) at 6 m/s^2^ twice for 30 s. The cell debris was recovered from the glass beads with a total of 3 mL of PBS (pH 8.0). The lysate was centrifuged (100.000× g for 1 h at 4°C), the cell debris resuspended in a total of 400 µL of PBS (pH 8.0), supplemented with 5% IAA and homogenized in the cell disrupter at 6 m/s^2^ twice for 30 s with 0.25 mL of glass beads. The proteins were then solubilized by addition of 100 µL of PBS (pH 8.0) with 1 mM PMSF, 4% CHAPS (3-[(3-cholamidopropyl)dimethylammonio]-1-propanesulfonate) and 2% ASB-14 (amidosulfobetaine-14). A second homogenization step was done after detergent addition under the same conditions as mentioned above. Cell debris was removed by centrifugation (14000 r.p.m., 15 min) after 1 h of incubation with the detergent. The biotinylated proteins were isolated and purified by NeutrAvidin (Thermo Scientific) agarose affinity-purification. For a reaction volume of 500 µL protein mixture 150 µL of NeutrAvidin agarose resin was washed twice with PBS (pH 8.0)/1% NP-40 and centrifuged at 1000 r.p.m. for 1 min at 4°C. The resin was mixed with the cell lysate for 1 h by gently shaking on ice. The supernatant was removed and the resin-bound complex washed. Biotinylated proteins were eluted twice by incubation with 1 mL of elution buffer (5% mercaptoethanol in H_2_O) for 1 h with gentle shaking. Supernatant was then recovered after centrifugation at 1000 r.p.m. for 1 min and mixed with 8 mL of cold acetone (−20°C, overnight). The precipitated proteins were harvested by centrifugation (8500 r.p.m., 30 min, 4°C) and washed twice with 1 mL of cold 98% ethanol (4°C). Finally the pellets were dried in a Concentrator 5301 (Eppendorf) for 2 min and dissolved in 15 µL 6M urea/2M thiourea for 2 min at 80°C.

### Elution of cell-wall-associated proteins at high pH

Surface-exposed proteins were extracted by exposure of cells to high pH using a protocol described by Morsczeck *et al.* [37]. A cell pellet from a 50 mL culture of *E. faecium* E155 grown in BHI to OD_600_ = 0.5 was washed with a PBS sucrose solution (100 mM NaCl, 60 mM sucrose, 55 mM sodium phosphate, pH 7.2), and then gently shaken for 1 h at room temperature in 2 mL NaOH glycine sucrose (glycine 50 mM, sucrose 60 mM, pH 12.4). After centrifugation (30 min, 10.000× g), 108 µL 1 M HCl and 100 µL 1 M Tris-HCl (pH 7.0) were added to 1 mL supernatant. Proteins were precipitated at 4°C by addition of 8 mL cold acetone overnight. The protein pellet obtained after centrifugation (10 min, 10.000× g) was resuspended in 200 µL of Tris-HCl (pH 7.5). After an aliquot of 25 µL of the protein solution was run through SDS-PAGE and Coomassie blue staining, each gel line with the protein-containing region was cut in five pieces. After each piece was digested with trypsin as is described in the mass-spectrometry section.

### Mass-spectrometry analyses

Overnight tryptic digestion of the obtained proteins (or peptides) was performed after each extraction method, and subsequently, MS analyses were performed as described elsewhere [38]. In brief, the samples extracted by trypsin shaving, biotinylation and alkaline extraction were treated with 0.5 µg trypsin (Promega) overnight at 37°C. Trypsin-cleaved samples were desalted and concentrated to obtain 1–2 µg of peptides on a tipmicroC18 Omix (Agilent) before nano-liquid chromatography nanoLC-MS/MS analysis. The chromatography step was performed on a nano-LC system (Prominence, Shimadzu). Peptides were concentrated on a Zorbax 5×0.3 mm C18 precolumn (Agilent) and separated onto a Zorbax 150×75 µm C18 column (Agilent). Mobile phases consisted of 0.1% trifluoroacetic acid, 99.9% water (v/v) (A) and 0.1% trifluoroacetic acid, 20% water in 79.9% ACN (v/v/v) (B). The nanoflow rate was set at 300 nL/min, and the gradient profile was as follows: constant 7% B for 5 min, from 7 to 70% B in 183 min, from 70 to 100% B in 5 min, and return to 7% B. The 300 nL/min volume of the peptide solution was mixed with 1.2 µL/min volumes of solutions of 5 mg/mL CHCA matrix prepared in a diluent solution of 50% ACN with 0.1% TFA. Twenty nine second fractions were spotted by an AccuSpot spotter (Shimadzu) on a stainless steel Opti-TOF 384 targets. MS experiments were performed on an AB SCIEX 5800 proteomics analyzer equipped with TOF ion optics and OptiBeam on-axis laser irradiation with a 1000 Hz repetition rate. The resulting fragmentation patterns were used to determine the sequences of the peptides. Database searching was performed using the mascot 2.3.02 program (Matrix Science). A database corresponding to an updated compilation download from the NCBI database was used with *E. faecium* as selected species (including 169 998 entries). The variable modifications allowed were as follows: C-Carbamidomethyl, K-acetylation, methionine oxidation, and dioxidation. Trypsin was selected as the enzyme, with three miss cleavages also allowed. Mass accuracy was set to 200 p.p.m. and 0.6 Da for MS and MS/MS modes, respectively. Finally, to confirm the identity of the recombinant proteins after affinity purification, SDS-PAGE and Coomassie blue staining, the protein-containing regions (bands) were excised, and washed twice with ultrapure water and once with acetonitrile/50 mM ammonium bicarbonate (1∶1, v/v). Samples were stirred for 15 min and vacuum-dried for 30 min. In-gel digestion of the excised protein bands was carried out using 0.5 µg trypsin, incubating overnight at 37°C. MS analysis was performed as described above.

### Determination of protein subcellular localization

The subcellular localization of the proteins was determined using two different *in silico* approaches as follows. The sequence of the identified proteins given by the MS analyses were retrieved from the NCBI data base and analyzed with two Web-server predictors: CELLO v.2.5 (http://cello.life.nctu.edu.tw/) [39] and Gpos-mPLoc (http://www.csbio.sjtu.edu.cn/bioinf/Gpos-multi/) [40]–[42].

### General molecular methods

PCR was performed with Phusion highfidelity DNApolymerase (Finnzymes). The primers used are listed in Table 2. PCR products and plasmids were purified using the NucleoSpin plasmid kit (Macherey-Nagel). Restriction enzymes and T4 DNA ligase were purchased from Promega and used as recommended by the manufacturer. Genomic DNA extraction and other standard techniques were carried out as described by Sambrook *et al.* [43].

**Table 2:** Primers used in this study.

Primer name	5′-3′sequence+	Restriction site
LysM-5-BamHI-2	aggcGGATCCGATGAAGTTTATACAGTAAAATC	BamH I
LysM-3-PstI	aggcCTGCAGGGCTTAGTACCAGCCGTTTG	Pst I
DdcP-5-BamHI-2	aggcGGATCCGAAGATACTTTCAAAGTAAATG	BamH I
DdcP -3-PstI	aggcCTGCAGCAATTAAAACAAGTTACCGAAAA	Pst I
PpiC-5-BamHI-2	aggcGGATCCTGTTCAGGCGATACTAATAAAG	BamH I
PpiC-3-SacI	aggcGAGCTCCTTTTATTTTGATGAATCAGTTG	Sac I
PBP5-5-BamHI	aggcGGATCCATGAAAAGAAGTGACAAGCACG	BamH I
PBP5-3-SacI	aggcGAGCTCAGCAATTTTTTATTGATAATTTTGGS	Sac I

+ Bases in lowercase letters are not complementary to the target sequence.

Underlined bases correspond to restriction sites.

### Construction of *E. coli* strains M15/pQE30LysM, M15/pQE30PpiC, M15/pQE30PBP5 and M15/pQE30DdcP

The proteins were recombinantly expressed to raise antibodies against the different antigens. The respective genes were amplified without the signal peptide using primers listed in Table 2 and genomic DNA from the *E. faecium* E155 as template. The amplified genes were then inserted downstream of the IPTG (Isopropyl β-D-1-thiogalactopyranoside)-inducible promoter into the pQE30 expression vector (QIAexpressionist kit; Qiagen) to obtain an N-terminal His6-tagged recombinant protein. The resulting construct was electroporated into the *E. coli* M15pREP4, creating the different M15/pQE30protein strains (see Table 1). Recombinant proteins were overproduced and purified under denaturing conditions using the Protino Ni-NTA Agarose (Macherey-Nagel) resin, following the manufacture instructions. Finally, the purified recombinant proteins were desalted by diafiltration using the Amicon Ultra-15 Centrifugal Filter Units of 3 KDa (Merck-Millipore).

### Opsonophagocytic assay (OPA) and opsonophagocytic inhibition assay (OPIA)

An *in vitro* opsonophagocytic assay (OPA) was performed as described elsewhere [23], [44]. Briefly, four components were prepared: (a) baby rabbit serum (Cedarlane Laboratories) absorbed with the target bacterial strain as a source of complement, (b) the different rabbit sera (see table 1), (c) polymorphonuclear neutrophils (PMNs) freshly prepared from human blood collected from healthy adult volunteers, and (d) the bacterial strains grown to OD_650 nm_ = 0.4 in tryptic soy Broth (TSB). For the assay, the four components were mixed: 100 µL of PMNs (2.5×10^4^ µL^−1^); 100 µL of the appropriate serum dilution, 100 µL of complement (1∶30 dilution for *E. faecium* strains and 1∶15 for *E. faecalis* strains), and 100 µL of an appropriate dilution of bacteria to yield the desired colony counts (i.e. 1∶1 relation PMNs/bacteria). The mixture was incubated on a rotor rack at 37°C for 90 min, and samples were plated on TSA plates in quadruplicate at time 0 and after 90 min. Percent killing was calculated by comparing the colony counts of a control without PMN's to the colony counts after a 90-minute incubation at 37°C (T90). For inhibition studies, rabbit serum was diluted 1∶50 and incubated for 60 min at 4°C with an equal volume of a diluted sera containing 100 µg of the corresponding protein. Subsequently, the absorbed-serum was used in the OPA as described above. Inhibition assays were performed at serum dilutions yielding 50–60% killing of the inoculum without the addition of the inhibitor. The percentage of inhibition of opsonophagocytic killing was compared to controls without inhibitor.

### Animal model

A mouse bacteremia model was performed to evaluate the passive protection conferred by antibodies raised against the recombinant proteins as described elsewhere [45], [46] with some modifications. In brief, Five female Balb-C mice 6 to 8 weeks-old (Charles River) received intravenously 200 µL of NRS, serum raised against the recombinant proteins or serum raised against recombinant protein SagA as a positive control, 48 and 24 h before the challenge. Bacterial inoculum of *E. faecium* E155 (5.2×10^8^ c.f.u per mouse) was injected *via* the tail vein (i.v.). 24 h after challenge, mice were sacrificed and colony counts in kidneys were determined by homogenizing and plating of serial dilutions.

### Statistical Analysis

The software program GraphPad PRISM version 5.00 was used for the statistical analyses. The percentage of organisms killed using immune sera in the OPA was expressed as geometrical mean ± the standard error of the means. Statistical significance for the OPA and OPIA was determined by ANOVA and Dunnett's Multiple Comparison Test. A p value of <0.05 was considered significant. Significance of the bacterial counts in the animal experiment was determined by analysis of variance for multi-group comparisons using log-transformed data, and Dunnett post hoc test. A p value of <0.05 was considered significant.

### Ethics Statement

All animal experiments were performed in compliance with the German animal protection law (TierSchG). The mice were housed and handled in accordance with good animal practice as defined by FELASA and the national animal welfare body GV-SOLAS. The animal welfare committees of the University of Freiburg (Regierungspraesidium Freiburg Az 35/9185.81/G-12/070) approved all animal experiments.

## Results

### Identification of surface related proteins in *E. faecium* E155

**Figure 1:**
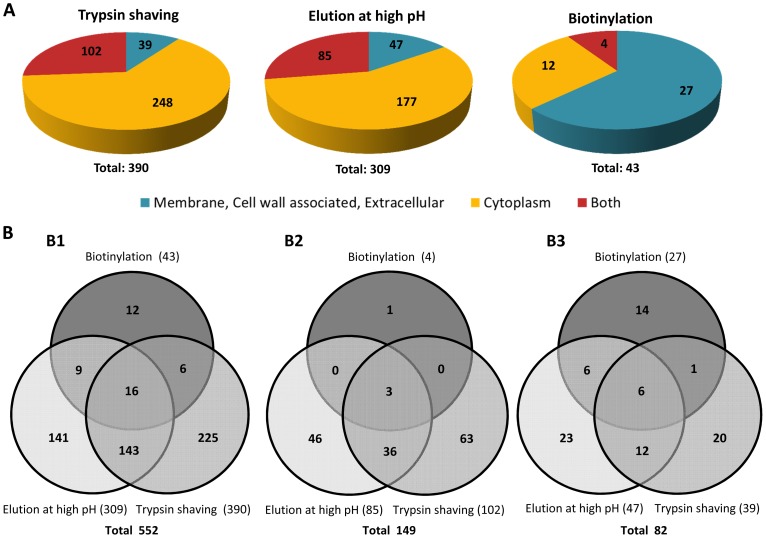
Distribution of *E. faecium* E155 proteins identified by the different extraction methods. (A) Rate of *E. faecium* E155 proteins identified with one or more unique peptides in at least two biological replicates by trypsin shaving, elution at high pH and biotinylation and their corresponding subcellular localization predicted by Cellov.2.5 (http://cello.life.nctu.edu.tw) and Gpos-mPLoc (http://www.csbio.sjtu.edu.cn/bioinf/Gpos-multi). (B) Venn-diagram of all the proteins identified by the different extraction methods. (B1) Correlation between the proteins extracted by the different extraction methods. (B2) Correlation between the proteins predicted to have both cytoplasmic and extracytoplasmic location by CELLO v.2.5 and Gpos-mPLoc. (B3) Correlation between the Proteins predicted to have exclusively an extracytoplasmic location by CELLO v.2.5 and Gpos-mPLoc.

For a more accurate identification of surface proteins in the *E. faecium E155* strain, three different approaches were used: trypsin shaving, biotinylation and high pH elution. The number of proteins identified by MS analysis containing at least one unique peptide in at least two sample replicates (see supplementary tables S1 to S3) for the different methods were 390 for trypsin shaving, 309 for elution at high pH, and 45 for biotinylation. We analyzed the sequence of each protein through two Web-server predictors (CELLO v.2.5 and Gpos-mPLoc) to evaluate their sub-cellular localization. For each of the three methods, proteins were then classified in three main groups: a) Inside: If a protein was predicted to have an exclusively cytoplasmic location by both algorithms we considered it to be inside of the cell. b) Both: If one of the algorithms predicted that the subcellular localization of a protein is intracellular (cytoplasmic) and the other predicted that is outside of the cytoplasm (i.e. membrane, cell wall associated and/or extracellular) OR if the algorithms predicts two locations inside and outside of the cytoplasm (Cytoplasm-membrane or cytoplasm-extracellular) at the same time, the protein was considered to be both inside and outside of the cytoplasm. c) Surface-associated: If a protein was predicted to have an exclusively extracytoplasmic location (i.e. membrane, cell wall associated and/or extracellular) by both algorithms we considered these proteins as surface-associated. Among all the proteins identified, 39 (10%), 47 (15%) and 27 (63%) polypeptides were predicted to be extracytoplasmic by trypsin shaving, elution at high pH, and biotinylation, respectively (see figure 1A). On the other hand, we observed that 102 (26%) proteins obtained by trypsin shaving, 85 (27%) by elution at high pH and 4 (9%) by biotinylation were predicted to have both cytoplasmic and extracytoplasmic location. The data were then compared using Venn-diagrams (figure 1B1) to identify proteins classified as surface-associated by more than one method. A total of 552 proteins with at least one unique peptide were uncovered and among them 16 proteins were identified by all three methods; 158 proteins appeared at least in two of the three different extraction procedures. We compared the proteins predicted to have cytoplasmic and extracytoplasmic localizations (see figure 1B2). Three of them were part of those polypeptides identified by all three methods, while 36 appeared at least in two (see supplementary table S4). Finally, we compared the proteins that were predicted to have an extracytoplasmic location (figure 1B3), showing that six of them appeared in all the extraction methods and 23 were identified by at least two of the three methods. Extracytoplasmic proteins identified by more than one method and their subcellular localization are summarized in table 3. Considering these results, we assumed that the six extracytoplasmic proteins identified by all three extraction methods were the most promising candidates to study immunogenicity and protective efficacy. Among the six proteins, we finally decided to focus on four that interact with peptidoglycan (PG) and are more likely to be surface-exposed: (a) the 21.6 kDa peptidoglycan-binding protein LysM (LysM) that has been reported to be non-covalently attached to PG [47]; (b) the 73.7 kDa low-affinity penicillin-binding protein 5 (PBP5) that is involved in polymerization of PG [48]–[50], (c) the 47.7 kDa D-alanyl-D-alanine carboxypeptidase (DdcP) - a low molecular weight penicillin binding protein (LMW-PBP) cross-linking PG chains to form rigid cell walls [49], [51] and (d) the 37.3 kDa PpiC-type peptidyl-prolyl cis-trans isomerase (PpiC) also involved in PG cross-linking [52].

**Table 3:** Summary of the proteins identified by at least two of the three extraction methods and predicted to have an extracytoplasmic location.

		Subcellular localization^b^		Extraction method		
Protein name	Gene Locus^a^	CELLO v.2.5	Gpos-mPLoc	Biot*	Tryp§	HpH$
Peptidoglycan-binding protein LysM	EFF34034	Ext-CW	Ext-Cw	+	+	+
Low affinity penicillin-binding protein 5	EFF35784	Ext	Ext-CW	+	+	+
D-alanyl-D-alanine carboxypeptidase	EFF35669	Mem	Mem	+	+	+
PpiC-type peptidyl-prolyl cis-trans isomerase	EFF34785	Ext-Mem	Ext-Mem	+	+	+
SCP-like extracellular protein	EFF35540	Ext	Ext	+	+	+
Basic Membrane lipoprotein	EFF34523	Ext	Mem	+	+	+
Glycosyl transferase	EEV52587	Ext	Ext	+	+	−
DNA-entry nuclease	EEI59681	Ext	Ext	+	−	+
Extracellular solute-binding protein, family 5	EAN09846	Ext	Ext	+	−	+
NLPA lipoprotein	EAN09985	Mem	Mem	+	−	+
Peptidase M41, FtsH	EAN10268	Mem	Mem	+	−	+
Extracellular solute-binding protein, family 3	EAN08986	Mem	Mem	+	−	+
Periplasmic solute binding protein	EAN10630	Mem	Mem	+	−	+
Cell envelope-related transcriptional attenuator	EAN08970	Ext	Ext	−	+	+
Peptidase S1, chymotrypsin	EAN09870	Ext-Mem	Ext	−	+	+
Beta-ketoacyl-acyl carrier protein synthase III (FabH)	EAN10058	Mem	Ext	−	+	+
50S ribosomal protein L2	EEI61156	Mem	Ext	−	+	+
Peptidylprolyl isomerase	EEI59596	Mem	Ext	−	+	+
Metallo-beta-lactamase superfamily protein	EEV42569	Mem	Ext	−	+	+
Penicillin-binding protein	EEV43240	Ext	Mem	−	+	+
ABC superfamily ATP binding cassette transporter	EEI61138	Mem	Mem	−	+	+
Family 2 glycosyltransferase	EEI61366	Mem	Mem	−	+	+
VANA ligase	CAA40215	Mem	Mem	−	+	+
PilT protein, N-terminal	EAN10184	Mem	Mem	−	+	+
Helicase, C-terminal: DEAD/DEAH box helicase	EAN08953	Mem	Mem	−	+	+

a Gene locus given by blast in the NCBI (http://www.ncbi.nlm.nih.gov/);

b subcellular localization predicted by Cellov.2.5 (http://cello.life.nctu.edu.tw) and Gpos-mPLoc (http://www.csbio.sjtu.edu.cn/bioinf/Gpos-multi).

CW, cell wall. Ext, extracellular. Mem, membrane.

*Biot; Biotinylation.

§ Tryp; Trypsin shaving.

$ HpH; Elution at high pH.

### The target proteins induce opsonic and cross-reactive antibodies

**Figure 2:**
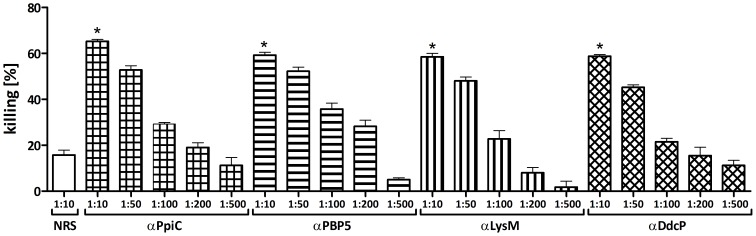
Opsonophagocytic assay against the homologous strain *E. faecium* E155. Opsonophagocytic assay used to test the ability to mediate opsonic killing in the strain *E. faecium* E155 by antibodies raised against the recombinant proteins at different dilutions. αPpiC (square grid), αPBP5 (horizontal stripes), αLysM (vertical stripes) and αDdcP (rhombic grid), compare with the activity of the preimune rabbit serum (NRS, white bar). Bars represent the mean of data and the error bars represent the standard error of the mean. Statistical significance was determined by ANOVA and Dunnett's Multiple Comparison Test. Comparing killing rates of similar dilutions (i.e. 1∶10) with the NRS, all comparisons were significant at p<0.001 (indicated by asterisk).

**Figure 3:**
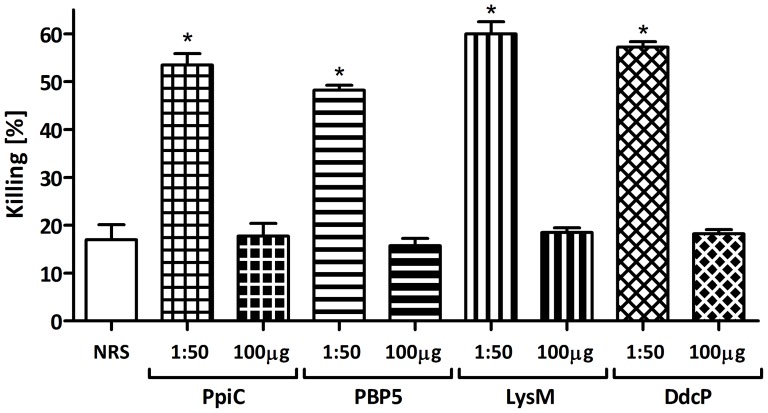
Specificity of the antibodies raised against the recombinant proteins. The sera were used at final dilution of 1∶50, PpiC (square grid), PBP5 (horizontal stripes), LysM (vertical stripes) and DdcP (rhombic grid) and the strain tested was *E. faecium* E155. Purified recombinant proteins were used as inhibitors at concentration of 100 µg/mL, and were preincubated with the corresponding sera dilution for 1 h at 4°C prior to OPA. Opsonic killing of the target strain with non-absorbed antibodies was used to assess the reduction of opsonic killing produced by each inhibitor, using preimune rabbit serum (NRS, white bar) as a Control. Bars represent the mean of data and the error bars represent the standard error of the mean. Statistical significance was determined by ANOVA and Dunnett's Multiple Comparison Test. Comparing killing rates of similar dilutions (i.e. 1∶50) with the NRS, all comparisons were significant at p<0.001 (indicated by asterisk).

The genes encoding the four candidate proteins were amplified without their signal peptides, cloned into the pQE30 expression vector and transformed into *E. coli*. The recombinant proteins were then purified under denaturing conditions. The purity of the proteins was assessed by SDS-PAGE and their identity was confirmed by LC-MS/MS (data not shown). New Zeeland white rabbits were immunized with purified proteins and exsanguinated two weeks after the last injection. The obtained polyclonal antibodies raised against the different proteins were tested in an OPA against the corresponding strain *E. faecium* E155 showing that all the proteins were able to induce opsonic antibodies. Different concentrations were tested to titer out the opsonic activity of the sera. Maximum opsonic activity of the antibodies was between 58–65% of killing with a 1∶10 serum dilution, and a reduction of killing was observed in a dose dependent fashion using increasingly higher dilutions of sera (see figure 2). To verify the specificity of the killing against the respective recombinant protein, opsonophagocytic inhibition assays (OPIA) were carried out by pre-incubating the sera with 100 µg/mL of the corresponding recombinant protein. These sera were then tested in an OPA using *E. faecium* strain E155 which showed that opsonic killing is inhibited by more than 85% in all cases (see figure 3).

### Specific and opsonic antibodies against the recombinant proteins are cross-reactive with different *E. faecium* and *E. faecalis* isolates

**Figure 4:**
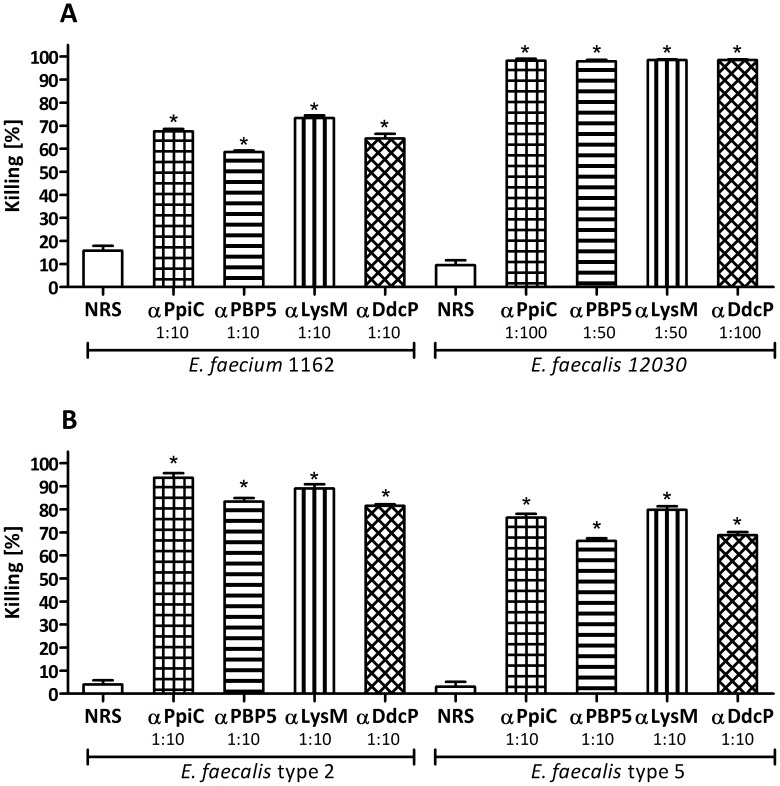
Cross-reactivity of the sera against different enterococcal strains. Opsonophagocytic assay used to test the ability to mediate opsonic killing of different enterococcal strains by antibodies raised against the recombinant proteins. A) Opsonophagocytic killing of strains *E. faecium* E1162 and *E. faecalis* 12030 by antibodies raised against the recombinant proteins at dilutions between 1∶10 and 1∶100. αPpiC (square grid), αPBP5 (horizontal stripes), αLysM (vertical stripes) and αDdcP (rhombic grid), compared with the activity of the preimune rabbit serum (NRS, white bar). B) Opsonophagocytic killing in *E. faecalis* type 2 and *E. faecalis* type 5 by antibodies raised against the recombinant proteins at dilution 1∶10. αPpiC (square grid), αPBP5 (horizontal stripes), αLysM (vertical stripes) and αDdcP (rhombic grid), compared with the activity of the preimune rabbit serum (NRS, white bar). Bars represent the mean of data and the error bars represent the standard error of the mean. Statistical significance was determined by ANOVA and Dunnett's Multiple Comparison Test. Comparing killing rates of similar dilutions (i.e. 1∶10, 1∶50 or 1∶100) with the NRS, all comparisons were significant at p<0.001 (indicated by asterisk).

To determine if the antibodies directed against the recombinant proteins were able to opsonize different strains, serum dilutions between 1∶10 and 1∶100 were tested in OPAs against *E. faecium* E1162 and *E. faecalis* 12030, type 2 and type 5 [46], [53]. The four sera were able to opsonize all strains exhibiting killing above 60% (see Figure 4A and 4B). Passive immunization with antibodies directed against the different proteins promotes clearance of bacteria in mice

**Figure 5:**
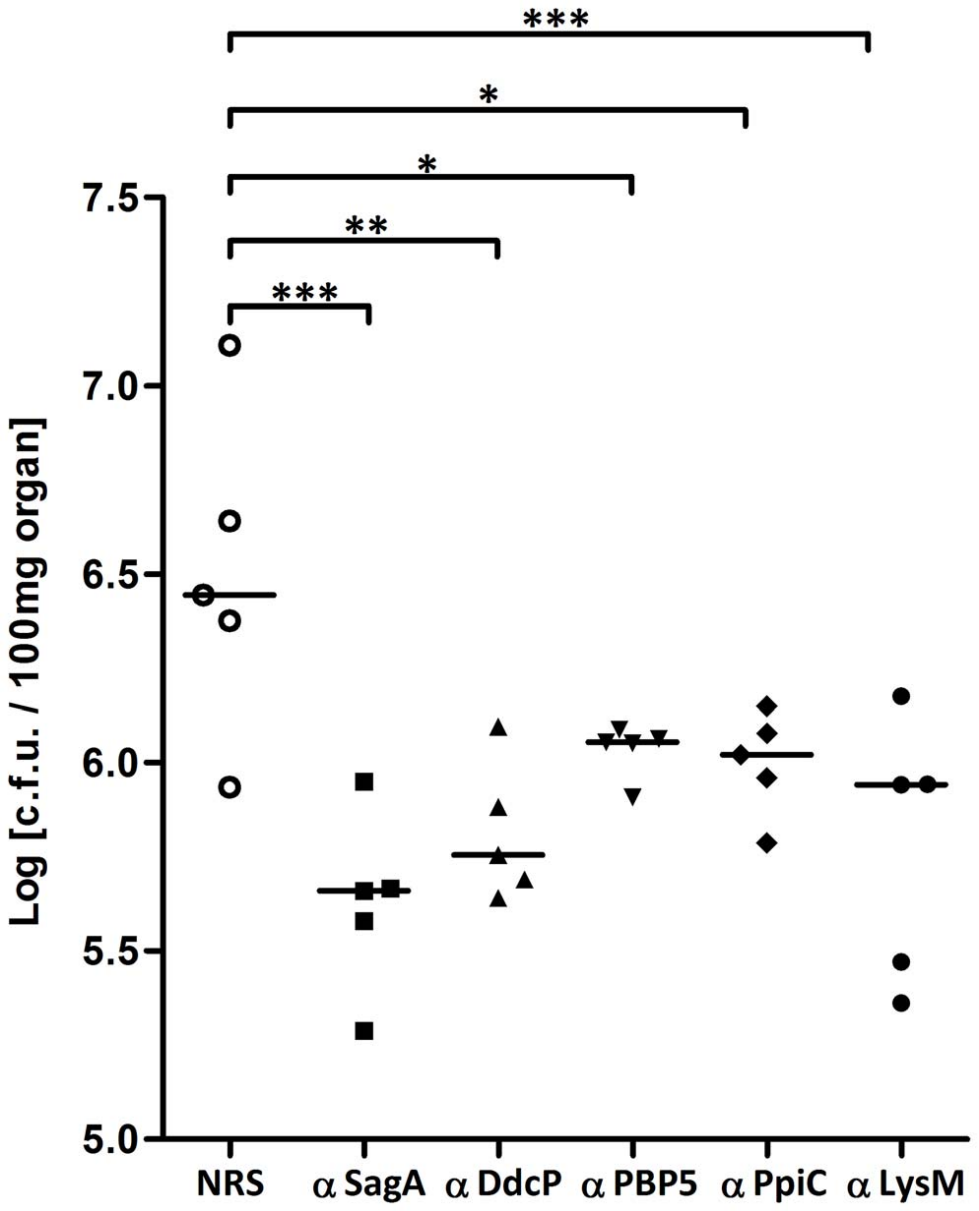
Protection against bacteremia in mice. Passive Immunization with the antibodies raised against the recombinant proteins promotes clearance of *E. faecium* E155 in mouse kidney in comparison with the normal rabbit serum. 24 h after the bacterial challenge mice were killed and kidneys were removed to assess viable counts. Each point represents the bacterial counts from a single mouse. Bars indicate the median CFU/100 mg of kidney for the group. P value was <0.05 (*P≤0.05, **P≤0.01, ***P≤0.001) for comparison between the animals immunized with the antibodies raised against the recombinant proteins and control animals immunized with preimune rabbit serum (NRS) determined by analysis of variance for multi-group comparisons using on log-transformed data, and Dunnett post hoc test.

To determine if antibodies directed against the recombinant proteins are protective in a mouse bacteremia model, mice were passively immunized twice within 48 h before bacterial infection. Sera raised against the four recombinant proteins significantly reduced *E. faecium* E155 colony counts in the kidneys. These results are comparable to the protection achieved by antibodies raised against the previously reported antigen SagA [23]. Immunization with the sera raised against PpiC and PBP5 proteins resulted in higher viable counts (i.e. less protection) (P value≤0.05) compared to serum raised against DdcP (P value≤0.01) and LysM (P value≤0.001) (see Figure 5).

## Discussion

It has been reported by *in silico* analysis that between 30 to 40% of the bacterial proteome corresponds to surface-associated proteins. However, few of these proteins have been physicochemically and immunologically characterized [26], although surface-exposed and secreted proteins have been shown to be promising vaccine candidates in some pathogenic bacterial species [23], [54], [55]. Surface-exposed proteins can be identified more or less successfully by *in silico* approaches or with different extraction methods, such as trypsin shaving and biotinylation [15], [26], [27], [36], [54]. Maione *et al.* used multiple genome screening approaches in group B *Streptococcus*, identifying 589 predicted surface-exposed proteins. They overexpressed and tested 312 of these candidates, but only four were found to be potential vaccine candidates [54]. In group A *Streptococcus*, trypsin shaving has been shown to be a useful technique to extract surface-exposed proteins. Rodríguez-Ortega and coworkers were able to identify 72 proteins and demonstrate that 95% corresponded to extracytoplasmic proteins and around 86% of them were effectively surface-exposed [26]. However, in our study trypsin shaving was not the most efficient method. Indeed, only 36% of the identified proteins were predicted to have an extracytoplasmic location. This is in agreement with the findings of Hempel *et al.* in *Staphylococcus aureus*, showing that by trypsin shaving only 41% of the extracted proteins corresponded to surface-exposed proteins [27]. It is important to point out that some of these proteins that we classified as cytoplasmic proteins by different web-server predictors (e.g. enolase, Inosine-5′-monophosphate dehydrogenase, glyceraldehyde-3-phosphate dehydrogenase, pyruvate dehydrogenase, triosephosphate isomerase, elongation factor Tu, and GroEL) have been described as “moonlight proteins” since they perform more than one function on the cell [56], [57] and have been identified on the cell surface of some gram-positive bacterial pathogens [33], [58]–[67]. Although the proteins mentioned above are predicted to be cytoplasmic we cannot be ruled out their possible surface location and should be consider as good candidates for further immunological studies. The combined results may indicate that the efficiency of trypsin shaving may be species-dependent. In the case of *E. faecium*, we demonstrated that biotinylation was the most accurate and specific procedure for the identification of extracytoplasmic proteins with a yield of 72%. In the present study the killing of bacteria in the opsonophagocytic assay indicates that the respective target is accessible for antibodies and complement. However, additional methods (such as immuno-electron microscopy or confocal microscopy) are necessary to confirm surface exposure.

Only one protein has been identified so far as a potential vaccine target in *E. faecium*. The major secreted protein SagA induced opsonic and protective antibodies in rabbit, that were able to mediate *in vitro* opsonophagocytic killing against the homologous strain and to reduce colony counts in mice [23]. Additionally, an antibody isolated from a phage display antibody library, directed against an epitope present in an ABC transporter protein has been described to promote clearance of *E. faecium* in mice, suggesting its possible use in immunotherapy [24]. The peptidoglycan-associated proteins tested as vaccine candidates in the present study have been implicated in antibiotic resistance and virulence. Penicillin-binding proteins, such as PBP5 and Ddcp, have been reported to play a key role in intrinsic resistance to β-lactams, being the major contributors to ampicillin resistance in *E. faecium* [51]. In *E. faecalis*, the homologue to protein PpiC has been characterized as a potential virulence factor that confers resistance to high NaCl concentrations and ampicillin, because this protein is involved in the folding and trafficking of extracellular proteins, especially PBPs [52], [68]. LysM, which is non-covalently attached to peptidoglycan, has been reported to be involved in early stages of erythromycin resistance in *E. faecalis*, but its precise function has not been elucidated yet [69]. All these proteins are clearly potential targets for drug development and we show here that they could also be interesting for vaccine development.

We were able to demonstrate that all four proteins induced opsonic antibodies in rabbits, which mediate effectively *in vitro* opsonophagocytic killing (higher than 50%) not only of the homologous strain but also of other enterococcal strains, i.e. *E. faecium* E1162 (belonging to clonal complex 17 [70]), *E. faecalis* 12030, *E. faecalis* type 2 and *E. faecalis* type 5 [53]. The broad cross-reactivity of the sera indicates that these protein antigens may effectively supplement serotype-dependent coverage of polysaccharide-based vaccines. The lower opsonophagocytic killing observed against the homologous strain compared with *E. faecium* E1162 and *E. faecalis* 12030, may be attributed to the surface accessibility of the protein antigens that vary from strain to strain, even if the antigen's encoding genes are conserved [54]. Such variability may be due to differences in gene expression, antigen masking by other cell wall components, protein degradation, or other factors [26], [54]. The presence of a putative antiphagocytic polysaccharide capsule in *E. faecium* E155, similar to the one found in *E. faecalis* serotypes C and D, may mask protein antigens, making them less available for binding or less accessible for complement components or phagocytes [46]. A similar effect was observed for *E. faecalis* Type 2 and Type 5 since these strains were killed to a lesser extent than *E. faecalis* 12030. Compared to this strain, 10 times higher serum concentrations were necessary to observe similar killing of these strains, strengthening the suggestion that masking may be the reason for the reduced opsonophagocytic killing observed in *E. faecium* E155. Specificity of the sera raised against the different proteins was demonstrated by the reduction of the opsonophagocytic killing elicited by serum absorbed with the corresponding recombinant protein. The OPA is known to correlate well with *in vivo* immune response and is considered a surrogate for the human protective immune response [10]. This assay is an indicator for the bacteria's ability to survive in the human blood and to cause infections [71]. We observed a good correlation between the ability of the antibodies raised against the different recombinant proteins to mediate opsonic killing *in vitro* and promote a statistically significant reduction of bacteria in mice after i.v. challenge.

In summary, we compared three existing extraction methods for bacterial surface proteins that are likely to interact with the host immune system. These proteins can be targets for drugs aimed at preventing bacterial infections and diseases, or could be used as components for conjugate vaccines. We demonstrate that the four peptidoglycan associated proteins identified by this approach, i.e. LysM, DdcP, PpiC and PBP5 elicit specific, opsonic and protective antibodies, with a broad cross-reactivity and serotype-independent coverage among *E. faecalis* and *E. faecium*. These antigens are interesting targets to be used as single component or as carrier proteins together with polysaccharide antigens in vaccine development against enterococcal infections.

## Supporting Information

Table S1 **Summary of all the proteins identified by trypsin shaving.**
(DOCX)

Table S2 **Summary of all the proteins identified by elution at high pH.**
(DOCX)

Table S3 **Summary of all the proteins identified by biotinylation.**
(DOCX)

Table S4 **Summary of all the proteins identified by at least two of the three extraction methods and predicted to have both cytoplasmic and extracytoplasmic location.**
(DOCX)
