# Supplementary material for: Identification of Peptidoglycan-Associated Proteins as Vaccine Candidates for Enterococcal Infections
Source: PLoS One. 2014 Nov 4;9(11):e111880. doi: 10.1371/journal.pone.0111880 (PMC4219796; doi:10.1371/journal.pone.0111880)
Supplement: Table S1 — Summary of all the proteins identified by trypsin shaving. (DOCX) [file pone.0111880.s001.docx]

**Table S1.** Summary of all the proteins identified by trypsin shaving.

| Protein name | Gene Locus^a^ | No of replicates | Peptides identify by MS analyses | Subcellular localization ^b^ | |
| --- | --- | --- | --- | --- | --- |
|  |  |  |  | CELLO v.2.5 | Gpos-mPLoc |
|  |  |  |  |  |  |
| Elongation factor Tu | EEI61151 | 3 | EHILLSR  TTVTGVEMFR  ALEGDASYEEK  TTVTGVEMFRK  GQVLAKPGTITPHTK  FSAEVYVLTKEEGGR  LLDYAEAGDNIGALLR  GITISTAHVEYETDTR  SKPHVNIGTIGHVDHGK  KLLDYAEAGDNIGALLR  ILELMAAVDEYIPTPER  VDMVDDEELLELVEMEVR  DNDKPFMMPVEDVFSITGR  NGGQAMAYDQIDGAPEERER  DLLTEYEFPGDDVPVVAGSALK  KNGGQAMAYDQIDGAPEERER  NMITGAAQMDGAILVVSAADGPMPQTR | Cyt | Cyt |
| Translation elongation factor G | EEI61150 | 3 | LSEEDPTFR  VEANVGAPQVSYR  IGADFLYSVSTLHDR  GTFTMTFDHYEDVPK  EFKVEANVGAPQVSYR  REFKVEANVGAPQVSYR  YLEGEEITEAELKEGIR  LYDGSYHDVDSNETAFR  INIIDTPGHVDFTVEVER  TDEEVERPADDSAPFSALAFK  IGETHEGASQMDWMEQEQER  VLDGAVTVLDAQSGVEPQTETVWR  AEMYTNDLGTEIEETEIPEEYR  AEMYTNDLGTEIEETEIPEEYRELAEEWR  AEMYTNDLGTEIEETEIPEEYRELAEEWREK | Cyt | Cyt |
| Phosphoribosylformylglycinamidine synthase II | EAN09784 | 3 | FGELDNPR  NFDLSIEKR  IGGQIAVAEAAR  AGSGLILTLDEVPQR  IYAEWGLTDEEYR  TRFEEMMGDAAVLAGK  ETGMTPYEMMLSESQER  DGIHGATFASEEFNQEEEQQR  AESHNHPSAVEPYEGAATGVGGIIR  LYHQGEEVANLPVDALAEDAPVYHK | Cyt | Cyt |
| Translation elongation factor Tu:Small GTP-binding protein domain | EAN08775 | 3 | EHILLSR  AVVTGVEMFR  TLDYGEAGDNVGVLLR  HYAHIDAPGHADYVK  SKPHVNIGTIGHVDHGK  KTLDYGEAGDNVGVLLR  GITINTAHVEYETEKR  ELLSEYGFPGDDTPVIK  GLANPQDYASIDAAPEER  KGLANPQDYASIDAAPEER  DTDKPLLLPVEDVFSITGR | Cyt | Cyt |
| Cell division protein FtsZ | EAN10730 | 3 | VIGVGGGGGNAVNR  EFDTFNREETK  GVEFITANTDVQALK  SHGDDELNTPPFFR  VTVIATGIDESKKER  LKENVDTLLIISNNR  ELGALTVGVVTRPFTFEGPK  TVMENQGTALMGIGVASGEDR  TVLDMDQAKPTSSEEENSFGDWDIR  TVLDMDQAKPTSSEEENSFGDWDIRR | Cyt | Cyt |
| VANA ligase | CAA40215 | 3 | VDMFLQDNGR  MMAAAGIALPELIDR  VNSADELDYAIESAR  KVNSADELDYAIESAR  IVLNEVNTLPGFTSYSR  KNHEYEINHVDVAFSALHGK  IHQEVEPEKGSENAVITVPADLSAEER | Mem | Mem |
| 30S ribosomal protein S4 | EEI61012 | 3 | QLVNHGHILVDGK  GKQSEYGMQLTEK  VDIPSYHVEVGQVISVR  EAVEATVGRPAFVSFDAEK  RVDIPSYHVEVGQVISVR  EAVEATVGRPAFVSFDAEKLEGSLTR  LPERDELYPEIDEALVVEYYNQSL | Cyt | Cyt |
| Peptidase M41, FtsH | EAN10268 | 3 | SMVTEYGMSDR  NAPAIIFIDEIDAVGR  AYSEQVAFEIDQEVR  LGPVQYEGNHQVFVGR  KNAPAIIFIDEIDAVGR  AYSEQVAFEIDQEVRK  VVAQQTPGFAGADLENVLNEAALVAAR  FSDVAGAEEEKQELVEVVEFLKDPR | Mem | Mem |
| UTP--glucose-1-phosphate uridylyltransferase, bacterial and archaeal type | EEI59439 | 3 | GLGHAVLQAR  LVEETTDVNLHFIR  TSIEYGLTHPEVGAPLR  THASTIAVMQVPHDETSK  YGIINPGEVLEDGLYNVK  NFVEKPDPSKAPSDLAIIGR  AKRPIEDHFDSNIELETNLSEK  TSIEYGLTHPEVGAPLREYILSLGK | Cyt | Cyt |
| Ribosomal protein S3 | EEV48626 | 3 | LADAAVSTVEIER  EYAEFLHEDLR  SEGYSEGTVPLHTLR  ADIDYAWEEADTTYGK  WYAEKEYAEFLHEDLR | Cyt | Cyt |
| ATP synthase F1, beta subunit | EAN09186 | 3 | FTQAGSEVSALLGR  VALTGLTIAEYFR  TAMVFGQMNEPPGAR  EILEGKYDDLPEEAFR  MPSAVGYQPTLATEMGQLQER  VFNVLGDTIDLETPFPEDAER | Cyt | Cyt |
| SAICAR synthetase | EAN09787 | 3 | VQIIPLEVVIR  GELNNQITSAIFR  TNEHMDKDVYRR  DLGDLIPVYQEVYNR  RDLGDLIPVYQEVYNR  KDQISGKGELNNQITSAIFR  KIYLTSDENLIWIEYLDQATALNGAR | Cyt | Cyt |
| Helicase, C-terminal:DEAD/DEAH box helicase, N-terminal | EAN08953 | 3 | VQAVYGGADIGR  ELAIQTQEELYR  EMTADLIDQYYVR  LFDVQTPELTIVFGR  AKEMTADLIDQYYVR  IDPDRHELQGLVIAPTR  GLDISGVTHVYNYDIPQDPESYVHR | Cyt-Mem | Mem |
| Septation ring formation regulator, EzrA | EEW64688 | 3 | QHEIPYSEVR  RIEDFYFNHPDLV  EYHYQEALDEIGTALER  VREDEHEYGPALFELQK  THDLVDAAALTEQMLQYANR  RKEELFDLPVIEEVDDVKK  VLEDIEKQQVEIDDSLHELR  VIEKEFTQFVTLNTSGDPVEAR  GFQTEVEEMERQNEQMIPQIR | Cyt | Mem |
| Ribosomal protein S5, bacterial and organelle form | EAN09419 | 3 | SLGSNTPINVVR  HLELEDRVVAINR  ILMKPAVEGSGVAAGGPVR  RAEEVAELRGKSVEELIG  VYIDPKHLELEDRVVAINR  NLVEVPMVGSTIPHEVIGAFSGGR  KNLVEVPMVGSTIPHEVIGAFSGGR  AIEDAKKNLVEVPMVGSTIPHEVIGAFSGGR | Cyt | Cyt |
| SecA protein | EAN08595 | 3 | VLIVDQFTGR  FHAVVQDIKDR  WTDHIDAMDQLR  GETLDQLLPEAFAVVR  MFEDMVGAIEYEVTR  YQKGETLDQLLPEAFAVVR  QGDPGMSQFYLSLEDDLMKR | Cyt | Cyt |
| Aicarft/imp cyclohydrolase | EEV44106 | 3 | QYGHTSLSFR  YDLAMVFTDVR  TLSIEEVTGFPEMMDGR  NHEFVTSVVDPSDYEEVLSELR  ETIMKPDVEIAEAIENIDIGGPSMLR | Cyt | Cyt |
| Asparaginyl-tRNA synthetase, class IIb | EAN09362 | 3 | TVTWIAGIDHVR  ATDYDFLLEQIR  YGSVPHSGFGLGLER  NLHQETSVLVTGEIR  NHGLDEKEYSWYLDLR | Cyt | Cyt |
| Glucosamine-fructose-6-phosphate aminotransferase | EEV41461 | 3 | VIIQNFAGDIVER  YVTEDNRVSLDQK  INIDTTGNIGIGHTR  NVTPGLIDGLEKLEYR  WATHGQPSEENAHPHTSQSGR  HGTIALIEEGTPVIGIISEEVTGAHTR | Cyt | Cyt |
| Ribosomal protein S2 | EEV41522 | 3 | WNPKMK  FLGGIADMPR  IPDVMYIVDPR  LEKFLGGIADMPR  WLGGTLTNWDTIQKR  QAQEAIKEEATRAGQYFVNHR | Cyt | Cyt |
| Small GTP-binding protein domain:GTP-binding protein TypA | EAN09390 | 2 | ATTYSIMSIEER  QSDTLDAHTQLQER  EGYELQVSRPEVIEK  VNIMDTPGHADFGGEVER  GTVFVEPGTEVYEGMIIGENSR | Cyt | Cyt |
| Catabolite control protein A | EEI59881 | 3 | QTITIYDVAR  LTSITQPLYDLGAVSMR  QVDGVIFMGHHITDEIR  VPEEFEIITSNNSLLTEVARPR  GVKVPEEFEIITSNNSLLTEVARPR | Cyt | Cyt |
| RecA recombinase | CAF21832 | 3 | SGAWYSYKEDR  VGVMFGNPEITPGGR  AEIDGEMGDSHVGLQAR  IYMANHPEMMAEVSALVR  IDQQISTIPSGSLALDVALGVGGYPR | Cyt | Cyt |
| S-adenosylmethionine synthetase | EAN08618 | 3 | IIVDTYGGYAR  KIIVDTYGGYAR  FVIGGPQGDAGLTGR  FVIGGPQGDAGLTGRK  EVIPAELLDDQTKYYINPTGR  VVKEVIPAELLDDQTKYYINPTGR | Cyt | Cyt |
| Dak phosphatase | EAN08621 | 3 | IGEGPTVDSTFDYEEFR  SVTEISASQFQEMVQAGANR  LQHETILEHDEEVNEFAK  NIFMAADQAAEVADIPVAVVPSR | Cyt | Ext |
| UDP-N-acetylglucosamine diphosphorylase | EEI59134 | 3 | HADVGPYAHLRPK  NASATILTAQAENPTGYGR  NSSIAAGSTITDNIPEYALAIAR | Cyt | Cyt |
| Aminoglycoside phosphotransferase | EAN10527 | 3 | VLHFER  VSGFIDLGR  LVGENENLYLK  TEKPEEELVFSHGDLGDSNIFVK  ELYDFLKTEKPEEELVFSHGDLGDSNIFVK | Cyt | Cyt |
| Ribosomal protein L19 | EAN10503 | 3 | GAGISETYTVR  RGAGISETYTVR  SDIPAFRPGDTVR  MNPLIEELTKEQLR | Cyt | Cyt |
| Trigger factor | EEI60214 | 3 | SMDEFLNNMQR  VLTEDMLEHDVTMKK  ELVETKEKAADDAKDEAAIR  MAVENAEIVELPHVMVHDEVHR  LGQYKDLVVEKQDREVTDADVDAR  QGISPEMYYQLTGSTEEDLHKQFEGEAEMR  QGISPEMYYQLTGSTEEDLHKQFEGEAEMRTR | Cyt | Cyt |
| 50S ribosomal protein L2 | EEI61156 | 3 | SANIALVHYEDGVK  ATIGSVGNEQHELINIGK  NKDNVVATVQTIEYDPNR  VGNALPLENIPVGTVIHNIEMKPGK | Cyt-Mem | Ext |
| Ribosomal protein S8 | EAN09416 | 3 | VMTDPIADFLTR  DVEYIEDDKQGVIR  EGFVRDVEYIEDDKQGVIR  VLNGLGIAIISTSEGVVTDKEAR | Cyt | Cyt |
| Ribosomal protein S9 | EAN10220 | 3 | ALLEVDPDFR  AQVQYIGTGR  DVEEYIPHADLR  KDVEEYIPHADLR  ITVNKKDVEEYIPHADLR  DVEEYIPHADLREVINQPFAVTETK  GAYDVFVNVNGGGYAGQSGAIR | Cell Mem-Cyt-Extracell. | Cyt |
| Carbamoyl-phosphate synthase, large subunit, glutamine-dependent | EAN09250 | 3 | VAELWEINEEAVR  EAVEHGIPLFTSLDTADAIVR  AMEIVENQRDLEDYMEHAVK  QNGFTDKKVAELWEINEEAVR  TAKQNGFTDKKVAELWEINEEAVR | Cyt | Cyt-Mem |
| Glutamyl-tRNA(Gln) amidotransferase B subunit | EAN09523 | 3 | VLLSGGEIQQETR  SPEEAYAYLEALR  QGTPLIEIVSEADMR  NMHGIGGYSYVDLNR | Cyt | Cyt |
| Pyruvate kinase | EAN09350 | 3 | TTVQGTTEADFGR  AGYIKFEVGDQTR  FNFSHGDHEEQLSR  YRPDADILAVTFDER  TGKDVGILLDTKGAEIR  GDMGVEIPAELVPMVQKR  IESQEGIDNIDEIIKVSDGVMIAR  KAQDVLDIREILEEKDMTHVQIFPK | Cyt | Cyt |
| Cell division protein FtsA | EAN10731 | 3 | INYGDAYPER  GIIVDIDKTVQAIQR  QIVSILPQDFTVDGFEGIKDPR  TSPDEEFPVDVIGQSEPVKVDER | Cyt | Cyt |
| Ribosomal protein L16 | AAG02220 | 3 | GAPEGWVAPVKR  IMFEIAGVPEEVAR  MGSGKGAPEGWVAPVKR  GKIMFEIAGVPEEVAR  GGKEVAFGEYGLQAVDSHWITNR | Cyt | Cyt |
| Phosphoglycerate mutase 1 | EEI61144 | 3 | YAMLDKR  YGDEQVHIWR  YGDEQVHIWRR  DIPGGENLKVTLER  ETAEKYGDEQVHIWR  ALPFWQDEIAPALLDNK  ETAEKYGDEQVHIWRR  KIKEAGIEFDVAYTSVLTR  SYDTLPPLMEETDEGSAANDR  YAMLDKRDIPGGENLKVTLER  SYDTLPPLMEETDEGSAANDRR  RSYDTLPPLMEETDEGSAANDRR | Cyt | Cyt |
| Arginine deiminase | EAN09542 | 3 | MLDESHIASNAVR  LAAEAIDAGNVKEQFLNK  AALGLDDLVLIPTGNGDEIVAPR  EQWNDGSNTLAIAPGVVVTYNR  LLFDDIPYLPIAQEEHDNFAK  SSSLYDLSADDDYPFYMDPMPNLYFTR | Cyt | Cyt |
| Ribosomal protein L17 | EAN09429 | 3 | LFNDIAPR  DLTTDLLINER  LRDLTTDLLINER  RGDGAPMVVLELV  AMLRDLTTDLLINER  KAMLRDLTTDLLINER  NEVASVREENEDIVIESALQK  QAAAFVRNEVASVREENEDIVIESALQK | Cyt | Mem |
| Ribosomal protein L1 | EEV43329 | 3 | NVTITTTFGPGIHVDQASF  VSFDDEKLVENFAAIHDVLLK  IQNGWFDFDVVVATPDMMATVGR  NLVEVPMVGSTIPHEVIGAFSGGR | Cyt | Cyt |
| Sigma 54 modulation protein/ribosomal protein S30EA | EAN08596 | 3 | VEVTIPLPYLVLR  GENIEVTEAIRDYVEK  AEETSPDLYASIDLVVDKLER  ETGMNTADATALFNEEDENENDSELDIVR  SRETGMNTADATALFNEEDENENDSELDIVR | Cyt | Cyt |
| Ribosomal protein L6 | EAN09417 | 3 | ALELIGVGYR  GANKEEVGELAANIR  MNIEGNEVTFTRPNDSKEMK  GANKEEVGELAANIRGTRPPEPYKGK  VVVIPEGVTVTQDGNNVTVKGPKGELTR  TFSADIKMNIEGNEVTFTRPNDSKEMK  IGNKVVVIPEGVTVTQDGNNVTVKGPKGELTR | Cyt | Cyt |
| Ribosomal protein L5 | EAN09414 | 3 | FNYSSVMQTPKVEK  MYEFLDKLVSVSLPR  EQLIFPEVDYDLVDKVR  NLDKAVEELTLITGQKPMITK  GNYTLGIKEQLIFPEVDYDLVDKVR  GMDIVIVTTANSDEESRELLTQLGMPFQK | Cyt | Cyt |
| Tyrosine--tRNA ligase | EEI60792 | 2 | NIIDELTWR  MNIIDELTWR  VLQTMEQVQHNVDALSNQMR  KTSPFEFYQFWLNQDDRDVVK | Cyt | Cyt |
| Ribosomal protein S15, bacterial chloroplast and mitochondrial type | EAN08911 | 3 | NEIINEYAR  KNEIINEYAR  HEGDTGSPEVQIAVLTEEINHLNEHAR | Cyt | Cyt |
| ABC transporter | EAN08750 | 3 | LISSYSHGMKKK  KEEQIKELQEFIAR  DTTKEFDTDLTILDWLR  VSEQYGLSVNPDAYVR  VLSGDLQPSTGSVTMGPDER | Cyt | Mem |
| Phosphoribosylamine--glycine ligase | EEI60577 | 2 | VIEFNAR  MNILVIGAGGR  FGDPETQVVLSR  EALDAAEQMLNQHR | Cyt | Cyt-Mem |
| Peptidylprolyl isomerase | EEI59596 | 3 | GGGTPWLDFR  TNRGDITVQLFPELAPK  GPQDRPVHDVVIETIEISE  HTVFGHVLEGMDTVDEIANVQR | Mem | Ext |
| Uracil phosphoribosyltransferase | EEI59351 | 3 | EVILIDDVLYTGR  AAMDAVMDFGRPR  AEEILVEMQELDGQDR  LKQLEDIDIPVGELDITLYR  LNEHGYIVPGLGDAGDR  VGHIGLYRDEETLEPHEYFVK  LNEHGYIVPGLGDAGDRLFGTK  QLFVVDPMLATGGSAIMAIDALKER  VGHIGLYRDEETLEPHEYFVKLPEDIDAR | Cyt | Cyt |
| [acyl-carrier-protein] S-malonyltransferase | EEI61246 | 2 | ETIAGLLEQQVMQPVR  KETIAGLLEQQVMQPVR | Mem | Mem |
| Ribose-Phosphate pyrophosphokinase | EAN10234 | 3 | LVVTDSIYLPEDR  IHENTPMSPLFR  IHENTPMSPLFRLEEKEFE  KIDKIDEVSVGGLMGDAIKR  GIKGDDVVVVSPDHGGVTR | Cyt | Cyt |
| UDP-N-acetylmuramate--alanine ligase | EAN08897 | 2 | AIFAYGDDAYLR  ANVPIYYYGVTENDDIQAR  QKYPDKEIIAVFQPHTFTR | Cyt | Cyt-Mem |
| Conserved hypothetical protein 92 | EAN09300 | 2 | APQAAGIIHSDFER  DYVVQDGDVMLFR  AGAEAANYPFATIDPNVGMVEVPDWR | Cyt | Cyt |
| 50S ribosomal protein L14 | EAN09412 | 2 | FDENAAVIIR  FDENAAVIIRDDK  FDENAAVIIRDDKSPR | Cyt | Cyt |
| Amidophosphoribosyl transferase | EAN09783 | 2 | LQQLTGEAAIGHVR  DLGLLSEVFKDQR  KLQQLTGEAAIGHVR | Cyt | Cyt |
| low affinity penicillin-binding protein 5 | EFF35784 | 3 | VSLTTQEAAR  YQNIYGAADVK  FATGYAPGSTFK  DSSWGSYQVTR  AYEENPEQPFISR  SGLEMAFDKDLR  ISGLEMAFDKDLR  NVIGETALQTIVPDLR  IIDGATPELPAGATIQEVDGR | Ext | Ext |
| Ribosomal protein S6 | EAN09585 | 3 | INDDIIRHMIVKEEA  YEIMYIIRPNIDEEAK  VSSPSSANAVNEFDR  FAYEMNGYREGIYHIVK  VSSPSSANAVNEFDRLAK  MENTKYEIMYIIRPNIDEEAK  VSSPSSANAVNEFDRLAKINDDIIR | Cyt | Cyt |
| Ribosomal protein S13 | EAN09426 | 2 | LKVEGDLR  ILADAGVSEDVR  KILADAGVSEDVR  AEVDKLKVEGDLR | Cyt | Cyt |
| Ribosomal protein L31 | EEI61448 | 3 | VEVTSDSHPFYTGR  TSQETVEWEDGNTYPVIR  MKENIHPDYHPVVFMDSTTGFK  MKENIHPDYHPVVFMDSTTGFKFLSGSTK  TSQETVEWEDGNTYPVIRVEVTSDSHPFYTGR | Ext | Cyt |
| Hypoxanthine phosphoribosyl transferase | EAN10267 | 2 | ILISQEEIQVR  DLLIVEDIIDSGR | Cyt | Cyt |
| Hypothetical protein EFZG_01592 | EEW66026 | 2 | VLNAYVDEDHQVR  SEMIVQDFIPGDDSNMR  IVNVEVIPGFDKDPVFIETMLR | Cyt | Mem |
| 50S ribosomal protein L3 | EFD10712 | 2 | VTIQNLEVVR  MGGNRVTIQNLEVVR  TMETDGYEAIQVGYQDMR | Cyt | Cyt |
| Ribosomal protein L29 | EAN09410 | 3 | QLKEELFNLR  FQLATGQLENTAR  ELTTAEMLDQEKQLKEELFNLR | Cyt | Cyt |
| HDIG | EAN10225 | 2 | KAKNLLSLAIQR  VMVKPEEISDLDAVR  SLENISNSFAGVESSYAVQAGR | Cyt | Cyt |
| Peptidase S1, chymotrypsin | EAN09870 | 2 | SEYANSVTSGIISSLNR  VGEPAIAIGSPLGSEYANSVTSGIISSLNR | Ext-Mem | Ext |
| Enolase | EAN08760 | 3 | YNQLLR  SIITDVYAR  EAGYTAVVSHR  GNPTIEVEVYTESGAFGR  AVDNVNNIIAEAIIGYDVR  GMVPSGASTGEYEAVELR  GMVPSGASTGEYEAVELRDGDKAR | Cyt | Cyt |
| Formate acetyltransferase | EAN08939 | 3 | LREEITEQYR  IITGLPDAYGR  MEQWNGFKGTK  THNQGVFDAYTAEMR  SNGYTPNDELTHIFTDWR  LLHTLTNMGPSPEPNLTVLYSEHLPEGFR  EAGKPLAPGANPSYNAEQSGLLASLNSTAK | Cyt | Cyt |
| Basic membrane lipoprotein | EFF34523 | 3 | WGKDQGLSR  GEVIDRFDAGFK  VLNQYAGDFSAPDKGR  SFNQSAWEGLEKWGKDQGLSR  VWVIGVDRDQSDEGEYTLNGEKK  SIAQGMYAQNADIIFHASGGTGNGVFQEAK | Ext | Mem |
| Ribosomal protein L11 | EEI59185 | 3 | MEDLNAANVEAAMR  TADQAGLIIPVVISVYEDR  EIAELKMEDLNAANVEAAMR  VSSDQVREIAELKMEDLNAANVEAAMR | Cyt | Cyt |
| Adenylosuccinate lyase | EAN09788 | 3 | NVDVIFKR  IREHASFDIDR  NMSATFGLIYSQR  QLGIRPQEISTQVLPR  GHMVTAYENVTLWHER  YTRPEMGNIWTEENKYR | Cyt | Cyt |
| ATP synthase F0 | EEV49775 | 3 | AQADITVER  MEQEREQQLLASR  ELSPEMHESLINQYIEGLGSSNETR  ILNKELSPEMHESLINQYIEGLGSSNETR | Mem | Mem |
| D,D-dipeptidase | ACU27885 | 2 | FDFMDER  GSAIDLTLYR  YATWDNFTGKPVDGYEVNR | Ext | Ext |
| Ribosomal protein L13, bacterial and organelle form | EAN10221 | 3 | WYVVDATDVPLGR  LNVYGGAEHPHAAQQPEVLDITNLI | Cyt | Cyt |
| Glycyl-tRNA synthetase, alpha subunit | EAN09379 | 2 | AIGPEPWNAAYVEPSR  YSFEVSDQDLLLSNFDRFEK | Cyt | Cyt |
| ATP-dependent Clp protease, ATP-binding subunit ClpC | EAN09176 | 2 | LLELEGLLHER  IQVDEPTPEEAEVILQGLR | Cyt | Cyt-Mem |
| 30S ribosomal protein S11 | EAN09427 | 2 | SLQATGLEVTAIR  STPFAAQMAAETATK | Cyt-Ext | Cyt |
| GroEL | EAN09274 | 3 | NVTAGANPLGIR  NVTAGANPLGIRR  EGLKNVTAGANPLGIR  EGLKNVTAGANPLGIRR  AAVEEGMVSGGGTALVNVISK  VVVDKDNTTIVEGSGEKEAIEAR  ALEEPIRQIAENAGYEGSVIVDKLK  EAIAQVAAVSSGSDKVGHLIADAMEK  SYGSPLITNDGVTIAKEIELEDHFENMGAK | Cyt | Cyt |
| Vancomycin resistance protein | AAA24789 | 2 | QGAFLINTGR  MPNVIITPHTAYYTEQALR  MPNVIITPHTAYYTEQALRDTVEK | Cyt | Cyt |
| RNA polymerase beta subunit | AAP21847 | 2 | EIPNVGEDALKDLDEMGIIR  QLGIHVATPVFDGASDEDVWETVR | Cyt | Cyt |
| DNA directed RNA polymerase | EEV51758 | 2 | LGIQAFEPVLVQGR  TFHTGGVAGDDITQGLPR | Cyt | Cyt |
| tRNA (5-methylaminomethyl-2-thiouridylate)-methyltransferase | EAN10581 | 3 | VFEYFLAEYR  TMFPLGGMEKSEVR  LLDDNRAEVVFDEPVR  VAAQIGIPYYSVNFEKEYWDR | Cyt | Cyt |
| Ribosomal protein L23 | EEI61155 | 3 | YTFEVDTR  KYTFEVDTR  QAVEAAFDVK  MNLLDVIKRPVITEK  SMLAMDDKKYTFEVDTR  RKAIVTLTEDSKEIQLFEAAE | Cyt | Cyt-Mem |
| Prokaryotic transcription elongation factor GreA | EAN09082 | 3 | ITTIENMIR  GFGDLSENSEYESAKDEQAFVEGR | Cyt | Cyt |
| GMP synthase (glutamine-hydrolyzing) | EEI61129 | 2 | ELGTQLGMPDSIVWR  AIGDQLTSIFVDHGLLR | Cyt | Cyt |
| ABC transporter, ATP-binding protein | EFF31524 | 2 | ALELLEQVGIPNPAR  IPWIPASDHEEEPILHEVAPNHLVR | Cyt | Mem |
| PpiC-type peptidyl-prolyl cis-trans isomerase | EFF34785 | 3 | QSLAFQEGLK  SAGYTEQTFK  NNLAFEAGLK  LESSNQSLVQR  SFHPEVEAQIIK  QLGDSFDSQLK  QSLAFQEGLKK  VIGEVLKDANVK  SFHPEVEAQIIK  SLGDTFESQLEAAGYTKDTYKEYIR  GGTITVSDFYDEAKLESSNQSLVQR | Ext | Ext-Mem |
| Aspartyl-tRNA synthetase bacterial/mitochondrial type | EEV48334 | 2 | GIEVTLPFPR  TPPFQIEDDQAISDEIR+  ATDAKPGDLLMFGADKSEIVAAALGAIR | Cyt | Cyt |
| Beta-lactamase-like:RNA-metabolising metallo-beta-lactamase | EAN10342 | 2 | NYDPEELIILETGR  FDQTAIPMYQTDYAR | Cyt | Cyt |
| Enoyl-(acyl carrier protein) reductase | EEI60436 | 2 | YLAAEFSPK  QGAEIIYTYQNER | Mem | Cyt |
| Phosphoenolpyruvate-protein phosphotransferase | EEI61237 | 3 | ISLSEQGDEMFR  TMDIGGDKELPYLQLPHEMNPFLGYR | Cyt | Cyt |
| Hydrolase | EEV45310 | 3 | VMAGDWTLEYMR  MMMIDEPEILDAAIAR  VVPLVSDADMHPLYIR  NLPTEEVAAQTWTNAHR  SVVFDVDDTMYDQQQPFR | Cyt | Cyt |
| UMP kinase | EFD09842 | 2 | AILGENIGTTVR  GQIGAQMGMER | Cyt | Cyt |
| CTP synthase | EAN10444 | 3 | GLGIQPNILVVR  TAEAYDGADVVQER  QLFEEQGLVFSGVSPDNR | Cyt | Cyt |
| Aspartate-semialdehyde dehydrogenase, USG-1 related | EEV45096 | 2 | ALQEAFGIQR  VLEEYDFPVGR  VNYSTYQAVSGSGQK | Cyt | Cyt |
| Initiation factor 2:Small GTP-binding protein domain | EAN09509 | 2 | DKEAGPATPVEITGLNDVPQAGDR  GPVSTLLVQEGTLHVGDPIVVGNTYGR | Cyt | Cyt |
| Exopolysaccharide synthesis protein | EAN09384 | 2 | SGPKPPNPSELLSSPR  TPVENLSILPSGPKPPNPSELLSSPR | Mem | Mem |
| FAD-dependent pyridine nucleotide-disulphide oxidoreductase:Pyridine nucleotide-disulphide oxidoreductase dimerisation region | EAN09169 | 2 | NHPEAEVTVYER  ESATLNDLEVQATVFEDNYRPEFMPTTEK | Mem | Cyt |
| LysM domain protein | EFF34034 | 2 | AGITTDQLFELNGLDPNNFMLYPGQELR  HQSTENNETQEPWEQPIYDTDDEVSSR | Ext | Ext |
| Acetyl-CoA carboxylase, biotin carboxylase | EAN10065 | 3 | HIEVQILGDR  VLEESPSVVISQTKR  AEAIHPGFGFLSENSR | Cyt | Mem |
| Pyruvate carboxylase | EFF37273 | 3 | TTFIDSTATLFEFPR  TVGIYAEEDEYSVHR  ITTEDPLNNFLPDTGKIDTYR  YIGEITVNGFPGIESGEKPFYEEPR | Cyt | Cyt-Mem |
| Peptidase U32 | EAN09175 | 2 | YGLFDMPFGQER  VYVAANMVTHEGNEDGAGEFFR  ELATGFYYNTPTENEQLFGER | Cyt | Cyt |
| Methionine aminopeptidase | EAN10665 | 2 | ALYIGIEQAQVGNR  IGDIGHAIQTYVEGENLAIVR | Cyt | Cyt |
| Phosphoribosylformylglycinamidine synthase I | EAN09785 | 2 | FSTIMEEVIR  HDESSLEGFDGVLLPGGFSYGDYLR | Cyt | Cyt |
| ATP-dependent Clp protease ATP-binding subunit ClpX | EEV41455 | 3 | GIIYIDEIDKIAR  LLSLDNTELEFEPEALR | Cyt | Cyt |
| Glycine--tRNA ligase | EEI59843 | 2 | FLGEDVTFAHAK  NDQGMLLPHFISAR  FLDENQLTYDTIETFSTPR | Cyt | Cyt |
| Acetyl-CoA carboxylase carboxyl transferase, beta subunit | EAN10066 | 2 | AEFLLSHGFVDQIVPR  RVIEQTIKQELPEDFQK | Mem | Cyt |
| 6-phosphofructokinase | EAN09351 | 3 | LSEFGDYHTR  TFVIEVMGR  RLDVADVGDKIQR  YPEFATEEGQLK  YPEFATEEGQLKGIEQLKK  IGILTSGGDAPGMNAAVR  GGTFLYSARYPEFATEEGQLKGIEQLKK | Cyt | Cyt |
| L-lactate dehydrogenase | EAN10259 | 3 | QALAELVDVDAR  FRQALAELVDVDAR  VIEIPLSDSEQDRMAASAK  NNPEIDEEAMVNLFFGVR QGIQKVIEIPLSDSEQDRMAASAK  IYSATYADAHDADLVVITAGAPQKPGETR  KIYSATYADAHDADLVVITAGAPQKPGETR AILDDENAVLPLSVFMNGEYGLNDIYIGAPAVINR | Cyt | Cyt |
| Ribosomal protein S10 | EFF30339 | 2 | TGASVSGPIPLPTER  RTGASVSGPIPLPTER | Cyt | Cyt |
| Valyl-tRNA synthetase, class Ia | EAN09797 | 3 | GEYIINWDPQAR  WMENVHDWVISR  FVSNAPEEVVEAER  LHLGHAWDTTLQDMLIR | Cyt | Cyt |
| Phosphoribosylaminoimidazole carboxylase | EEV41069 | 2 | TTLPVIGVPVQSR  TPDLMFEYAQNAR | Cyt-Mem | Cyt |
| Protein of hypothetical function DUF28 | EEI61262 | 2 | YEGFGPSGSMVIVDTLTNNVNR | Cyt | Cyt |
| Translation elongation factor Ts | EEI60042 | 2 | DVAMHVAAINPR  RFEVVEKDDNAAFGGYLHMGGR | Cyt | Cyt |
| Beta-ketoacyl-acyl-carrier-protein synthase II | EEI61248 | 3 | GSIPFDENR  FGVIIGSGIGGLPTIENQVIR  NGFVMGEGAGVLVLESLEHAQAR | Cyt-Mem | Cyt |
| Xylose import ATP-binding protein XylG | EFF38008 | 2 | KITEHGVGHVPEDR  VSEQYGLSVNPDAYVR | Cyt | Mem |
| Transketolase | EEV41175 | 3 | MFDKIDQLGVNTIR  IVWDVSHQSYGHK  EFTDSVPVGQLYER  VAMETTDAPTILVLSR  EVYGWEYPDFTVPEEVAAR  YVGTEGATITIDHFGASAPGTK  EDMISMLDWAFEQTAQPVAIR  MAKEVYGWEYPDFTVPEEVAAR  FHQTMIEEGQKAEDAWNEMFANYKK  AYDQLSHDLAINENPVIILVGGGTISASDR  YLAPATKEDMISMLDWAFEQTAQPVAIR  LAAIQHTPVVYVLTHDSVAVGEDGPTHEPIEQLASVR | Cyt | Cyt |
| Regulatory protein, TetR | EAN10570 | 2 | TIDSILALDNPEIIR | Cyt | Cyt |
| Threonyl-tRNA synthetase | EAN10081 | 2 | IPYQLVVGDKELEDATVNVR  FDLTYVGEDGENTHRPVVIHR  LSYRDPNNTDKYFDDDAMWEK | Cyt | Cyt |
| Universal stress protein family | EEW64499 | 2 | EIPEDNQVDLIMLGATGLNAVER | Cyt | Cyt |
| Nucleoside 2-deoxyribosyltransferase | EAN09913 | 3 | ALEENKTVTSFYSPR  SQIYLAGPFFSEEQIDR  IEKALEENKTVTSFYSPR  SSDQAVREYDFETLPVETYVGEYL | Cyt | Ext |
| Asparagine synthase | EAN09487 | 2 | HLPEEWSNR  AWLKEEHGYEQVK | Cyt | Cyt |
| Crossover junction endodeoxyribonuclease | EEI59678 | 2 | INEAEEEFGFDR  IKELFSLPVDYQDER | Cyt | Cyt |
| Putative ABC transporter | CAD21830 | 2 | YQPDDLRPALK  GVDVPSEYMTEER  FEQVDFTYQPNTPFEQR | Cyt | Mem |
| 3-dehydroquinate synthase | EAN09101 | 3 | VVEEDVFDQGNR  SLEQAAELYDFLADNDFTR | Cyt | Cyt |
| Cell-division initiation protein DivIVA | EAN10725 | 2 | DYEELTQR  ADELVANAEKR  HSEEKLEYFNELK  GYNQDEVDDFLDLVVR | Cyt | Cyt |
| s-ribosylhomocysteinase | EEW61267 | 2 | FKQPNKEHMDMPSLHSLEHLTAELIR | Cyt | Cyt |
| Phosphoribosylformylglycinamidine synthetase PurS | EEW65761 | 2 | MGYETIEDIR  LLANVNMETYR | Cyt | Cyt |
| Ribosomal protein S18 | EFF27944 | 2 | KVDYIAANHIEYIDYKDIELLKR | Cyt | Mem |
| Protein of unknown function DUF322 | EAN10750 | 2 | GTFASNVTELLGR | Cyt | Cyt |
| hsp70-like protein | EEV61248 | 3 | FQLTDIPAAPR  GFAEDYLGEKVEK  AKFDELTADLVER  AVITVPAYFNDAQR  TTPSVVSFKNGEIQVGEVAKR  IVNEPTAAALAYGLDKTDRDEK  QALKDAGLSQSEIDEVILVGGSTR | Cyt | Cyt |
| Hypothetical protein HMPREF9526_02551 | EFR70419 | 2 | SGENGIFER  LLNQFRPSLDGVR  INVPIDDNFNTVLK  INVPIDDNFNTVLKR | Cyt | Ext |
| Predicted protein | EEV46337 | 2 | SALEALYTSVK  NMTDQNYFLCR  QKELAWLNDYR  DHHYTASWDEVK  NYGWTYEGPAWR  WGNYEGPAWEAPTSGGHLVYR | Ext | Ext |
| Ribosomal protein L15 | EFS07584 | 2 | LGFEGGQTPLFR  SGGGVRLGFEGGQTPLFR | Cyt | Cyt |
| conserved hypothetical protein | EEV46664 | 2 | SDNLFLEVQPEVFKQVPR | Cyt | Ext |
| Ribosomal protein L21 | EAN08779 | 2 | VEVGQAIYVEK  VGAPTVAGATVEGTVEK | Cyt | Cyt |
| ClpE | EFF23737 | 3 | VEANVGFGAAR  AQNDPFGFGSLDDLFR  MQPVRVDEPTVEETISILK QLTEEETPVITEKDMEKIVEQR  MQPVRVDEPTVEETISILKGLQK  YEDYHHVKYTDEAIEAAATLSNR | Cyt | Cyt-Mem |
| Pyridine nucleotide-disulfide oxidoreductase | EFR66605 | 2 | AHGFTLWSFEDSVR  VEPEAIQYDLQHLFAR | Mem | Cyt |
| Ribosomal protein L7/L12 | EEV43331 | 2 | ALNIENIVAELK  LEEVGASVTVK  AVVDGAPAPVKEGVSKEEAEELKAK | Cyt | Cyt |
| ribosome biogenesis GTP-binding protein YsxC | EFR69403 | 2 | QYPDTQLPEIALAGR | Cyt-Mem | Cyt |
| Chromosomal replication initiator protein, DnaA | EAN09579 | 2 | QIAMYLAR  YTFDTFVIGK  DLSPASYNTWIETANPR | Cyt | Mem |
| GTP-binding protein | EEV47685 | 3 | QYPDTQLPEIALAGR  NPAPEIAENGEPGQER  LFQMTNFDHDETVMR  VILHVIDMSGMEGRDPYEDYLAINK  FKEQLNKEKEDEFADDIPVFPISGVTR | Cyt | Cyt |
| Acetyl-CoA carboxylase, biotin carboxyl carrier protein | EFF30077 | 2 | EGQFELYMNK  ELVSQFDQSSLTEFDLR | Cyt | Cyt-Mem |
| Dihydroorotate oxidase | EAN09059 | 2 | EGPEIFSR  LKPEIQIIGTGGIR  EGFGGIGGEYIKPTALANVR | Cyt | Cyt |
| Histidyl-tRNA synthetase | EAN09125 | 2 | TPIFEHYEVISR  LLFRDYQYNEMR  GTNDILPGESEKWQFVEETAR | Cyt | Cyt |
| DNA-directed RNA polymerase, delta subunit | EFR69072 | 2 | SELSMIEVAHAILEQR | Cyt | Ext |
| Gamma-glutamyl phosphate reductase GPR | EAN10188 | 2 | LTEYLDVLIPR  YIIYGDGQIRE  IASLDDPIGKVDEMWTNTDGLR | Cyt | Cyt |
| ATP synthase alpha subunit | AAM49626 | 3 | IMEVPVGEALIGR  ARPVEAMAPGVMQR | Cyt | Cyt-Mem |
| DNA-binding protein II | EEI60701 | 2 | KGRNPQTGEEIEIPASK  GRNPQTGEEIEIPASKVPAFKPGK  KGRNPQTGEEIEIPASKVPAFKPGK | Cyt | Cyt |
| D-alanyl-D-alanine carboxypeptidase | EFF35669 | 2 | IAPEYEAFAK  WIADNAAQYGFIVR  AAEAAGFPLVMVSAYR  YPEDKTELTGIQYELWHIR | Mem | Mem |
| Fibronectin binding protein A | EEI60406 | 2 | DGELLTTFLTQVPR  IHQPYENEIVLVIR | Cyt | Cyt-Mem |
| Cell division ATP-binding protein FtsE | EEI59752 | 3 | IEMKDVMKK  VFPSELSGGEQQR  IIRDQAEGEYGYDD | Cyt | Mem |
| Ribosomal protein L27 | EFS07335 | 2 | ADGQTVTGGSILYR  RADGQTVTGGSILYR | Ext-Mem | Ext |
| Sodium-transporting two-sector ATPase | EAN10388 | 2 | ILQEEQQLNEIVR  FLGHPLELGVSEDMIGR  LEEMPGDEGYPAYLGSR | Cyt | Cyt-Mem |
| Cold-shock protein, DNA-binding | EAN09358 | 2 | TLEEGQAVTFDVEEGQR TLEEGQAVTFDVEESDRGPQAANVVK  TLEEGQAVTFDVEEGQRGPQATNVTKA | Cyt | Cyt |
| Polyribonucleotide nucleotidyltransferase | EFF36260 | 2 | DGLVHISQLANDR  KLDEIRPLSSEVSLLPR  ALAQVIPSEEEFPYTIR | Cyt | Cyt |
| Adenylosuccinate synthetase | EAN09591 | 2 | IADLLDKEIFEER  VGDGPFPTELFDETGEQIR | Cyt | Cyt |
| Ribosome recycling factor | AFK59307 | 2 | QQKNGDITEDDLR  AIMASDIGISPTNDGNVIR | Cyt | Cyt |
| Putative metallophosphoesterase YsnB | EFF36717 | 2 | ILFLNPGSISQPR  AHQAVEELAFSFSR | Cyt | Cyt |
| Iron-containing alcohol dehydrogenase | [EAN09662](http://www.ncbi.nlm.nih.gov/protein/68195206?report=genbank&log$=prottop&blast_rank=2&RID=P6T1VURV01R) | 2 | YDYFRADTDYADIAK  MLIAELEGVGPDYPLSR | Cyt | Cyt |
| Heat shock protein Hsp33 | EEI59163 | 2 | ALAHDGFVR  AYAVQATNTVAEAQR | Cyt | Cyt |
| Ribosomal protein S19, bacterial and organelle form | EAN09406 | 2 | HVPVYIQEDMVGHK | Ext-Mem | Cyt |
| Thioredoxin | EAN10401 | 3 | EQLRQMIDQYL  MQAPILDQLEQEYDEEEFR  AVGVHSKEQLRQMIDQYL MQAPILDQLEQEYDEEEFRIAK  DGQVVEKAVGVHSKEQLRQMIDQYL | Cyt | Ext |
| Glutamine transport ATP-binding protein GlnQ | EFF29922 | 2 | VMFIDDGNFLEDGTPQQIFENPQNER | Cyt | Mem |
| Phosphoribosylformylglycinamidine cyclo-ligase | EFR78857 | 2 | AGVDVEAGYEVVER  GAAHITGGGFVENIPR | Cyt | Cyt |
| Ribonucleoside-diphosphate reductase, alpha subunit | EAN09926 | 2 | LFEDSFSYSNQLGQR  IYYPAPYLSNETIPYYTSAYDMDMR | Cyt | Cyt |
| dTDP-glucose 4,6-dehydratase | EEI61370 | 2 | QVTNILSGIRPK  EELGWQPEFTNFR | Cyt | Cyt |
| NAD-dependent epimerase/dehydratase | EAN10656 | 2 | LREELGWKPEFTNFR | Ext-Mem | Cyt |
| Ribosomal protein L25, Ctc-form | EAN08602 | 2 | EHGANTVITMDIDGQK  SGGVLAQNLYTVVVSATPDKLPER | Cyt | Cyt-Mem |
| dTDP-4-dehydrorhamnose reductase | EAN10655 | 3 | AVEEILEDYYIIR  NVEYEVDDQTNPLNEYGR | Cyt | Cyt |
| Pur operon repressor | EAN10232 | 2 | ITEGSTVSVNYVSGSSER  LVDMTQYLLNHPHELISLTSFAER | Cyt | Cyt |
| H1-transporting two-sector ATPase, delta/epsilon subunit | EAN09185 | 2 | MEYHELNPLIR  ELELLSNETFER  HAPIIVPLTIDEVR | Cyt | Cyt |
| Metallo-beta-lactamase superfamily protein | EEV42569 | 2 | TDLYTGNLEQLLHSIR | Mem | Ext |
| Peroxiredoxin | EFS06595 | 2 | IFDVLDEEQGLAQR | Cyt | Cyt-Ext |
| Signal recognition particle-docking protein FtsY | EAN0850 | 2 | AGAIDQLVVWGER  GNAGGDPAAVVFDALER | Cyt | Cyt-Mem |
| Macrolide export ATP-binding/permease protein MacB | EHM36381 | 2 | STLLNILSTLDKPTDGHIR | Mem | Mem |
| Proline--tRNA ligase | EEI60036 | 2 | AGYEVLVDDRNER  QVSAGIYSYLPLANR  SVLFIADEQPVLVLVR | Cyt | Cyt |
| rpsA, 30S ribosomal protein S1 | EEI60699 | 2 | GGLVVDVGVR  KVWEDIERDFQEGK | Cyt | Cyt |
| 6-phosphogluconate dehydrogenase, decarboxylating | EAN09346 | 2 | ATYTIEEFVESIEKPR  VDKEGIFHYSWYHEE  ATYTIEEFVESIEKPRR | Cyt | Cyt |
| Inosine-5'-monophosphate dehydrogenase | EFS05230 | 2 | DRYFQGSVNEANK  LPIVDNEGRLSGLITIK | Cyt | Cyt |
| Dihydroorotase multifunctional complex type | EAN09248 | 2 | GQLITPGLVDVHVHLR  KAEELGLPGILSVTESSQIAR | Cyt | Cyt |
| Dihydroorotase | EFF32592 | 2 | NMTTPAEIWIEDGRIK  GQLITPGLVDVHVHLR  KAEELGLPGILSVTESSQIAR | Cyt | Cyt |
| Hypothetical protein HMPREF9526_02845 | EFR70128 | 2 | QLVDRYPSNYHDVEVQIQK | Cyt | Mem |
| Ribonucleotide-diphosphate reductase subunit beta | EAN09926 | 2 | LTEQFWLDTR  IPLSNDLDDWR | Cyt | Cyt |
| Hydrolase | EEV43078 | 3 | VMAGDWTLEYMR  VVPLVSDADMHPLYIR  MMMIDEPEILDAAIAR  NLPTEEVAAQTWTNAHR  SVVFDVDDTMYDQQQPFR | Cyt | Cyt |
| Translation initiation factor IF-1 | EAN09424 | 3 | ILPGDKVTVELSPYDLNR  ILPGDKVTVELSPYDLNRGR | Cyt | Cyt |
| Lysine--tRNA ligase | EFF60521 | 2 | EGQIQIYVR  EFANAFTELNDPIDQR | Cyt | Cyt |
| DNA replication initiation control protein YabA | EEV51422 | 2 | SLYDGLNQLESELR | Cyt | Cyt-Mem |
| Aminotransferase | EEV49863 | 2 | HYQHNEIGYNYR  INIDTTGNIGIGHTR | Cyt-Ext | Cyt |
| CBS domain-containing protein | EEV41210 | 2 | IQLIEQELIQPETVLPR | Cyt | Cyt |
| Sporulation initiation inhibitor protein soj | EHM33068 | 2 | LYGDNLINTIVFR  KLYGDNLINTIVFR  GAADYIDYMEFFTER | Cyt-Mem | Cyt-Mem |
| Ribonucleotide reductase | EEI59656 | 2 | FGFPFLADYELR | Cyt | Cyt |
| Cold shock protein | EEI61403 | 2 | SLEEGQEVEFTIVEGAR | Cyt | Cyt |
| Conserved hypothetical protein | EAN11004 | 3 | SNIYDSANQIER  IIMTPVRDLYSE  SNIYDSANQIEREIR  SNIYDSANQIEREIRELPEFK | Cyt | Mem |
| Transcription antitermination protein NusG | EAN08510 | 2 | NTPGVTGFVGSHGAGSKPAPLLPEEVNHILR | Cyt | Cyt |
| Conserved hypothetical protein | EEV53316 | 2 | ASQLGISPEEVASLSQQASLTR | Mem | Mem |
| Putative sugar-binding domain protein | EEI59792 | 2 | MAIAGGKSKAKAIR  MIESVAPDIIEVLQER | Cyt | Cyt |
| Phosphoribosylglycinamide formyltransferase | EEI60575 | 2 | ETGVTIHYIDQGVDTGPIIR | Cyt | Cyt |
| CobB/CobQ-like glutamine amidotransferase domain protein | EFR71929 | 2 | VVQGQGNNGEDQSEGVIYR  IQGISALDHYTLSQENNR | Cyt | Cyt |
| DNA topoisomerase IV subunit A | EAN09573 | 2 | GQQITIDPTDFPIGDR  KIDTQVLVAQEDVIVSVTR | Cyt | Cyt |
| Phosphomethylpyrimidine kinase | EAN08768 | 2 | AIREEINVGHK  KLVPLAEVLTPNFYEAEK | Cyt | Cyt |
| 50S ribosomal protein L20 | EAN08876 | 2 | EQVMNSYNYAYR | Cyt-Ext | Mem |
| ATP-dependent RNA helicase | EEI61347 | 2 | RGVPVGSLASDQN  GLDITGLPYVVNAEVPLSEEAYLHR | Mem | Cyt |
| UDP-N-acetylmuramoyl-L-alanyl-D-glutamate-L-lysine ligase | EFD09395 | 2 | ETADLFQIPTITYGR  TVVLNHESDYFDLLR | Cyt-Mem | Cyt-Mem |
| GTP-binding protein YqeH | EEV54363 | 2 | HYNEIQDVSLTDDDFLR  RAYEAGIRPVDVLLTSAK | Cyt | Cyt |
| GntR family transcriptional regulator | ZP_05714436 | 2 | QISYFEDGTPFEYVR  RFADDVPICFEVASVPQK | Cyt | Cyt-Mem |
| Pyruvate decarboxylase | EAN09595 | 3 | GIIPDDYENFLGFAGR  FAFDAAAKSDRPVVIDVK  YSAEIQDPETISEVIANAYR | Cyt | Cyt |
| ATPase, ParA family protein | EFF35808 | 2 | GIDVLPADIALSSAER  GAEVYQALAKEVVSREEK | Cyt | Mem |
| Peptidase M29, aminopeptidase II | EAN10668 | 2 | NHLWEGAGSYNAR  REQFSALHYTAPGTDIIIGLPK | Cyt | Cyt |
| Transposase | AAD34803 | 3 | LGCQNFNSR  QFIKSCLAIITRNS  EIIGHSCGNKKDAQLVK | Mem | Ext-Mem |
| D-alanine--poly(phosphoribitol) ligase subunit 1 | ZP_05713501 | 2 | RLDEYGLLFYEG  IELGDIEHYLLQDNR | Cyt | Cyt |
| 3-oxoacyl-(acyl-carrier-protein) reductase | EEV43217 | 2 | NVFITGSTR  AGANIILNGRGEIPKEKIEEIEAFGVK | Cyt | Cyt |
| conserved hypothetical protein | EFF35541 | 2 | SSIDNMRFINAMK  SSIDNMRFINAMKLNK | Ext-Mem | Ext |
| 50S ribosomal protein L4 | AFK57682 | 3 | GGGVVFGPTPR  VLVVLEKGNDFAALSAR  VAENNLVAIEGLNFDAPK  NLPNVSVVTSDNVSVLDVVSNTK  SVLSEKVAENNLVAIEGLNFDAPK | Cyt-Mem | Cyt-Mem |
| HAD-superfamily hydrolase | EEV43241 | 2 | REIAFEILSQK  FETIVSAENVQR | Cyt | Cyt-Mem |
| AMP-binding enzyme | EFR74513 | 2 | WWEQVHELPR  RFQENVYGDWWDSGDYGFMD | Cyt | Cyt |
| Ornithine carbamoyltransferase | EAN09541 | 3 | QFDQAENR  MKESVFQGR  FGITEMEVTDEVFR | Cyt | Cyt |
| Glucose-1-phosphate thymidylyltransferase | EAN10659 | 2 | DTGATVFGYHVNDPER  RFGVVEFDENMQALSIEEKPAQK | Cyt | Cyt |
| Phosphoglucomutase/phosphomannomutase | EEV42904 | 2 | GVAIAYDSR  SPNPEEHSAFEYAIR | Cyt | Cyt |
| ATP-dependent protease ATP-binding subunit | ZP_03981246 | 2 | KLAEESGIIFIDEIDK  DLVENAIQIVEKEQYSR | Cyt | Cyt-Mem |
| Exonuclease VII, small subunit | EAN10746 | 2 | IVMQLEQGDVPLESALDSFKR | Cyt | Cyt |
| MreC | EAN10252 | 2 | KDGIEANMAVMSQK  IELLSSSNESSNHFPVR | Ext | Ext |
| acetyl-CoA carboxylase | EAN10067 | 3 | NFGSPHPEGYR  VIPETFNGEQLPR  VLEESPSVVISQTKR  AEAIHPGFGFLSENSR | Cyt | Cyt |
| PilT protein, N-terminal | EAN10184 | 2 | KLLDGVIVTNDYNLNK  KIEQETASQVESQLLER | Mem | Mem |
| Inorganic diphosphatase PpaC | ZP_06623379 | 2 | VEEAFQTTLTNNR  KEIAGIMLSAIISDTLLFK | Cyt | Cyt |
| Methionine--tRNA ligase | EAN08866 | 2 | AVPFGSDGVFTPEDFVSR  RFHTIYWPIMLMALDLPLPK | Cyt | Cyt |
| Elongation factor P | EAN08792 | 3 | SGATYDTTFRPEEK  SGATYDTTFRPEEKFEK | Cyt | Cyt |
| Cof-like hydrolase | EAN09356 | 2 | QFGSYVDVR  GVQEIANYLSIDQK | Cyt | Cyt-Mem |
| di-trans,poly-cis-decaprenylcistransferase | EEI60039 | 2 | RAEIVTAVQNIAEEVAK  ALWPDFDGAHLEEAIASYQNR | Cyt | Cyt |
| Adenylate kinase | AAL84190 | 3 | GELVPDEWINSLAK  LAEPDTEKGFLLDGFPR | Cyt | Cyt |
| Family 2 glycosyltransferase | EEI61366 | 2 | VNQLEKMVEILPVR  RIMAINSGVAWSVK | Cyt | Cyt |
| FeS assembly ATPase SufC | EEV55747 | 2 | MALLNMPEEMAER  TGEIHAIMGPNGTGK | Cyt | Mem |
| GTP-binding protein LepA | EAN09614 | 2 | ALIFDSIYDSYR  RDFLMVGDVGYITASIK | Cyt | Cyt |
| Vancomycin response regulator | AAA24787 | 2 | KSNNTITVHIR  QLSLTPTEFSILR | Cyt | Cyt |
| Orotidine 5'-phosphate decarboxylase | EAN09253 | 2 | IGMELFYQEGPEIVR | Cyt | Cyt |
| S-adenosylmethionine-dependent methyltransferase | EEI59359 | 2 | KVHNGFIQEELNDR  VLLDYGGGTGLVSLPLAER | Cyt | Cyt |
| UDP-glucose/GDP-mannose dehydrogenase | EAN09675 | 2 | NIIFSPEFLR  RIGTHYNNPSFGYGGYCLP | Cyt | Cyt |
| Ribosomal protein L35 | EAN08875 | 2 | ASMVSSGDFKR | Ext | Ext |
| PTS system fructose subfamily IIA component:PTS system sorbose subfamily IIB component | EAN08784 | 3 | IIVVSDAVSKDDLR  VDSRLLHGQVATAWTK  VLSMGQEDVEAFEKLEQK  VLSMGQEDVEAFEKLEQKGVK  VLSMGQEDVEAFEKLEQKGVKFDVR | Cyt | Cyt |
| ATP-binding protein | EFF61807 | 2 | GYLLEIQEPTVIR  YGLPIDSDIVMDVR | Cyt | Cyt |
| Putative LicD-family phosphotransferase | EFF19884 | 2 | KTDENYTLLAFETR  GFIPWDDDIDIVMPRPDYNK | Cyt | Cyt |
| N-acetylmuramidase | EEI59112 | 2 | RYEGLSFYSGGSKPIYR  NYLPTVGWEYEGIAWQAPNSGQPVYR | Ext | Ext |
| DeoxyguanosIne kinase | EFF32656 | 2 | KFDFVENPEDAK  SIYEDSLLFHLNADLGR | Cyt | Cyt |
| DNA polymerase III PolC | EAN09504 | 2 | YGIPEAANPVIDTLELAR  RAIAITDHGGAQAFPEAHSAGK | Cyt | Cyt |
| S-adenosyl-methyltransferase MraW | EFF32806 | 2 | IAVNDELGAEEASLEQAIR  KVDGILYDLGVSSPQLDEAER | Cyt | Cyt |
| Transcription termination factor NusA | EAN09506 | 2 | GPQVFVSR  TIIYNEFSAYEK  DALLINPAYEIGDTIR | Cyt | Cyt |
| MTA/SAH nucleosidase | EAN10591 | 2 | KEGLIVTGDTFVDSPDK  AMSDTADHSATQSFDEFIEDAGKR | Cyt | Cyt |
| Transketolase, central region:Transketolase, C terminal | EAN08636 | 3 | AILSLEAPIGR  QIVEDSLRVLNIQ  SFREEVPDEAYEVPLDK  SPFGGGVHTPELHSDNLEGLIAQSPGIR  YYGDSSMNVLNFGAEKEFTDSVPVGQLYER | Cyt | Cyt |
| branched-chain amino acid aminotransferase | EEI60435 | 2 | YSLLQLAEER  IEEVGAANFFGITK  GGLKPTNFIVSDYDR | Cyt | Cyt |
| 50S ribosomal protein L22 | EFD10707 | 2 | TSHITVVVSEK | Cyt | Mem |
| LexA repressor | EEI60453 | 2 | LQPENDLLEPIILDQVSILGR  RGDSMINAGILDGDNVIVR | Cyt | Cyt |
| SCP-like extracellular protein | EFF35540 | 3 | KISLDELR  AELQALYNR  GLQHKPETPK  ELAWLNDYR  KGDYTDNTWNAFQTALNNAK  GDFTEESWNNFQTALSNAKK  QQNGVAPMQFNDIVQQAADIR  WGNYEGPAWEAPTSGGHLVYR  KFNVTVNHVNADTNAVLSSESK | Ext | Ext |
| 30S ribosomal protein S7 | EEI61149 | 3 | AFAHYRW  RDVLPDPIYNSKLVTR  ESTGNDPLEVFEQAMK  VGGSNYQVPVEVRPER  VGGSNYQVPVEVRPERR  RVGGSNYQVPVEVRPERR | Cyt | Cyt-Mem |
| UDP-N-acetylmuramoyl-L-alanine--D-glutamate ligase | EFF62162 | 2 | TQYVGEIQGR  ETLHFFHGVPHR  TTTTTMTGLLLNAGADQGIAR | Cyt | Cyt-Mem |
| 1-deoxy-d-xylulose-5-phosphate synthase | EFF26929 | 3 | YVDNGNDLETVIR  EFTDSVPVGQLYER  LFEEVKDIDHPIVLHVHTEK  ETHELVITLEDGSLSGGFGENISR  IPTHGVENGTAIANDYSIPAYQTVNNGGK | Cyt | Cyt |
| GTP-binding protein TypA/BipA | EFF19770 | 3 | VEPTNSPDAWTVSGR  QSDTLDAHTQLQER  EGYELQVSRPEVIEK  VNIMDTPGHADFGGEVER  GTVFVEPGTEVYEGMIIGENSR | Cyt | Cyt |
| Penicillin-binding protein | EEV41311 | 2 | SGLEMAFDKDLR  AYEENPEQPFISR  SALSEKEILDKYQNIYGAADVK | Mem | Ext |
| Ribonucleoside-triphosphate reductase | EEI61284 | 2 | TVVTQQPEAHQMKVIKRDGR  LINKDQTVVNENANKDSNVFNTQR  ANADAWGDEYGYHFSVYSTPSESLTDR | Cyt | Cyt |
| Glutamyl-tRNA(Gln) amidotransferase A subunit | EAN09524 | 3 | AGQVRTLIKR  SLEELHHMLVSKEITVQDLTK  NAWDPTKVPGGSSGGSAAAVAAGQIPVSLGSDTGGSIR | Cyt-Mem | Cyt |
| 50S ribosomal protein L1 | EEI59184 | 3 | VSFDDEK  FDATVEVAYR  NVTITTTFGPGIHVDQASF  EAEAAGADFVGDDDMVQK  AGKVTYRVDKAGNIHVPIGK | Cyt | Cyt |
| Pheromone cAD1 lipoprotein | EFF23723 | 2 | NYSNGYR  SKQDDTEYNENMK  SGTNPEKYIPELNDQFLK | Ext | Ext |
| 30S ribosomal protein S10 | EEI61152 | 3 | TGASVSGPIPLPTER  ILDQSADKIVETAKR  RTGASVSGPIPLPTER | Cyt | Cyt |
| Glyceraldehyde-3-phosphate dehydrogenase, type I | EEI59791 | 3 | VGINGFGR  TVKVGINGFGR  HTVDNPSFGYDDR  LQGHAQRVPVVDGSLTELVSVLK | Cyt | Cyt |
| Tyrosyl-tRNA synthetase, class Ib | EAN10848 | 3 | DAINQQTNEER  TSPFEFYQFWLNQDDRDVVK  VLQTMEQVQHNVDALSNQMR  FFTFLSQEEIEDLAKKVETEPEKR | Cyt | Cyt |
| Elongation factor EF1A | EEI60673 | 2 | GLANPQDYASIDAAPEER  KGLANPQDYASIDAAPEER | Cyt | Cyt |
| Glutamyl-tRNA synthetase | EEV45513 | 3 | GAISFESDNIGGDFVIQKR  VAVSGQMHGPELPETIELLGR  VAVSGQMHGPELPETIELLGREK | Cyt | Cyt |
| Isoleucyl-tRNA synthetase | EAN10724 | 2 | FLLANTEDFDPKKDTVSYNDLR  LGVAGDWEHPYVTLDPSYEAAQVR | Cyt | Cyt |
| General stress protein Gls20 | EAN10936 | 3 | LVNTDNVTAGINTEVGKK  NMNTTNGSVLANEKGENR  MENKNMNTTNGSVLANEKGENR | Cyt | Cyt |
| DNA-dependent RNA polymerase subunit beta | AAO00728 | 2 | TGEPFDGR  QLGIHVATPVFDGASDEDVWETVR  VFSPRDPEREVNVIGNGYPEAAVK | Cyt | Cyt |
| Chaperone DnaK protein | EEI60127 | 3 | FQLTDIPAAPR  GFAEDYLGEKVEK  AVITVPAYFNDAQR  IVNEPTAAALAYGLDKTDRDEK  GFAEDYLGEKVEKAVITVPAYFNDAQR  SQVFSTAADNQPAVDIHVLQGERPMAADNK | Cyt | Cyt |
| Cobyric acid synthase, putative | EAN10272 | 2 | RNVFGSYFHGPILAR  VVQGQGNNGEDQSEGVIYR | Cyt | Cyt |
| 16S rRNA processing protein RimM | AFK59457 | 2 | EILSPGANDVWVVQRPK  VKEILSPGANDVWVVQRPK | Cyt | Cyt |
| Uridylate kinase | EEI60041 | 2 | QIAEPYIRR  AILGENIGTTVR  LSGEALAGDAGFGIKPPVIK | Cyt | Cyt |
| Phosphoribosylglycinamide synthetase | EAN09779 | 3 | FGDPETQVVLSR  EALDAAEQMLNQHR  LKSDFAQVIDDLLENR | Cyt | Cyt-Mem |
| Glutamine synthetase type I | EAN10445 | 3 | NIYVMDEEER  MMFDGSSIEGFVR  QTVSEWEREQYLELY  LVPGYEAPVYVAWSGR | Cyt | Cyt |
| ClpX, ATPase regulatory subunit | EAN09535 | 2 | MYDNPSSNETVR  ILEGTVASVPPQGGR  LLSLDNTELEFEPEALR | Cyt | Mem |
| HD/KH domain-containing protein | EAN10225 | 2 | RTSYGQNVLK  SLENISNSFAGVESSYAVQAGR | Cyt | Cyt |
| Asparagine--tRNA ligase | EEI59902 | 2 | NLHQETSVLVTGEIR  YGSVPHSGFGLGLER | Cyt | Cyt |
| Ribosome biogenesis GTPase YqeH | EFF31443 | 2 | AYEAGIRPVDVLLTSAK  VYQLNEGQTLFLGGLAR  HVGGLLQPPRPDEVEAFPELVR | Cyt | Cyt |
| Response regulator receiver:Transcriptional regulatory protein, C-terminal | EAN09345 | 2 | HEGYNAEVHYNGR  VSGLDHGADDYIVKPFAIEELLAR | Cyt | Cyt |
| Conserved hypothetical protein HMPREF0352_0336 | EEI61459 | 2 | QLVDRYPSNYHDVEVQIQK | Cyt | Mem |
| Peptidase S14, ClpP | EAN08823 | 3 | TGQPLEVIEKDTDRDNYMTAEQAK  FALPNAEIMIHQPLGGAQGQATEIEIAAR | Cyt | Cyt |
| GMP synthase, C-terminal:GMP synthase, N-terminal | EAN09438 | 3 | IVYDITSKPPATVEWE  ELGTQLGMPDSIVWR  AIGDQLTSIFVDHGLLR  SHHNVGGLPEDMQFELIEPLNTLFKDEVR | Cyt | Cyt |
| NADH oxidase | EEV50506 | 2 | QVTLIDGLDR  YLDKPFTDILEK  NHPEAEVTVYER | Cyt-Mem | Cyt |
| Ribosomal protein S5 | EEI61170 | 2 | ILMKPAVEGSGVAAGGPVR  NLVEVPMVGSTIPHEVIGAFSGGR | Cyt | Cyt |
| Conserved hypothetical protein | EEV42650 | 2 | RIMEVILSSTTAQK  ASQLGISPEEVASLSQQASLTR | Mem | Mem |
| LuxS protein | EAN08795 | 3 | RDEWSEVEA  TGAHGDVIIKYDVR  EFLAKRDEWSEVEA  VADRKTGAHGDVIIKYDVR  FKQPNKEHMDMPSLHSLEHLTAELIR | Cyt | Cyt |
| Phosphoribosylformylglycinamidine synthetase I | EEV55335 | 2 | EIMGADAEFVR  LPLLIMTTSTAAWK | Cyt | Cyt |
| Acetaldehyde dehydrogenase (acetylating) | EEI60950 | 2 | IGGEYGIPHGR  MLIAELEGVGPDYPLSR | Cyt | Cyt |
| Malonyl CoA-acyl carrier protein transacylase | EAN10060 | 2 | TIGIEQVIEVGPGK  LDQTQYTQPAILTVSIAYYR  MIPLNVSGPFHTAILEPAAK | Cyt-Mem | Mem |
| IMP dehydrogenase | EAN09302 | 3 | LSGLITIKDIEKVIEFPNAAK  YFQGSVNEANKLVPEGIEGR | Cyt | Cyt |
| Nicotinamide mononucleotide transporter PnuC | EAN10489 | 2 | MEEEYYNAQQHA  KNYLSIGEQIAYMITLDIPVLLSK | Mem | Mem |
| UDP-N-acetylglucosamine 1-carboxyvinyltransferase | EAN09183 | 2 | RQLEIEAPYEYVSQMR  HLNVDVDFDEQKNQVTIDASR | Cyt | Cyt |
| Chromosome partition protein SMC | EAN09848 | 2 | GGKMPDIIFAGSDTR  EQPKMYQLMNQVQQLR | Cyt | Cyt |
| Transcriptional antiterminator bglG:Sigma-54 factor, interaction region | EAN08782 | 2 | AVDMPLDMDPK  GLNCLITGPTGSGK | Cyt | Cyt |
| Cyclic nucleotide-binding:Bacterial regulatory protein, Crp | EAN08996 | 2 | VESEYAEFYR  RTEEGMLIDFSITNEEIAK | Cyt | Cyt |
| Coenzyme A disulfide reductase | EEV49679 | 2 | AGDIPENDLKEAFFLDIR  NNIDVFTKHEVTAIDPSTKR | Cyt | Cyt |
| Ribosomal protein L18 | EAN09418 | 2 | VQALAEAARENGLEF | Cyt | Cyt |
| Endonuclease/exonuclease/phosphatase | EAN08498 | 2 | TWRHLSDHAPLSAEIHL  RNAVYPEGHHGNAVLSR | Cyt | Ext |
| DNA-directed RNA polymerase., Acetylornithine deacetylase | EAN09721 | 2 | RTMITTVGK  RLGIQAFEPVLVQGR  TFHTGGVAGDDITQGLPR | Cyt | Cyt |
| AICARFT/IMPCHase bienzyme | EAN09780 | 3 | TLSIEEVTGFPEMMDGR  TLSIEEVTGFPEMMDGRVK ETIMKPDVEIAEAIENIDIGGPSMLR  HTAAYDALIAQYLTDWVEEKEPEKLTLTYER | Cyt | Cyt |
| DNA-directed RNA polymerase, beta subunit | EFF27850 | 3 | VNKFGFIETPYR  VLVKELQSLGLDMR  MNIGQVLELHLGMAAR  TYEAIVKGEPIPKPGVPESFR  ALGFGSDDTIFEIFGDSETLR  AQEVFFGDFPLMTEQGTFIINGAER  IMPEEDMPFLPDGTPIDIMLNPLGVPSR | Cyt | Cyt |
| Peptidoglycan-binding LysM | EAN10627 | 3 | YQLDASYLNGDYSAANQER  RYQLDASYLNGDYSAANQER  RIYVGEQLTIPTSNDSSATTENK | Ext | Ext |
| Beta-ketoacyl synthase | EAN10062 | 2 | RVSPMFVPMAIANMAAGNIALR NGFVMGEGAGVLVLESLEHAQAR | Cyt-Mem | Cyt |
| Ketose-bisphosphate aldolase, class-II:Fructose-1,6-bisphosphate aldolase, class II | EAN10443 | 2 | KGGYAVGGFNTNNLEWTQAILEAAEAK  GLAFDHLQAIADAVGPDMPLVLHGGSGIPQDQIEK | Cyt | Cyt |
| GTP-binding translation factor YchF | EAN09300 | 2 | GASKGEGLGNQFLSHIR AGAEAANYPFATIDPNVGMVEVPDWR  SLFLLTTKPILYVANVSEDDVADADANHYVQEVR | Cyt | Cyt |
| Glucokinase | EEI60064 | 2 | FAIDNDANVAALGER  WSIDTNILDDGKHIVPEIIESINHR | Cyt | Cyt |
| Cell envelope-related transcriptional attenuator | EAN08970 | 2 | VSIDDKEPFSVLLLGLDTGGLGR QDQLQGEGQMINDIYYQILGK | Ext | Ext |
| ABC superfamily ATP binding cassette transporter, ABC protein | EEI61182 | 2 | LQGFNIR  YFDAADQLYDGLK  DTTKEFDTDLTILDWLR | Cyt | Mem |
| Orotate phosphoribosyl transferase | EAN09254 | 2 | MVVIEDLISTGGSVLEAAEAAKR | Cyt-Mem | Cyt |
| GroES | AAN32668 | 2 | VIIEVAKEEEKTVGGIVLASAAK  YAGTEVKYEGKEYLIVAGKDIMAIVE | Cyt | Cyt |
| Ribosomal protein L24 | EFF30351 | 2 | HQKPSQAAPQGGIVEMEAPIHVSNVMVVDSTGVAGR  KHQKPSQAAPQGGIVEMEAPIHVSNVMVVDSTGVAGR | Mem-Cyt-Ext. | Cyt |
| Glutamyl-tRNA synthetase bacterial/mitochondrial | EAN10183 | 3 | GAISFESDNIGGDFVIQKR  VAVSGQMHGPELPETIELLGR  VAVSGQMHGPELPETIELLGREK | Cyt | Cyt |
| Serine hydroxymethyltransferase | EEV43046 | 2 | KINSAVFPGIQGGPLEHVIAGK  AVAFKEALDPAFKEYSEQIIANAK | Cyt | Cyt |
| Glycosyl transferase, family 8 | EAN10922 | 2 | KKMGEFGR  FSVKNARMNSDVEFLK | Cyt | Cyt |
| Phosphoribosylaminoimidazolecarboxamide formyltransferase/IMP cyclohydrolase | EEV47769 | 3 | TLSIEEVTGFPEMMDGR  TLSIEEVTGFPEMMDGRVK  NHEFVTSVVDPSDYEEVLSELR | Cyt | Cyt |
| Tripeptide aminopeptidase | EEI60258 | 2 | RKAQITHIQEELNQR  QAMIELGISPVIEPVRGGTDGSK | Cyt | Cyt |
| Cation-transporting ATPase Pma1 | EAN10220 | 2 | MIDPPKESAVKAVK  QAADMVLGDDNFHTIAKAVKEGRR | Cyt | Ext-Mem |
| Peptidase M20A, peptidase V | EAN09067 | 2 | ALIDAGVEFKTR  FVTEQHHEQALESLSELIR | Cyt | Cyt |
| Mannose-6-phosphate isomerase, type I | EAN10281 | 2 | ELHIQQSIDVTTVPAKTPELQIK  ELHIQQSIDVTTVPAKTPELQIKEVR | Cyt | Cyt |
| DNA polymerase III, beta chain | EAN09580 | 2 | VIPVEQAADHFDIVIPGK | Mem | Mem |
| Ribosomal protein S20 | EEI60551 | 2 | KFEDAVASGADNVDALYKEAVK | Cyt-Ext | Cyt-Ext |
| Tyrosine decarboxylase | EEI60793 | 2 | ALLNELVDEHLGWR KEIEVHPLTYPDFNMVDYVFK | Cyt | Cyt |
| Glycine hydroxymethyltransferase | EFF61030 | 2 | TYHFVAYGVDPTTEVIDYNVVR  KTYHFVAYGVDPTTEVIDYNVVR | Cyt | Cyt |
| Aspartate--tRNA ligase | EFF60874 | 2 | GIEVTLPFPR  QLLMNAGFDR  SEIVAAALGAIR | Cyt | Cyt |
| Group II intron reverse transcriptase/maturase | EFD08593 | 2 | TNLKLKVKDIAR  NPMNFSQDICNFTKQGRNK | Cyt | Cyt-Ext |
| Putative transposase | EFF27092 | 2 | RNAQLEEENLILK  RNAQLEEENLILKK | Cyt-Ext | Cyt-Ext |
| Transcriptional regulator NrdR | EAN09600 | 2 | IEAAPLLVIKK  RRECENCSFR | Cyt | Cyt |
| Low molecular weight phosphotyrosine protein phosphatase | EFF26581 | 2 | MGNICRSPMAEGLLR | Cyt | Cyt |
| Replication protein | ZP_06679557 | 2 | KDTDFMTDDEEK  KAMKLDYDPNVKVQMIRPK | Cyt | Ext |
| Cystathionine gamma-synthase/cystathionine beta-lyase | EAN08794 | 2 | NGLEYTIIDTSNLDKIEQSIKPNTK  LIHGGISEDPTTGAVSVPIYQTSTYRQDGVGQPK | Cyt | Cyt |
| beta-glucosidase | EEI59622 | 2 | LLDQAVRR  NAVGVMAAYNDIDGIPCHINR | Cyt | Cyt |
| Glutamate--tRNA ligase | EAN10183 | 2 | GAISFESDNIGGDFVIQKR  VAVSGQMHGPELPETIELLGREK | Cyt | Cyt |
| Sugar isomerase (SIS) | EAN10542 | 2 | NSKEIVKEIVAK  NSKEIVKEIVAKK | Cyt | Cyt |
| RNA binding S1 | EAN09060 | 3 | KVWEDIERDFQEGK  TEFDQNQENNQSMEDAMK  KVWEDIERDFQEGKVIEAPVTNVVK | Cyt | Cyt |
| Cell division protein DivIVA, putative | AFK58950 | 3 | LSNLEREVFGK  SAAVTNFDILKR  RLSNLEREVFGK  VQQTEAPKSAAVTNFDILKR  KLDQQASAVKPAQPNPNNYTNADTSLDDNEKTRQF | Cyt-Ext | Ext |
| DNA-directed RNA polymerase, beta' subunit | EFF27851 | 3 | LGIQAFEPVLVQGR  MQGVEIGDKHIEVMVR  TFHTGGVAGDDITQGLPR  IGVNPNSLGEKPFTEWQKER  MIDVNKFESMQIGLASPEKIR | Cyt | Cyt |
| DNA-directed RNA polymerase | EAN09428 | 3 | IDEEKDYGKFIVEPLER  IAKIDEEKDYGKFIVEPLER  AGINTVQELTNKSEPEMIKVR | Cyt | Cyt-Mem |
| Glutaminyl-tRNA synthase b subunit | EEI59851 | 3 | SSIQNEQIKEGHDLTEEEELTVLSR  DSLHEFEEAGRDDLAEKVKSEIVIVEK  MIKSSIQNEQIKEGHDLTEEEELTVLSR | Cyt | Cyt |
| Fructose-bisphosphate aldolase class-II | EEV42281 | 3 | SHIDWFGSANKA  QAPVLIQTSMGAAK  YIEAGKDKEGKGFDPR  AAIVEDAKSHIDWFGSANKA  LLAPGKAAIVEDAKSHIDWFGSANKA  GLAFDHLQAIADAVGPDMPLVLHGGSGIPQDQIEK | Cyt | Cyt |
| DNA gyrase, B subunit | EAN09583 | 2 | RAFIEENAHYVKNLDI  AKEYDASQIQVLEGLEAVR  YKGLGEMDDHQLWETTMDPSNR  NITYVQPGKNAEEELKQVVASLPASPKPSVQR | Cyt | Cyt-Mem |
| Protein of unknown function DUF964 | EFF27851 | 2 | SNIYDSANQIER  IIMTPVRDLYSE  SNIYDSANQIEREIR  SNIYDSANQIEREIRELPEFK | Cyt | Cyt |
| ATP-dependent Clp protease ATP-binding subunit ClpL | EFF33055 | 2 | AAVDYSIQYIPQR  YLVNGQSLTPDEFAQYR  YLVNGQSLTPDEFAQYRATGKLPQNNK | Cyt | Cyt-Mem |
| Phosphoglucosamine mutase | EAN09943 | 2 | ILVRPSGTEPLLR  VIEEAEAEMGSEGR  GKYFGTDGVRGEANKELTPELAFK | Cyt | Cyt |
| Initiation factor 3 | EAN08874 | 2 | LSPTIDLNDFNTKLR  VLNRLAEETADIATVEQK  VLNRLAEETADIATVEQKAK | Cyt | Cyt |
| Phosphocarrier HPr protein | EAN10050 | 2 | FNSDVNLEYKGK  EFHVVAETGIHARPATLLVQTASK  MEKKEFHVVAETGIHARPATLLVQTASK | Cyt | Cyt |
| Protein phosphatase 2C-like | EAN10247 | 2 | MQIEYQSDVGR  NTNQDYASVFTSR  SGEISEEMAVNHPR | Cyt | Cyt |
| Alanyl-tRNA synthetase | EEV41201 | 3 | YFDGSVVPENPR  FDFTHFGQITSDELAEMER  SPQLKEVVSKAEQLQQQVR  ALKEVLGEHANQAGSLVAPGHLR | Cyt | Cyt |
| Purine and other phosphorylases, family 1 | EAN10591 | 2 | QFNIPFLIVR  AMSDTADHSATQSFDEFIEDAGKR | Cyt | Cyt |
| Pyridoxal-dependent decarboxylase | EAN10847 | 2 | ALLNELVDEHLGWR  SSASFEHTVNKTKDVLSEISAR | Cyt | Cyt |
| DegV family protein | EEI60622 | 2 | TTGLLSSVLNIR  VVMNFAHSELIPVTK  VFNLAVAHGDAKEEAIEMEAR | Cyt | Cyt |
| General stress protein, putative | EAN08704 | 2 | VSESADEAGSESDDILLNLKEQSADLSNR  QKVSESADEAGSESDDILLNLKEQSADLSNR  STELTGIAKEKAQDLSQQAGDLAGSVK | Cyt | Ext |
| GTPase ObgE | EAN08613 | 2 | RDSDATWILSGEK  FKEQLNKEKEDEFADDIPVFPISGVTR | Cyt | Cyt |
| Alkaline shock protein | EEI61314 | 2 | GVYLANEEDGLK  GTFASNVTELLGR | Cyt | Cyt |
| CsbD-like | EAN10339 | 2 | NLGDEVKDKFSR | Ext | Ext-Mem |
| 3-deoxy-7-phosphoheptulonate synthase | EEI60186 | 2 | ECARMAKAGGAKILR  TSPYAFQGLEEEGLKYIR | Cyt | Cyt |
| OB-fold nucleic acid binding | EAN08481 | 2 | THNSEELHELFDPR  TQEQQHQVEDLNDQMLVRR | Cyt | Cyt |
| Beta-ketoacyl-acyl carrier protein synthase III (FabH) | EAN10058 | 2 | MMDTSDEWISSR  TTAVLFGDGAGGILLEANPQK | Mem | Ext |
| UvrB/UvrC protein:AAA ATPase, central region:Clp, N terminal | EAN09610 | 2 | LMMLDMGSLVAGTK  IQVDEPTPEEAEVILQGLR | Cyt | Cyt-Mem |
| ABC superfamily ATP binding cassette transporter, membrane protein | EEI61138 | 2 | IKGEPEWMLEFR  GGINAVPVGQMEASR  SVGLNPFASEYAGMSSK | Mem | Mem |
| Glucose 1-dehydrogenase | EEI60023 | 2 | TPINAAKFDDPEQKELLESMIPLGR  GNIINMSSVHEQIPWPTFAHYAASK | Cyt | Cyt |
| ArsC family transcriptional regulator | EEI59210 | 2 | AWLQEHQIPFVER | Cyt | Cyt |
| Heat shock protein HslU | EEV47837 | 2 | QIVSELDEYIVGQHAAK  QYVALIGTENVSVTFTK | Cyt | Cyt-Mem |
| Cell division transporter substrate-binding protein FtsY | EAN09850 | 2 | MNELFANFR  AGAIDQLVVWGER  GNAGGDPAAVVFDALER | Cyt | Cyt-Mem |
| Pyruvate dehydrogenase (lipoamide) | EAN08637 | 2 | YGPHTLSGDDPTRYR  LGFFAPTAGQEASQLASAYAFDK | Cyt | Cyt |
| Type I restriction-modification system M subunit | EAN10009 | 2 | GAELNQKLFSAADNLR  GEFYTPHMVSDMMAQIVTLDQK | Cyt | Mem |
| Peptide chain release factor RF3 | EAN08645 | 2 | WIDPEDLDEKMSSSR  RVNILDTPGHEDFSEDTYR | Cyt | Cyt |
| Translational initiation factor IF2 | EEV47331 | 2 | ADVQGSAEALAASLK  LDKGKGPVSTLLVQEGTLHVGDPIVVGNTYGR | Cyt | Cyt |
| 2,5-didehydrogluconate reductase | EAN08928 | 2 | WSLQHEFLPLPK  LWNANHSYELVMSSFEESLK | Cyt | Cyt |
| Ribonuclease PH/Ham1 protein | EAN09749 | 2 | LSAEWKQWLEGER  FGDTTVICSATIEDSVPPFLR | Cyt | Cyt |
| Bcl-2 family protein | EFF23123 | 2 | LAAVGDFVPESAR  LADIGSDHAYLPVALILR | Cyt | Cyt-Ext |
| Nucleotide-binding protein | EAN09108 | 2 | KLDLPIELQFTNYR  LENSNLVVLSDDDFK | Cyt | Cyt |
| Glutamate--ammonia ligase repressor | EEI61486 | 2 | QIFRDEILAQGGLSK | Cyt | Cyt |
| Carboxylesterase | EAN09195 | 2 | AVLLLHAYSGSPNDVR  SLAEQQLMDIQDQAAIVESR | Cyt | Cyt |
| DNA mismatch repair protein | EEV48697 | 2 | LALSHPSIAFR  ITVSNLFFNTPAR  ALSQENLIPSAADNLR  TIQVIDNGEGILADDVENAFKR | Cyt | Mem |
| Acetate kinase | EEI59236 | 2 | VLVIPTDEELMIAR  VLVIPTDEELMIARDVER | Cyt | Cyt |
| Extracellular solute-binding protein family 1 | EEV47188 | 2 | LTTMLSSGDTTDVLTMK  AAMMYMGTWYMAGILANK | Ext | Ext |
| Phosphopentomutase | EEV53559 | 2 | IIARPYVGEPGDFTR  DTMTGHWEIMGLNIK | Cyt | Cyt |
| Adenylosuccinate synthase | EAN09591 | 3 | IADLLDKEIFEER  IADLLDKEIFEERLR  QYVTDTSVILNDALDAGKR  VGDGPFPTELFDETGEQIR | Cyt | Cyt |
| PTS system protein | EEI60913 | 3 | AVILKPSEGPDDLR  IIVVSDAVSKDDLR  FSMESAHEIAAHIIETAKDGVK | Cyt | Cyt-Mem |
| Lysyl-tRNA synthetase | EEI59161 | 2 | ITYDGQAVDLESDFKR  EFANAFTELNDPIDQR | Cyt | Cyt |
| Polynucleotide phosphorylase/polyadenylase | EEI60913 | 2 | DGLVHISQLANDR  KLDEIRPLSSEVSLLPR  ALAQVIPSEEEFPYTIR | Cyt | Cyt-Mem |
| Glucokinase ROK | EAN09555 | 2 | EFTFPQVR  FAIDNDANVAALGER | Cyt | Cyt |
| Triosephosphate isomerase | EAN08761 | 2 | EYFHETDEDINK  QTVEKLYGKEVSEAVR | Cyt | Cyt |
| RNA polymerase, omega subunit | EAN10242 | 2 | RAHELDASAQPTLDSFDSVK  SVGQALEEIDAGNVVNDPHPELKR | Cyt | Cyt |
| Glycyl-tRNA synthetase, beta subunit | EAN09380 | 2 | LEDAEFFYNEDKK  NDQGMLLPHFISAR  FLDENQLTYDTIETFSTPR | Cyt | Cyt |
| Acetyl-CoA carboxylase, alpha subunit | EAN10067 | 2 | VIPETFNGEQLPR  LFADDEAIVGGIAR | Cyt | Cyt |
| Peptidase M24A, methionine aminopeptidase, subfamily 1 | EAN10665 | 2 | ALYIGIEQAQVGNR  KALYIGIEQAQVGNR  IGDIGHAIQTYVEGENLAIVR | Cyt | Cyt |
| Peptidase M3B, oligoendopeptidase F | EAN09218 | 2 | AYVLNHYLDGFK  YYGPAVEEDPEIKFEWSR  NDQDTGNTDYQALYAR | Cyt | Cyt-Mem |

a Gene locus given by blast in the NCBI (http://www.ncbi.nlm.nih.gov/); b subcellular localization predicted by Cellov.2.5 (http://cello.life.nctu.edu.tw) and Gpos-mPLoc (<http://www.csbio.sjtu.edu.cn/bioinf/Gpos-multi>). Cyt, Cytoplasm, CW, cell wall. Ext, extracellular. Mem, membrane.
