# Supplementary material for: Identification of Peptidoglycan-Associated Proteins as Vaccine Candidates for Enterococcal Infections
Source: PLoS One. 2014 Nov 4;9(11):e111880. doi: 10.1371/journal.pone.0111880 (PMC4219796; doi:10.1371/journal.pone.0111880)
Supplement: Table S2 — Summary of all the proteins identified by elution at high pH. (DOCX) [file pone.0111880.s002.docx]

**Table S2.** Summary of all the proteins identified by elution at high pH.

| Protein name | Gene Locus^a^ | No of replicates | Peptides identify by MS analyses | Subcellular localization ^b^ | |
| --- | --- | --- | --- | --- | --- |
|  |  |  |  | CELLO v.2.5 | Gpos-mPLoc |
| Ribosomal protein L7/L12 | EEV43331 | 3 | AVVDGAPAPVK  ALNIENIVAELK  EATILELNDLVK  AVVDGAPAPVKEGVSKEEAEELK  AVVDGAPAPVKEGVSKEEAEELKAK | Cyt | Cyt |
| Ribosomal protein S8 | EAN09416 | 3 | VITNLKR  RISKPGLR  DIAEILKR  VMTDPIADFLTR  DVEYIEDDKQGVIR  EGFVRDVEYIEDDKQGVIR  VLNGLGIAIISTSEGVVTDKEAR | Cyt | Cyt |
| Penicillin-binding protein | EEV41311 | 3 | FATGYAPGSTFK  SGLEMAFDKDLR  AYEENPEQPFISR  NVIGETALQTIVPDLR  TALIYSDNIYAAQETLK  IIDGATPELPAGATIQEVDGR | Ext | Ext |
| Ribosomal protein L19 | EAN10503 | 3 | TFPLHTPR  IQLFEGVVIK  GAGISETYTVR  MNPLIEELTK  IQLFEGVVIKR  RGAGISETYTVR  SDIPAFRPGDTVR  MNPLIEELTKEQLR | Cyt | Cyt |
| Ribosomal protein S9 | EAN10220 | 3 | AQVQYIGTGR  ALLEVDPDFR  DVEEYIPHADLR  EVINQPFAVTETK  KDVEEYIPHADLR | Cyt | Mem-Cyt-Extracell. |
| Ribosomal protein L21 | EAN08779 | 3 | QGHRQPYTK  VEVGQAIYVEK  VGAPTVAGATVEGTVEK  VVFDEVILVGGESTK  VEVGQAIYVEKLDVEAGEK | Cyt | Cyt |
| Glutaminyl-tRNA synthase b subunit | EEI59851 | 3 | YMPEQLSDEEIR  EGHDLTEEEELTVLSR  DSLHEFEEAGRDDLAEK  SSIQNEQIKEGHDLTEEEELTVLSR | Cyt | Cyt |
| Ribosomal protein S13 | EAN09426 | 3 | IAGVDIPR  ILADAGVSEDVR  ELTNEQTDAIR  VVVSLTYIYGIGNTTAK | Cyt | Cyt |
| 50S ribosomal protein L24 | EFF30351 | 2 | EGVVLAAFPK  NKEGVVLAAFPK  DKNKEGVVLAAFPK | Mem-Cyt-Ext | Cyt |
| Nucleoside 2-deoxyribosyltransferase | EAN09913 | 3 | TVTSFYSPR  ALEENKTVTSFYSPR  SQIYLAGPFFSEEQIDR | Cyt | Ext |
| Ribosomal protein L17 | EAN09429 | 3 | RQAAAFVR  LFNDIAPR  DLTTDLLINER  NEVASVREENEDIVIESALQK | Cyt | Mem |
| Rhodanese-like | EAN09554 | 3 | AGYQEIYLLK  SATTLTEEEFR  KAGYQEIYLLK  EKDVFDAGHILGAR | Mem | Mem |
| Glutamate ABC superfamily ATP binding cassette transporter, Membrane protein | EEI60115 | 3 | IILPQAIR  LQGFNIR  YFDAADQLYDGLK  GGINAVPVGQMEASR  VGTESAEFLSDHESEYGYSIK | Mem | Mem |
| ferritin | EEV43399 | 3 |  | Cyt | Cyt |
| Conserved hypothetical protein 250 | EAN10439 | 3 | AGYQEIYLLK  SATTLTEEEFR  KAGYQEIYLLK  EKDVFDAGHILGAR | Cyt | Cyt |
| Ribosomal protein S11 | EAN09427 | 2 | SLQATGLEVTAIR  STPFAAQMAAETATK | Cyt-Ext | Cyt |
| Ribosomal protein L11 | EEI59185 | 2 | TADQAGLIIPVVISVYEDR  DVYAFDLDEVAVR | Cyt | Cyt |
| ATP synthase subunit C | EEV42701 | 2 | QFLTEQR  SHFESETIR  MEYHELNPLIR  ELELLSNETFER  HAPIIVPLTIDEVR  GRELELLSNETFER  DAIHTQVSNELPESIMDSIR | Cyt | Cyt |
| Glutamate--ammonia ligase repressor | EEI61486 | 2 | AGLIIPVVIS  YYEEQDLVHPER | Cyt | Cyt |
| Ribosomal protein L15, bacterial form | EAN09421 | 2 | LGFEGGQTPLFR | Cyt | Cyt |
| Ribosomal protein L20, bacterial and organelle form | EAN08876 | 2 | EQVMNSYNYAYR | Cyt-Ext | Mem |
| Ribosomal protein S5, bacterial and organelle form | EAN09419 | 2 | SLGSNTPINVVR | Cyt | Cyt |
| Ribosomal protein S3 | EEV48626 | 3 | QLENRVAFR  EYAEFLHEDLR  AGAQGIKTQVSGR  SEGYSEGTVPLHTLR  ADIDYAWEEADTTYGK | Cyt | Cyt |
| 30S ribosomal protein S4 | EEI61012 | 2 | LDNVVYR  RPYAPGQHGPNSR | Cyt | Cyt |
| Ribosomal protein L5 | EAN09414 | 2 | LVSVSLPR | Cyt | Cyt |
| Ribosomal protein S12 | EEV41706 | 2 | PTINQLVR | Ext | Cyt |
| 50S ribosomal protein L4 | AFK57682 | 2 | GGGVVFGPTPR | Cyt-Mem | Cyt-Mem |
| Aminoglycoside phosphotransferase | EAN10527 | 3 | VLHFER  ELYDFLK  GTTYDVER  VSGFIDLGR  GTTYDVEREK  IIELYAECIR  YKGTTYDVER  LVGENENLYLK  EKDMMLWLEGK  ADKWYDIAFCVR  YKGTTYDVEREK  LFHSIDISDCPYTNSLDSR  TEKPEEELVFSHGDLGDSNIFVK | Cyt | Cyt |
| Ribosomal protein L2 | EEV41714 | 3 | APVSPWGQPALGYK  SANIALVHYEDGVK  KAPVSPWGQPALGYK  GSVMNPNDHPHGGGEGK  DNVVATVQTIEYDPNR  NMTGSDFAEITTSTPEK  ATIGSVGNEQHELINIGK  NKDNVVATVQTIEYDPNR | Cyt-Ext | Ext |
| Glutamate 5-kinase, ProB-related | EAN10187 | 3 | DVVDYPESR  NPAIIFDILAGK  EVGTLFKEDLK  FGDNDQLSAIMAK  FSVYNQQTAQILVTR  GKEVILVSSGAVGVGMHK  ILAQAGGAGSTFGTGGMQSK | Cyt-Mem | Cyt |
| Putative pyruvate, phosphate dikinase regulatory protein | EEV42906 | 3 | TPLSLFLANK  QALQDALEEK  VANLPLIPQAHIPK  QPGALHLLNENYFK  IVGLTNNPDILNNIR  SYGLNPDTAYSDIEK  KIVGLTNNPDILNNIR | Cyt | Cyt |
| Glucose-1-phosphate thymidylyltransferase, long form | EAN10659 | 3 | LSVELMGR  GIILAGGSGTR  VACLEEIAYR  EILIISTPQDTPR  DTGATVFGYHVNDPER  GIKPSERGELEITDVNK | Cyt | Cyt |
| Fructose-bisphosphate aldolase class-II | EEV42281 | 3 | PVVSGAEFLK  SHIDWFGSANK  SHIDWFGSANKA  QAPVLIQTSMGAAK  VNVNTEFQLSFAAATR | Cyt | Cyt |
| Hypothetical protein EfaeDRAFT_0546 | EAN08927 | 3 | SGENGIFER  LLNQFRPSLDGVR  INVPIDDNFNTVLK  INVPIDDNFNTVLKR  FYTEQNEHEVVINEK  LKNDEPTAQAVATPVTEETVSVPEEK | Cyt | Ext |
| Peptidylprolyl isomerase | EEI59596 | 3 | QSLAFQEGLK  QLGDSFDSQLK  QSLAFQEGLKK  EIDSQYDQTKK  SAGYTEQTFKDSIK  VKFDSTTTTVPAEVK | Mem | Ext |
| cell division initiation protein DivIVA | EAN10725 | 3 | DYEELTQR  ADELVANAEKR  HSEEKLEYFNELK  GYNQDEVDDFLDLVVR  DALNQSIIVAQDTADKVK | Cyt | Cyt |
| HAD-superfamily hydrolase | EEV43241 | 3 | QFGSYVDVR  IFASSRPGEK  FAGAVSAELTR  EIAFEILSQK  QLNLDFIAAENR  KQLNLDFIAAENR  AINAGHHVSIATGRPFR | Cyt | Cyt-Mem |
| Elongation factor Tu | EEI61151 | 3 | EHILLSR  TTVTGVEMFR  FSAEVYVLTK  ALEGDASYEEK  TTVTGVEMFRK  QVGVPYIVVFLNK  GQVLAKPGTITPHTK  VGDEVEVVGIAEETSK  FSAEVYVLTKEEGGR  LLDYAEAGDNIGALLR  HYAHVDCPGHADYVK  GITISTAHVEYETDTR  SKPHVNIGTIGHVDHGK  KLLDYAEAGDNIGALLR  NGGQAMAYDQIDGAPEER  ILELMAAVDEYIPTPER  HTPFFTNYRPQFYFR  ERGITISTAHVEYETDTR  VDMVDDEELLELVEMEVR  DNDKPFMMPVEDVFSITGR  NGGQAMAYDQIDGAPEERER  DLLTEYEFPGDDVPVVAGSALK  NMITGAAQMDGAILVVSAADGPMPQTR | Cyt | Cyt |
| NLPA lipoprotein | EEV45011 | 3 | IITILQDAGLVK  DNSPYVNIIATR  DGATIITSNSESDWGR  VKDGVDLETATFDDIEENPK  DVSELKDGATIITSNSESDWGR | Cyt | Cyt |
| Putative Soj | AAO52857 | 3 | AFTEEYLK  EFTQVGSEHR  EYTNDKLEVR  GIDVLPADIALSSAER | Cyt-Mem | Cyt-Mem |
| Hydrolase | EEV45310 | 3 | ALDVQEAAANVPIER  LLVETDAPYLAPVPFR  NLPTEEVAAQTWTNAHR  EALEDTYQILKEEDVR | Cyt | Cyt |
| Chain A, D-Alanyl-D-Lacate Ligase | EAN08995 | 3 | LQYGIFR  VDMFLQDNGR  YEPLYIGITK  NAGIATPAFWVINK  KVNSADELDYAIESAR  GSENAVITVPADLSAEER  IVLNEVNTLPGFTSYSR  DDRPVAATFTYPVFVKPAR  IHQEVEPEKGSENAVITVPADLSAEER | Mem | Mem |
| Pseudouridine synthase, Rsu | EAN09070 | 3 | AEEFILAGR  KAEEFILAGR  RKAEEFILAGR  YGDLTLQGLRPGDYR  LDYDTSGILLLTNDGDFAQR  LQIDQVDQETETSIIR | Cyt | Cyt |
| Aldose 1-epimerase | EEV41014 | 3 | GLNAYAIR  HAPVLFPFVGR  TYQMGQHGFAR  VGINQLGAHEIFK  ELFEQDALVFETR | Cyt | Cyt-Ext |
| LacX protein | EFF27494 | 3 | GLNAYAIR  HAPVLFPFVGR  TYQMGQHGFAR  VGINQLGAHEIFK  EIFEKDALVFETR | Cyt | Cyt-Mem |
| Transcriptional regulator | EEV42148 | 3 | QADLLYER  YFYFIQR  ELNIDLFQR  EANSGVFHYTQR | Cyt | Cyt |
| DNA-entry nuclease | EEW64988 | 3 | LSDGQWLYNR  KLSDGQWLYNR  REPLYIDPTGWK  VTPHFEGDELVAR | Ext | Ext |
| DNA polymerase, beta-like region | EAN10531 | 2 | YYIAGHVFR  LLETFEHKPEK  ANSGTDDKYYIAGHVFR | Cyt | Ext |
| SCP-like Extacellular protein | EFF35540 | 3 | ISLDELR  EVQWLTK  NNKPIYR  KISLDELR  AELQALYNR  GLQHKPETPK  ELAWLNDYR  SALEALYTSVK  NMTDQNYFLCR  QKELAWLNDYR  DHHYTASWDEVK  NYGWTYEGPAWR  IGVDNVFISANPIFK  YEGIGWYGADTSKPK  GLRDHHYTASWDEVK  GDFTEESWNNFQTALSNAK  GDFTEESWNNFQTALSNAKK  KGDYTDNTWNAFQTALNNAK  QQNGVAPMQFNDIVQQAADIR  WGNYEGPAWEAPTSGGHLVYR  KFNVTVNHVNADTNAVLSSESK  VEPTNSVTLNNVTESQNYIFNYTR  IKWGNYEGPAWEAPTSGGHLVYR  NSPAHNANLLYNNQSILGLGHNFVVDSAGR | Ext | Ext |
| Glycosyl transferase, family 2 | EAN08590 | 3 | QRPVYIIR  LASAYPYVR  KLASAYPYVR  TEYISYENR | Mem | Mem |
| Sporulation initiation inhibitor protein soj | EHM33068 | 2 | LYGDNLINTIVFR  KLYGDNLINTIVFR  GAADYIDYMEFFTER | Cyt-Mem | Cyt-Mem |
| Pur operon repressor | EAN10232 | 2 | YIESLAER  AIDDYTSLLYVK  ITEGSTVSVNYVSGSSER | Cyt | Cyt |
| 6-phosphofructokinase | EAN09351 | 3 | GGTFLYSAR  VSILGHVVR  TFVIEVMGR  LSEFGDYHTR  YPEFATEEGQLK  FGAYAVELLQEGK  RLDVADVGDKIQR  IGILTSGGDAPGMNAAVR  HKPDLSLYDLNNSLSF | Cyt | Cyt |
| Acetyl-CoA carboxylase carboxyl transferase, beta subunit | EAN10066 | 3 | ESLPVVLFTASGGAR  AEFLLSHGFVDQIVPR  VIEQTIKQELPEDFQK  RVIEQTIKQELPEDFQK  ESLPVVLFTASGGAR  AEFLLSHGFVDQIVPR | Mem | Cyt |
| Hemolysin A | EAN10744 | 2 | QGLFETR  KLPYVSR  EEGEVIALIKPQFEAGR | Cyt | Cyt |
| Heat shock protein Hsp33 | EEI59163 | 2 | ALAHDGFVR  AYAVQATNTVAEAQR | Cyt | Cyt |
| Rod shape-determining protein MreC, subtype | EAN10252 | 2 | TKPDSYGLDR  IELLSSSNESSNHFPVR | Ext | Ext |
| DegV family protein | EEI60622 | 3 | GLSGTVEAAR  TTGLLSSVLNIR  VVMNFAHSELIPVTK  VTVIDSDTTDQGLSFQVIR | Cyt | Cyt |
| Nucleotidyltransferase domain protein | EFF28841 | 3 | YYIAGHVFR  LLETFEYKPEK  ANAGAEDKYYIAGHVFR | Cyt | Ext |
| 2,5-didehydrogluconate reductase | EAN08928 | 3 | AFEELYEAGK  DNWEQANADTWR  ESGIPREEIFLTTK  HIDTAQGYKNEESVGQAIK | Cyt | Cyt |
| 16S rRNA methyltransferase | EAN09120 | 2 | ILAVFGPEGGLSR  DYFVLEGENYHHIVR | Cyt | Cyt |
| Elongation factor G | EEI61150 | 3 | VAASMALR  ILYYTGK  ELAEEWR  QATTYGVPR  EFSLQNTR  VMTDPFVGR  LSEEDPTFR  ELAEEWREK  TREFSLQNTR  VEANVGAPQVSYR  GITITSAATTAQWK  YLEGEEITEAELK  IGADFLYSVSTLHDR  AMVPLAEMFGYATTLR  GTFTMTFDHYEDVPK  SEISEVYAGDIAAAVGLK  EFKVEANVGAPQVSYR  VYSGVLQSGSYVQNATK  YLEGEEITEAELKEGIR  LYDGSYHDVDSNETAFR  INIIDTPGHVDFTVEVER  MDKIGADFLYSVSTLHDR  TDEEVERPADDSAPFSALAFK  IGETHEGASQMDWMEQEQER  VLDGAVTVLDAQSGVEPQTETVWR  AEMYTNDLGTEIEETEIPEEYR | Cyt | Cyt |
| 2-hydroxy-3-oxopropionate reductase | EAN10054 | 2 | HLLEAGHEVAVYNR | Cyt | Cyt |
| Pyruvate kinase | EAN09350 | 3 | FEVGDQTR  TTVQGTTEADFGR  AGYIKFEVGDQTR  FNFSHGDHEEQLSR  GATTTITEGELITVDSR  YRPDADILAVTFDER  FGLDNDIDFIAASFVR | Cyt | Cyt |
| Low affinity Penicillin-binding protein 5 | EFF35784 | 3 | ISGLEMAFDKDLR  AYEENPEQPFISR  VSLTTQEAAR  YQNIYGAADVK  FATGYAPGSTFK  TVEQFVQALNK  DSSWGSYQVTR  SGLEMAFDKDLR  AYEENPEQPFISR  NVIGETALQTIVPDLR  TVEQFVQALNKGDYNK  IIDGATPELPAGATIQEVDGR  SALSEKEILDKYQNIYGAADVK | Ext | Ext |
| Peptidase M24A, methionine aminopeptidase, subfamily 1 | EAN10665 | 3 | ALYIGIEQAQVGNR  KALYIGIEQAQVGNR  IGDIGHAIQTYVEGENLAIVR  DFIGHGVGPTIHEEPAVPHYGEAGK | Cyt | Cyt |
| Conserved hypothetical protein | EEV57022 | 2 | GFFQADNRR  LVSDYREGFNAEK  YDYIVGDWGYEQLR | Cyt | Cyt |
| RNA-binding S4 | EAN10726 | 2 | VLFGVLR  MDANVYQHFR  FMFYGGYEQAER | Cyt | Cyt |
| Putative LicD-family phosphotransferase | EFF19884 | 2 | VTHHPYDFYEK  TDENYTLLAFETR | Cyt | Cyt |
| Transcriptional regulator, MerR family | EFF21441 | 2 | FTAYYDER  LQHILFYR  FYDEIGLLKPAR | Cyt | Cyt |
| DegV | EAN09264 | 2 | GLSGTVEAAR  IDITSEEIYQR  DQVYTDKIDITSEEIYQR | Cyt | Cyt |
| Extracellular solute-binding protein, family 3 | EAN09753 | 2 | STNEIIWGVK  GNGLANEDILER  AVNQALEEMHADGTYDK | Ext | Ext |
| ABC transporter | EAN08750 | 3 | DLHLYYGK  SPFELSGGQMR  ETLEQVGLDESYLER  IFMNPEKQETEDYISGR  FEQVDFTYQPNTPFEQR | Cyt | Mem |
| Putative ABC transporter | CAD21830 | 2 | YQPDDLRPALK  GVDVPSEYMTEER | Cyt | Mem |
| ABC superfamily ATP binding cassette transporter, Membrane protein | EEI61138 | 3 | LQGFNIR  IILPQAIR  LPWLTQAFGGSR  GGINAVPVGQMEASR  SVGLNPFASEYAGMSSK  VGTESAEFLSDHESEYGYSIK | Mem | Mem |
| Formate acetyltransferase activating enzyme | EAN08938 | 2 | SDYDEYLIR | Cyt | Cyt |
| Sequence 6481 from patent US 6583275 | AAQ45923 | 2 | SFPDNVVIAGNPAR  SQSQIINQAESAELR | Cyt | Cyt |
| Transcription regulator | EEV56647 | 2 | LAGVHGFSDSLSTR  NNQPFHYSQSTVR | Cyt | Cyt |
| GntR family transcriptional regulator | EEI60711 | 2 | YEEIANILR  DGQPIEYSSSR | Cyt | Cyt-Mem |
| Hypothetical protein HMPREF9524_01757 | EFR68086 | 2 | VDAQIHDYPESTSLQR | Cyt | Mem |
| Cof protein:HAD-superfamily hydrolase, subfamily IIB | EAN10484 | 2 | GIAVAHVAHER  LELLHITYVDSIR | Cyt | Cyt-Mem |
| Conserved hypothetical protein | EEV42836 | 2 | WHNIQGR  AGGPDPSMNPALR | Cyt | Cyt |
| Haloacid dehalogenase-like hydrolase | EEV62227 | 2 | LNQPYQEIR  FQEQNEAFSTR  VLEHIVEFADENHR | Cyt | Cyt-Mem |
| Family 2 glycosyltransferase | EEI61366 | 2 | FYHEYDQR  LVDDYFEILR | Mem | Mem |
| DNA-(apurinic or apyrimidinic site) lyase | EEI60267 | 2 | AIKPLLLDQR | Cyt | Cyt |
| Thioredoxin reductase | EAN09045 | 2 | LGVPGEEEFAGR | Cyt | Cyt |
| VANA ligase | CAA40215 | 3 | SLTYIVAK  LQYGIFR  VDMFLQDNGR  YEPLYIGITK  SAIEIAANINKEK  EKYEPLYIGITK  NAGIATPAFWVINK  MMAAAGIALPELIDR  VNSADELDYAIESAR  KVNSADELDYAIESAR  VAILFGGCSEEHDVSVK  GSENAVITVPADLSAEER  IVLNEVNTLPGFTSYSR  DDRPVAATFTYPVFVKPAR  NHEYEINHVDVAFSALHGK  KNHEYEINHVDVAFSALHGK  IHQEVEPEKGSENAVITVPADLSAEER | Mem | Mem |
| Glycerol-3-phosphate dehydrogenase | EFF22517 | 3 | APSDLAIIGR  GLGHAVLQAR  AVIPAAGLGTR  KAVIPAAGLGTR  YDVGDKFGFMK  LVEETTDVNLHFIR  ASQVEEINTQHTNQR  TSIEYGLTHPEVGAPLR  THASTIAVMQVPHDETSK  YGIINPGEVLEDGLYNVK  NFVEKPDPSKAPSDLAIIGR  RPIEDHFDSNIELETNLSEK  AKRPIEDHFDSNIELETNLSEK | Cyt | Cyt |
| Vancomycin resistance protein | AAA24789 | 3 | QGAFLINTGR  GPLVDTYELVK  TIKNCLDFER  SEISASILLALK  MKQGAFLINTGR  SAPFNQCISVGHK  SEISASILLALKR  FGVMATIINANVSESNAK  MPNVIITPHTAYYTEQALR  MPNVIITPHTAYYTEQALRDTVEK | Cyt | Cyt |
| Elongation factor Ts | EEI60042 | 3 | FEVGEGIEKR  DVAMHVAAINPR  EDNFVEEVMSQVK  DDNAAFGGYLHMGGR  EDNFVEEVMSQVKK  GTIESDLIEATQVIGEK  YVNESQIPEAELEHEK  TVLTEQALNEGKPANIVEK  EIAELVAENKPADMEAAMK  TDKGTIESDLIEATQVIGEK | Cyt | Cyt |
| L-lactate dehydrogenase | EAN10259 | 3 | VIGSGTSLDSAR  QALAELVDVDAR  GATFYGIAVALAR  KGATFYGIAVALAR  VIEIPLSDSEQDR  TEGDAIDLSHALAFTSPK  NNPEIDEEAMVNLFFGVR  KIYSATYADAHDADLVVITAGAPQKPGETR | Cyt | Cyt |
| Cystathionine gamma-synthase | EAN08794 | 3 | QYEYSR  EEAGIKDGLIR  ALYLETPSNPLLK  FALEELIADLEGGVR  VYYPGLPDHELHGVAK  YLGGHSDVVAGLVTSNHK  NAFVVADFLFSHPAVEK  LIHGGISEDPTTGAVSVPIYQTSTYR  NGLEYTIIDTSNLDKIEQSIKPNTK | Cyt | Cyt |
| RecA recombinase | CAF21832 | 3 | LMSQALR  TIAIFINQIR  IIEVYGPESSGK  SGAWYSYKEDR  VGVMFGNPEITPGGR  AEIDGEMGDSHVGLQAR  EKVGVMFGNPEITPGGR  IDQQISTIPSGSLALDVALGVGGYPR  AAYGIGEEVAVPEDEKGQEELPLVEE | Cyt | Cyt |
| PpiC-peptidyl-prolyl cis-trans isomerase | EFF34785 | 3 | NNLAFEAGLK  DTYKEYIR  LESSNQSLVQR  VIGEELKDANVK  SFHPEVEAQIIK  QSLAFQEGLK  SAGYTEQTFK  NNLAFEAGLK  LESSNQSLVQR  SFHPEVEAQIIK  QLGDSFDSQLK  QSLAFQEGLKK  VIGEVLKDANVK  EIDSQYDQTKK  FDSTTTTVPAEVK  SAGYTEQTFKDSIK  GGTISEQEVMDSLKK  VKFDSTTTTVPAEVK  SLGDTFESQLEAAGYTK  LKDGQVSDVITSTNASTYTTEYYVVK  GGTITVSDFYDEAKLESSNQSLVQR | Ext | CW-Extracelular |
| Cysteine synthase | EFF31579 | 3 | VVTIFPDAADR  FDNQHYSMPK  LEYLNPGGSIKDR  VVTIFPDAADRYLSK  EQGLLVGSSSGAAFAAALR  MIISTITEAIGSTPLYR  LAEEISNSYLPLQFENK  TTIIEPTAGNTGIGIALAALTYK | Cyt-Mem | Cyt |
| Glyceraldehyde-3-phosphate dehydrogenase | EEI59791 | 3 | AAAANIVPNSTGAAK  VPVPTGSLTELVTVLEK  FNGTVEVHEGSFNVNGK  AIGLVIPELNGKLDGAAQR  IQDVDGIEVVAINDLTDAK  TVAWYDNEMSYTAQLVR  RIQDVDGIEVVAINDLTDAK  VVISAPGGNDVPTIVYNTNHETLTGK  RVVISAPGGNDVPTIVYNTNHETLTGK | Cyt-Mem | Cyt-Mem |
| Malonyl CoA-acyl carrier protein transacylase | EAN10060 | 3 | FYESIHK  TIGIEQVIEVGPGK  RGAYMTEAAPAGSGK  TAFLFSGQGAQYQGMGK  ETIAGLLEQQVMQPVR  MIPLNVSGPFHTAILEPAAK  LDQTQYTQPAILTVSIAYYR  YGIVSPANYNTPQQIVIGGEEK | Cyt-Mem | Mem |
| Ribose-phosphate pyrophosphokinase | EAN10234 | 3 | SKHYFDPR  IFALNSNRPLAEK  ANVAEVMNIIGQVK  LVVTDSIYLPEDR  TINVVMPYYGYAR  IHENKPVSPLFETK  GIKGDDVVVVSPDHGGVTR  SSVTQFSDGEIQVNIEESIR | Cyt | Cyt |
| VanY | EEV43760 | 3 | YVGLPHSAIMK  NTTIHVPTNLR  VEFQNYDQNPK  GGVSHFIINSGYR  TELTGIQYELWHIR  TITEEQVYQGNLLLINSK  YEISGNNIDGVIVTVFPGSTHTNSR | CW-Ext | CW-Ext |
| Aminotransferase AlaT | EEV41045 | 3 | EFLATYR  AAFYIFPK  ELLEEIVEVAR  SNKLEGVSYDVR  GYIEGLNMLSSMR  QNNLIIYSDEIYDR  LNTGNPAPFGFEAPNEVIR  EYIYNAINDIPGLSAVKPK | Cyt | Cyt |
| Cell division protein FtsZ | EAN10730 | 3 | FAAEGIAR  EFDTFNR  TPMLEAFR  KTPMLEAFR  AAEESEQSLR  VIGVGGGGGNAVNR  GLGAGSQPEVGQK  EFDTFNREETK  GVEFITANTDVQALK  SHGDDELNTPPFFR  LKENVDTLLIISNNR  ELGALTVGVVTRPFTFEGPK  TVMENQGTALMGIGVASGEDR  QGVQGISDLITAPGYVNLDFADVK  TVMENQGTALMGIGVASGEDRVVEATKK | Cyt | Cyt |
| DNA-directed RNA polymerase | EAN09428 | 3 | LHDLGLGLR  FIVEPLER  MIEFEKPR  VNYQVENTR  GYGTTLGNSLR  RVNYQVENTR  GVREDVTQIILNIK  IDEEKDYGKFIVEPLER | Cyt | Cyt-Mem |
| ATP synthase | EEV45692 | 3 | GINLAYELR  NLSDQPSFDEVR  NLSDQPSFDEVRK  TATDNAANIIDELTVSYNR  EIVTHLTATQLNDIASENPR | Cyt | Cyt |
| dTDP-glucose 4,6-dehydratase | EEI61370 | 3 | ENLAGLPSDR  QVTNILSGIRPK  EELGWQPEFTNFR  LREELGWQPEFTNFR  FTAETPYNPSSPYSSTK  LTYAGNKENLAGLPSDR  ATISNCSNNYGPYQHIEK | Cyt | Cyt |
| Inorganic diphosphatase | EAN08937 | 3 | VEEAFQTTLTNNR  IANFETANPLYYR  IAQVNTVDLNEVMDR  EKVEEAFQTTLTNNR  ETEAVALGTPNEETQYALDHFQLSAPR | Cyt | Cyt |
| Glycerol-3-phosphate dehydrogenase (NAD(P)+) | EAN10334 | 3 | MENEFSLR  LFMNDYFR  DGKMENEFSLR  ISEVLAEEIPEEKR  NIIAIGAGAIHGLGFGDDAK | Cyt | Cyt |
| Ornithine carbamoyltransferase | EAN09541 | 3 | QFDQAENR  MFDGIEFR  MKESVFQGR  AIMAATLGNLFIPR  FGITEMEVTDEVFR | Cyt | Cyt |
| PTS system fructose subfamily IIA component:PTS system sorbose subfamily IIB component | EAN08784 | 3 | LIEQAAPPGVK  RLIEQAAPPGVK  LLHGQVATAWTK  VKPEELEPAEAPK  AVILKPSEGPDDLR  IIVVSDAVSKDDLR | Cyt | Cyt |
| Catabolite control protein A | EEI59881 | 3 | LDYRPNAVAR  QTITIYDVAR  TIILPYGIEEK  IAFVSGALIDPINGQNR  LTSITQPLYDLGAVSMR  VPEEFEIITSNNSLLTEVARPR | Cyt | Cyt |
| D-alanyl-D-alanine carboxypeptidase | EFF35669 | 3 | IAPEYEAFAK  ITYEPWHLR  WIADNAAQYGFIVR  AAEAAGFPLVMVSAYR  SVSSQQQVFEQNVQDVMSR | Mem | Mem |
| 3-dehydroquinate synthase | EAN09101 | 3 | IGLSPTGSTAALER  VVEEDVFDQGNR  KVVEEDVFDQGNR  SLEQAAELYDFLADNDFTR | Cyt | Cyt |
| Peptidase U32 | EAN09175 | 3 | QINFWAK  NVPEYLAFLK  RPLLQNYYNYTK  YGLFDMPFGQER  IPYIYDAETLVTSAR  ELDTGFFYIDPSAIK  FVGEVLSYDEATQIATIR  TAIHYGADAVYIGGNAYGLR  ELATGFYYNTPTENEQLFGER | Cyt | Cyt |
| GTP-binding protein, HSR1-related | EAN10069 | 3 | FDFISGNR  ASFIAYVSNDVNIHR  AYEAGIRPVDVLLTSAK  VYQLNEGQTLFLGGLAR | Cyt | Cyt |
| Dihydroorotate dehydrogenase 1, partial | EAN09059 | 2 | EGPEIFSR  LKPEIQIIGTGGIR  EGFGGIGGEYIKPTALANVR | Cyt | Cyt |
| Vancomycin histidine protein kinase | AAA24788 | 3 | LYQYSIR  LEQLIDEFFEITR  QAVIHAPEDLTVSGDPDKLAR  EIIVQHGGQIYAESNDNYTTFR | Cyt | Mem |
| Beta-ketoacyl-acyl carrier protein synthase III (FabH) | EAN10058 | 3 | LIDWSDR  DIFDFAVR  DKPVDYLLLHQANLR  YGNTSAASIPILLDEAVSSGK | Mem | Ext |
| NAD(+) synthase | EEI59345 | 3 | MITQYAVAGER  FGDGGADILPLFR  AALDFIQPDVSLR  AGAVLGTDHAAENITGFFTK | Cyt | Cyt |
| Branched-chain amino acid aminotransferase | EEI60435 | 3 | YSLLQLAEER  VNLFRPEQNSR  IEEVGAANFFGITK  GGLKPTNFIVSDYDR | Cyt | Cyt |
| S-adenosylmethionine:tRNA ribosyltransferase-isomerase | EEI60944 | 3 | STLVMLVSAFAGR  VVEAFSTNFHLPK  LQAVVKEELEHGGR  LEDPDRYQTVYAK | Cyt | Cyt |
| Peptidase M24 | EAN10751 | 3 | AFFITDFR  NVGPIFDEVANLVR  SLGASGVSFDTIVASGLR  QFVPGNVITDEPGIYLPGIGGVR | Cyt | Cyt |
| PrgP | ABB46233 | 2 | DVLLIDYDQQR  SLAFIKPLEELR  NTTSNIGSTYQITSFDR | Cyt | Cyt-Mem |
| dTDP-4-dehydrorhamnose reductase | EAN10655 | 2 | AVEEILEDYYIIR  NVEYEVDDQTNPLNEYGR | Cyt | Cyt |
| Glucokinase ROK | EAN09555 | 2 | EFTFPQVR  FAIDNDANVAALGER | Cyt | Cyt |
| pseudouridylate synthase | EEV41212 | 2 | SAVTHFTVLER  VLTNLFTDYSR  YIGYPLAGDPVYGPK | Cyt | Cyt |
| Uncharacterised P-loop ATPase protein UPF0042 | EAN08628 | 2 | IEFVSFGFK  YGLPIDSDIVMDVR  FLPNPHYIPELRPLTGK | Cyt | Cyt-Mem |
| Glutamyl-aminopeptidase | EEI60990 | 3 | VVPLGGWNPYVVSAQR  SVEVTDILFDAGFESR  IKELTELQGTSGFEQDVR  GGTDAGAAHTQNEGIPSTVIGVVGR | Cyt | Cyt |
| Methyltransferase | EAN10738 | 2 | ISVITFHSLEDR  KPILPSEEELTENNR  IAVNDELGAEEASLEQAIR | Cyt | Cyt |
| Glycine--tRNA ligase | EEI59843 | 2 | GAGTMSPYTFLR  LLGIDPLEHDIR  AIGPEPWNAAYVEPSR | Cyt | Cyt |
| 50S ribosomal protein L2 | EEI61156 | 2 | SANIALVHYEDGVK  NKDNVVATVQTIEYDPNR | Cyt-Mem | Ext |
| Cell division transporter substrate-binding protein FtsY | EAN09850 | 2 | MNELFANFR  AGAIDQLVVWGER  GNAGGDPAAVVFDALER | Cyt | Cyt-Mem |
| Periplasmic solute binding protein | EAN10229 | 3 | GTEPHDFEPS  ISGVSPDQEPTP  QVAISGVSPDQEPTPSR  LLIPAGTEPHDFEPSAK | Mem | Mem |
| Peptidase M22, glycoprotease | EAN09544 | 2 | FGGVVPEVASR  LPEVELVIPPLR  SAFINLVHNAQQR | Cyt | Cyt |
| UTP--glucose-1-phosphate uridylyltransferase | EEI59439 | 2 | APSNLAIIGR  AVIPAAGLGTR  KAVIPAAGLGTR | Cyt | Cyt |
| Peptide chain release factor 1 | EAN08645 | 3 | TYNFPQNR  IQQEAQSEYDANR  IQQEAQSEYDANRK  LTHLPTGIVVAMQDER | Cyt | Cyt |
| D-alanine:D-alanine ligase | AAG49141 | 2 | ITLLYGGR  AIVEQGIEAR | Mem | Mem |
| Pyruvate dehydrogenase (acetyl-transferring) | EEI60529 | 2 | AILSLEAPIGR  QAGVAAQVVSEISER  SFREEVPDEAYEVPLDK | Cyt | Cyt |
| Oligopeptide/dipeptide ABC transporter, ATP-binding protein, C-terminal | EAN09842 | 2 | QYPHELSGGMR  ALELLEQVGIPNPAR  SDNQLLEVQNLHTGFR | Cyt-Mem | Mem |
| GTP-binding translation factor YchF | EAN09300 | 2 | GEGLGNQFLSHIR  APQAAGIIHSDFER | Cyt | Cyt |
| Phosphate starvation-inducible protein PhoH | EEI59849 | 2 | GVIEIAPLAYMR  KIDFVNFEASDVVR | Cyt | Cyt-Mem |
| UDP-N-acetylglucosamine diphosphorylase | EEI59134 | 3 | HADVGPYAHLRPK  NASATILTAQAENPTGYGR  VSETKPDEVITIVGHGAEQVK  NSSIAAGSTITDNIPEYALAIAR | Cyt | Cyt |
| UDP-glucuronate 5'-epimerase | EFF25088 | 2 | LYNIPSTGLR  DFEFKPSTSLR | Ext | Cyt |
| ParB-like partition protein | EAN09298 | 2 | EKPYYIR  GYEIIAGER  SRPYIANYLR | Cyt | Cyt |
| Aspartate-semialdehyde dehydrogenase | EEV45096 | 2 | ALQEAFGIQR  VLEEYDFPVGR | Cyt | Cyt |
| 1-phosphofructokinase | EAN09530 | 2 | GFYQELIDIIR  NSVGAGDSMIAGFIGAYSR  MIYTVTLNPSIDYIVR | Cyt | Cyt-Mem |
| 3-deoxy-7-phosphoheptulonate synthase | EEI60186 | 2 | SPVIFIER  SLQEGAFTR  TSPYAFQGLEEEGLK | Cyt | Cyt |
| Ribonucleoside-diphosphate reductase | EAN09926 | 3 | SIYYVR  QVFHNFEK  IGGGVGITLSNLR  SEIPEGIYEWK  YQPATPSFLNAGR  TNDGSAYLETYEDR  LFEDSFSYSNQLGQR  TLSLGVIVPDKFYELTR  LQQESGYPYVINIDTANR  LAVAPNGSISYINDTSASIHPITR | Cyt | Cyt |
| Spermidine/putrescine import ATP-binding protein PotA | EFF21600 | 2 | FNESEEEFDAR  VGLAFEPEDIHVMR | Cyt | Mem |
| Diacylglycerol kinase | EEV41394 | 2 | FIFYLPR  TLSQYHVGLLPGGTVNNLAR | Cyt | Cyt |
| Pseudouridine synthase, RluD | EAN11002 | 2 | NPFVLPILGR  GHPIVGDPLYQNR | Cyt | Cyt |
| Aminoglycoside adenyltransferase | AAK62560 | 2 | LHYAYPEYDR  DIVPTDIDYHVR | Cyt | Cyt |
| Sua5/YciO/YrdC/YwlC family protein | EEV45280 | 2 | ITGIIDDGATR  AGVIASPEIADQVR  DTLPSVVTGGLSTAAFR | Cyt | Cyt |
| Aminodeoxychorismate lyase | EEI61390 | 2 | NIAQVFFNR  IAIPEGYDIDQIAER | CW-Ext | Ext |
| Aspartate carbamoyltransferase | EAN09247 | 2 | LGAQVFFSGPR  HAIIMHPAPVNR  QYFATNLFFENSTR | Cyt | Cyt |
| Primosomal DnaI, N-terminal | EAN09602 | 2 | DIQSASFENFER | Cyt | Cyt-Mem |
| Glycosyl transferase, family 51:Penicillin-binding protein, transpeptidase | EAN08682 | 3 | IYGFVGGR  QYAYEHYLAK  IVNATNEGSNTFQTVR  NYSQNQNNHAFDTER  FGVGGTDENYTDYWNTASAYAR | Ext | Mem |
| Peptidase S1, chymotrypsin | EAN09870 | 2 | INSDKVETVASFGDSSALK  VGEPAIAIGSPLGSEYANSVTSGIISSLNR | CW-Ext | Ext |
| Dihydrouridine synthase, DuS | EAN10212 | 2 | GFPGANDLR  LQLDLQDHYSEIIPR  GSGLILRPEVAAQLIEAAK | Cyt | Cyt |
| Lipoyltransferase and lipoate-protein ligase | EAN09741 | 2 | ISEEYYR  VTQPIIQALHELGVAGAELK | Cyt | Cyt |
| Penylalanyl-tRNA synthetase | EEV52628 | 2 | NFYQNDLR  ITAAQDLNELNQIR | Cyt | Cyt |
| Phosphate acetyltransferase | EAN10482 | 2 | IVFPEATDAR  LKAEELVEPILVGNEEEIR | Cyt | Cyt |
| GTP-binding protein Era | EEI59845 | 2 | VYLQDFGYR  SGFVAIVGRPNVGK | Cyt | Cyt |
| tRNA (5-methylaminomethyl-2-thiouridylate)-methyltransferase | EAN10581 | 3 | VFEYFLAEYR  GQHAGLMYYTIGQR  LLDDNRAEVVFDEPVR | Cyt | Cyt |
| Biotin--acetyl-CoA-carboxylase ligase | EAN08834 | 2 | FEHGPTGYR  AISVFPDGQASVTR | Mem | Cyt |
| Ribosomal RNA large subunit methyltransferase N | EEV43060 | 2 | VSNFTEMTNLSK  LAYVNLIPYNPVSEHDQYSR | Cyt | Cyt |
| PilT protein, N-terminal | EAN10184 | 2 | ASQVESQ  KIEQETASQVESQLLER | Mem | Mem |
| di-/tripeptide transporter | EFF29058 | 2 | TLDGVGTTPPNPLTTEER | Mem | Mem |
| Beta-lactamase | EAN10282 | 2 | IVPTEQLPSGEVLR  YLPEFENQTITLR | Cyt | Mem |
| Glycosyl transferase, group 2 family protein | EFF19883 | 2 | VIFNENNLFLR | Cyt | Cyt-Mem |
| Dimethyladenosine transferase | EEV43080 | 2 | SLGQNFLTEPNILR | Cyt | Cyt |
| Guanosine monophosphate reductase 2 | EAN09771 | 2 | YLPETFVIAGNVGTPEAVR | Cyt | Cyt |
| Carboxy-cis,cis-muconate cyclase | EAN10291 | 2 | ISTLPEEFDGENGGAAIR | Cyt | Cyt-Ext |
| Pantothenate kinase | EAN09437 | 2 | NLQEYILPTR | Cyt | Cyt |
| Translation elongation factor Tu:Small GTP-binding protein domain | EAN08775 | 3 | EHILLSR  YLIVFLNK  AVVTGVEMFR  AVVTGVEMFRK  TLDYGEAGDNVGVLLR  HYAHIDAPGHADYVK  SKPHVNIGTIGHVDHGK  KTLDYGEAGDNVGVLLR  GITINTAHVEYETEKR  ELLSEYGFPGDDTPVIK  GLANPQDYASIDAAPEER  KGLANPQDYASIDAAPEER  DTDKPLLLPVEDVFSITGR  GLANPQDYASIDAAPEERER | Cyt | Cyt |
| ATP synthase F1, beta subunit | EAN09186 | 3 | VGLFGGAGVGK  VIDLLAPYLK  YDDLPEEAFR  VATEVQHVLQR  TIAMESTDGLQR  FTQAGSEVSALLGR  VALTGLTIAEYFR  TREGNDLYYEMK  TAMVFGQMNEPPGAR  EILEGKYDDLPEEAFR  APAFDELSTSTEILETGIK  KAPAFDELSTSTEILETGIK  MPSAVGYQPTLATEMGQLQER  VFNVLGDTIDLETPFPEDAER | Cyt | Cyt |
| Enolase | EAN08760 | 3 | YNQLLR  SIITDVYAR  EAGYTAVVSHR  IEDQLGEVAEYK  GMVPSGASTGEYEAVELR  GNPTIEVEVYTESGAFGR  VNQIGTLTETFEAIEMAK  AVDNVNNIIAEAIIGYDVR  GVYVLADSGEGEKTTDEMIK  SGETEDSTISDIAVATNAGQIK  AAADYLEVPLYHYLGGFNTK  GMVPSGASTGEYEAVELRDGDK  AGYVPGKDVVLAMDAASSEFYDKEK | Cyt | Cyt |
| GTP-binding protein | EEV41165 | 3 | EAIPFPR  IAFLQLR  QHAIMQIR  VFSFGPTFR  DGTAFFQGVVVK  TVTWIAGIDHVR  ATDYDFLLEQIR  YGSVPHSGFGLGLER  ATYEFFNQHHFVK  NLHQETSVLVTGEIR  NHVGETVTIGAWVANKR | Cyt | Cyt |
| Asparaginyl-tRNA synthetase, class IIb | EAN09362 | 3 | EAIPFPR  IAFLQLR  QHAIMQIR  VFSFGPTFR  DGTAFFQGVVVK  TVTWIAGIDHVR  ATDYDFLLEQIR  YGSVPHSGFGLGLER  ATYEFFNQHHFVK  NLHQETSVLVTGEIR  NHVGETVTIGAWVANKR | Cyt | Cyt |
| S-adenosylmethionine synthetase | EAN08618 | 3 | NIVAAGLAR  YYINPTGR  QTAAYGHFGR  IIVDTYGGYAR  KIIVDTYGGYAR  SNELTYLRPDAK  FVIGGPQGDAGLTGR  EVIPAELLDDQTK  FVIGGPQGDAGLTGRK  SQVTVEYDDQGQPER  TDVDLPWEHTDKVDALK  EVIPAELLDDQTKYYINPTGR | Cyt | Cyt |
| ATP synthase F1, alpha subunit | EAN09188 | 3 | LDLASYR  ELSLLLR  AIDALVPIGR  HVLIIFDDLSK  IMEVPVGEALIGR  ARPVEAMAPGVMQR  EAYPGDVFYLHSR  DLPEEEALNSAIQEYK  VVNPLGQPIDGLGEIVTDK  ELEAFTQFGSDLDAATQAK  EQIENYQNVLSVEEIGTVTYVGDGIAR | Cyt | Cyt-Mem |
| UDP-N-acetylmuramate--alanine ligase | EAN08897 | 3 | YFFTQR  LYHFVGIK  VYLCDIFGSAR  AIFAYGDDAYLR  AIFAYGDDAYLRK  KAIFAYGDDAYLR  EIIAVFQPHTFTR  IVADMTVVDDYAHHPAEIK  ANVPIYYYGVTENDDIQAR  QKYPDKEIIAVFQPHTFTR | Cyt | Cyt-Mem |
| Helicase, C-terminal:DEAD/DEAH box helicase, N-terminal | EAN08953 | 3 | IISQVPDQR  VQAVYGGADIGR  DRPHIVVGTPGR  ITPERPLPMQK  AEGIHGDLSQQKR  ELAIQTQEELYR  SGHLDILVATDVAAR  EMTADLIDQYYVR  AKEMTADLIDQYYVR  IDPDRHELQGLVIAPTR  AGFEEATPIQAETIPLALAGK | Cyt-Mem | Mem |
| Cell division protein FtsA | EAN10731 | 3 | YLSEIISAR  IAVTGETSAAR  INYGDAYPER  LYVPNHMGLR  LEMYGLLFTGPK  FTNLDQEGGEFVTK  GIIVDIDKTVQAIQR  QIVSILPQDFTVDGFEGIKDPR  TSPDEEFPVDVIGQSEPVKVDER | Cyt | Cyt |
| GroEL | EAN09274 | 3 | ALEEPIR  IEDALNATR  NVTAGANPLGIR  SYGSPLITNDGVTIAK  NQIAETTSDFDREK  GIETELDVVEGMQFDR  TNDIAGDGTTTATVLTQAIVR  VVVDKDNTTIVEGSGEKEAIEAR | Cyt | Cyt |
| Mannosyl-glycoprotein endo-beta-N-acetylglucosamidase | EAN10197 | 3 | DATAWLTGR  YEGIGFYGY  DWLTPMGWR  HTGEHLYTLNAGEK  SNTNSYKDATAWLTGR  YEGLSFYSGGSKPIYR  TTSFQPGVYYYSGAWK | Ext | Ext |
| Glutamyl-tRNA(Gln) amidotransferase B subunit | EAN09523 | 3 | YFPEPDIPK  LTPENLAGMIR  FVIDDEWIQK  VLLSGGEIQQETR  SPEEAYAYLEALR  QGTPLIEIVSEADMR  CDANISLRPYGQEEFGTK | Cyt | Cyt |
| GTP-binding protein YchF | EAN09300 | 3 | QVDAICHVVR  GEGLGNQFLSHIR  APQAAGIIHSDFER  DYVVQDGDVMLFR  NFAAEENAEVIVVSAR  KTVPTTFEFTDIAGIVK  AETVSFEDLDHYGNMHAAK  AAYDLLGLATYFTAGEQEVR | Cyt | Cyt |
| Acetyl-CoA carboxylase, biotin carboxylase | EAN10065 | 3 | FGHVIHLGER  IQNLLLPAGGMGLR  VLEESPSVVISQTKR  INAENPAFHFAPSPGK  AEAIHPGFGFLSENSR | Cyt | Mem |
| GMP synthase, C-terminal:GMP synthase, N-terminal | EAN09438 | 3 | TYDYTVGIR  QPFPGPGLGIR  IVNEVAHVNR  HSEYGIELLR  DLGVFSELLSHR  AVTSIDGMTADFAR  IIVLDYGSQYNQLITR | Cyt | Cyt |
| ClpX, ATPase regulatory subunit | EAN09535 | 3 | FGLIPEFIGR  SLAVAVYNHYK  EIIDEEFYEEAVR  MYDNPSSNETVR  ILEGTVASVPPQGGR  LLSLDNTELEFEPEALR | Cyt | Mem |
| phosphoglycerate kinase | EEI59790 | 3 | AILFSHLGR  GEQLEEAIR  EVTFVPETR  AALPTINYVLEQGGK  VDFNVPLKDGVITDDTR  YWASLGDVFVNDAFGTAHR  AHASNVGIASTGIPTVAGFLMEK | Cyt | Cyt |
| Sodium-transporting two-sector ATPase | EAN10388 | 3 | LAEYYER  VIDTFFPVTK  MILTFGEEGRR  LNPDAPMITGQR  VFWGLDSSLAQK  TVLIANTSNMPVAAR  ILQEEQQLNEIVR  EASIYTGITIAEYFR  LEEMPGDEGYPAYLGSR  EDYLQQNAFDDVDTFTSR  GDVASIQVYEETSGIGPGEPVR  SIREDYLQQNAFDDVDTFTSR  EGSITAISAVSPSGGDISEPVTQNTLR | Cyt | Cyt-Mem |
| Gamma-glutamyl phosphate reductase GPR | EAN10188 | 3 | YIIYGDGQIRE  LTEYLDVLIPR  DSAQQLALMPTNR  VVDSLDEAIAHINR  HSETIVTDSYAASQR  FLNEVDAAAVYVNASTR | Cyt | Cyt |
| serine protease serine protease serine proteaseSerine protease | EEW64168 | 3 | AELQALYNR  SALEALYTSVK  NYGWTYEGPAWR  YEGIGWYGADTSKPK  GDFTEESWNNFQTALSNAK  KGDYTDNTWNAFQTALNNAK  VEPTNSVTLNNVTESQNYIFNYTR | Ext | Ext |
| carboxylate--amine ligase | EAN08468 | 3 | IFLEYAR  KIFLEYAR  EEFDLILGR  YMFHNYIPR  YGTTVFYEKDR  ICQAYASDQLAPTR  SSFFVTLNGLNLAR  VLNAYVDEDHQVR  SEMIVQDFIPGDDSNMR | Cyt | Mem |
| Toxic anion resistance | EAN10595 | 3 | AGEGNIFQR  QITIQQAPQIR  IQASIATAIPLWK  IDANDAQSVISYGSAAQAK  LQEANPDELRAGEGNIFQR  AEETGDQMDVQIANDYTQFLDR | Cyt | Cyt |
| Glutamyl-tRNA(Gln) amidotransferase A subunit | EAN09524 | 3 | SLDDVYVNSR  EYLGEGVAPGVK  QPAAFNGIVGMKPTYGR  ILYNFEPIYDATVMDK  TMYQAAYAFEQATEFHK  DGTSSGVSVPDFAEGLTGDIK  YGVAVYYIIASSEASSNLQR  VPGGSSGGSAAAVAAGQIPVSLGSDTGGSIR | Cyt-Mem | Cyt |
| MurC UDP-N-acetylmuramate-L-alanine ligase | EEV41827 | 3 | FFGLSIPEIR  QGLAHVSITQNR  NFPVIPVADPLK  YPQLPVFYFEK  AMQQLATYYLEK  ITEGSLFVPLAGTR  KITEGSLFVPLAGTR | Mem | Mem |
| Trigger factor | EEI60214 | 3 | SLNVPGFR  KSLNVPGFR  QFEGEAEMR  FTIAQDEIQK  AADDAKDEAAIR  SMDEFLNNMQR  QDREVTDADVDAR  EKAADDAKDEAAIR | Cyt | Cyt |
| Cytoplasmic peptidoglycan synthetase, N-terminal:Cytoplasmic peptidoglycan synthetases, C-terminal | EAN10987 | 3 | AEFLQLSYDSR  SHLTTPESLDLYR  ETADLFQIPTITYGR  TVVLNHESDYFDLLR  INGVDEPYEGDYAIVKR | Cyt-Mem | Cyt-Mem |
| 6-phosphogluconate dehydrogenase, decarboxylating | EAN09346 | 3 | GYSVALFNR  LPANIIQAQR  YISAYKEER  DYFGAHTYER  IMSYAQGFAQLR  ATYTIEEFVESIEKPR | Cyt | Cyt |
| Arginine deiminase | EAN09542 | 3 | NYVSNELLR  ESMFTEYILK  VLEINSSELSR  MLDESHIASNAVR  RPGQEVENLTPDIMDR  AALGLDDLVLIPTGNGDEIVAPR | Cyt | Cyt |
| M20 family peptidase PepV | EEI60987 | 3 | VTPDAPFGPGPR  ATAIYADAIYR  GYEQIIGGGTYGR  TIDWQKEVEAR  GVTVDGLQSDIEQTVGQEGATVTR | Cyt | Cyt |
| IMP dehydrogenase | EAN09302 | 3 | ALYDAGVDVVK  ISGVPIVETLENR  VIEFPNAAKDEHGR  SGMGYVGAANLQQLR  VVAGVGVPQLTAIYDAASVAR | Cyt | Cyt |
| Amino acid adenylation:D-alanine-activating enzyme | EAN09734 | 3 | IPVGFYPR  KIPVGFYPR  QLFATLPALK  LDEYGLLFYEGR  QASDALAYYLEK  TAESLLDRFPNAR  IELGDIEHYLLQDNR  VAYQADETHTFGELK | Cyt | Cyt |
| GTP-binding protein EngA | EEI60700 | 3 | VAGYAHEAGR  AFTFEGTSIR  FLMIDTAGMR  ISIVEDTPGVTR  SSLINAILGEER  DFEQEIRDEFR  ANPTIAIVGRPNVGK | Cyt | Cyt |
| FMN-binding | EAN09170 | 3 | YIPELNDQFLK  TYIPTLNDEFVK  VTESNFDYIDKDGK  VVAGGDLQDGTYKLEEK  ITESKYDNVNENGESK | Ext | Ext |
| Glucose-6-phosphate isomerase | EAN094716 | 3 | SGTTTEPAIAFR  GWIDLPTNYDKEEFAR  VEADAEGWETFVIPDDIGGR  VAPFVNEQELGYMQSQVTAAHNELR | Cyt | Cyt |
| Basic Membrane lipoprotein | EFF34523 | 3 | VWVIGVDR  GEVIDRFDAGFK  SFNQSAWEGLEK  VLNQYAGDFSAPDK  VLNQYAGDFSAPDKGR  SADGKFPGGEHTVYGLK  SSSDTTTAALITDTGGVDDR  DNEASYLAGVAAAYTTETNVVGFIGGVK | Ext | Mem |
| Chromosomal replication initiator protein, DnaA | EAN09579 | 3 | QIAMYLAR  YTFDTFVIGK  TAEEFRQEYR  QIVLTSDRLPNEIPK  IVEVGYMLSGNEIIPR  DLSPASYNTWIETANPR | Cyt | Mem |
| Putative ATP-dependent RNA helicase DbpA | EFF62078 | 3 | GFKQPTEVQER  LHHQQPQVVVGTPGR  NQGLKVAKIHGDITPRER  QVQNLEYQYVVATDLAAR | Cyt | Cyt-Mem |
| S-adenosyl-L-methionine-dependent methyltransferase | EEI60011 | 3 | ELLIQAFR  NVSFEQLALYR  EIYPEIIGGYEK  TFFFSDDSTTAFR  IRFESVNLPESQFIYGK | Cyt | Cyt |
| GTP-binding protein YqeH | EEV54363 | 3 | FDFISGNR  AYEAGIRPVDVLLTSAK  VYQLNEGQTLFLGGLAR  HYNEIQDVSLTDDDFLR | Cyt | Cyt |
| Tyrosyl-tRNA synthetase, class Ib | EAN10848 | 3 | ISLLEFLR  ITQALFSGNIK  MNIIDELTWR  DAINQQTNEER  ELNAEEIAQGFGK  FVHSEEDLKEAQK | Cyt | Cyt |
| Glutamyl-tRNA synthetase bacterial/mitochondrial | EAN10183 | 2 | TALFNYLFAR  YAPSPTGHLHIGNAR  EIYQPLIDQLLASNR | Cyt | Cyt |
| Ribonuclease PH:Ham1-like protein | EAN09749 | 3 | QWLEGER  LLYELTDVPDER  KDSLVVEAEWPGR  TLLDYPELPDVEETGSTFEENAR | Cyt | Cyt |
| Transcription termination factor NusA | EAN09506 | 2 | GPQVFVSR  TIIYNEFSAYEK  DALLINPAYEIGDTIR | Cyt | Cyt |
| Adenylosuccinate synthetase | EAN09591 | 2 | IADLLDKEIFEER  VGDGPFPTELFDETGEQIR | Cyt | Cyt |
| Glucose-6-phosphate dehydrogenase | EAN10723 | 3 | FTIDNFR  WSGVPFYVR  EVFPEQDIFR  IYNEEEVLENFVR | Cyt | Cyt |
| TPR repeat | EAN08530 | 2 | EYAFFLR  QRAEELLLK  ENQQAINYLEELR | Cyt | Cyt-Mem |
| DAK2 domain protein | EFF21282 | 2 | GNSGVILSQLFR  IGEGPTVDSTFDYEEFR | Cyt | Ext |
| Nicotinate phosphoribosyltransferase related | EAN10309 | 3 | ELGDKINFQGVR  ADLHAVFECYFR  SVIGEDPLLEFGTR  VREQLDAAGFTEAK | Cyt | Cyt |
| DnaJ central region:Heat shock protein DnaJ, N-terminal:Chaperone DnaJ, C-terminal | EAN08838 | 3 | IPAGTQTGTNFR  LKIPAGTQTGTNFR  VNVPAGVEEGQQMR  EVSEAYEILSDPQKR | Cyt | Cyt |
| 30S ribosomal protein S1 | EEI60699 | 3 | VLSINPEQER  IIEIEPSENR  VLEIHPEEHR  EILSTIHEGDVLSGR | Cyt | Cyt |
| NADH oxidase | EEV50506 | 2 | QVTLIDGLDR  YLDKPFTDILEK  NHPEAEVTVYER | Cyt-Mem | Cyt |
| UDP-N-acetylmuramoylalanine-D-glutamate ligase | EEW66919 | 3 | LYGISNEAIR  TQYVGEIQGR  ETLHFFHGVPHR  TTTTTMTGLLLNAGADQGIAR | Cyt | Cyt-Mem |
| UDP-glucose 6-dehydrogenase | EFF25089 | 2 | LFANTYLALR  ALYDNLYPSR  NILFSPEFLR | Ext | Cyt |
| DnaB helicase | EAN09590 | 2 | VPVIALSQLSR  ELKVPVIALSQLSR  GFEQGEEVQTILDDAER | Cyt | Cyt-Mem |
| Aminotransferase, class I and II | EAN09361 | 2 | VTVEQLEQAR  NDILIVADDIYGR  ASYYTPTAGIPELR | Cyt | Cyt |
| UDP-glucose/GDP-mannose dehydrogenase | EAN09675 | 2 | LFANTYLALR  ALYDNLYPSR  NIIFSPEFLR | Cyt | Cyt |
| Beta-ketoacyl synthase | EAN10062 | 2 | LFANTYLALR  ALYDNLYPSR  NIIFSPEFLR | Cyt-Mem | Cyt |
| Signal recognition particle protein | EAN08916 | 2 | LTDLEVFHPDR | Cyt | Mem |
| Putative ATPase | BAB68231 | 2 | YPHELSGGQQQR  VIQFDTPENILR | Cyt | Mem |
| Queuine/other tRNA-ribosyltransferase:Queuine tRNA-ribosyltransferase | EAN08608 | 2 | QAIMDDNLLEFR  VLEFTTPLIPENKPR | Cyt | Cyt |
| Peptidase M20A, peptidase V | EAN09067 | 2 | DITGDMTEPFVSGGATFAR | Cyt | Cyt |
| Cell envelope-related transcriptional attenuator | EAN08970 | 2 | VLSLDGVSNYR  VSIDDKEPFSVLLLGLDTGGLGR | Ext | Ext |
| Mur ligase | EAN09591 | 2 | SPNATIISNGDSPIFNSIETVNPR | Cyt | Cyt |
| Hydroxymethylglutaryl-CoA synthase | EFF31663 | 2 | ALQSVLDQTDEENQER | Cyt | Cyt |
| Glutamine synthetase type I | EAN10445 | 2 | QTVSEWEREQYLELY  LVPGYEAPVYVAWSGR | Cyt | Cyt |
| tRNA (uracil-5-)-methyltransferase Gid | EAN09574 | 2 | RPYAVIQLR  MIPGLENAEFVR | Cyt | Cyt |
| histidyl-tRNA synthetase | EAN09125 | 2 |  | Cyt | Cyt |
| HI0933-like protein | EAN09473 | 2 | TEFEEFHAPAIILTTGGR | Cyt-Mem | Cyt |
| YvlB family protein | EEI59891 | 2 | MGAEPFEAFSER  SQIEVNEDHISFQIPNKR | Cyt | Cyt |
| Dihydroorotase multifunctional complex type | EAN09248 | 2 | VLQYAPITENLR | Cyt | Cyt |
| coproporphyrinogen III oxidase | EEV57297 | 2 | TIESVYGDVLR  GTFLGNNVFER | Cyt | Cyt |
| ATP-dependent Zn protease | EEV50395 | 2 | GYGLLINSVVITR  YIEEQSAYILER | Cyt | Cyt-Mem |
| Thiamine biosynthesis protein | EAN09799 | 2 | IFGIQNFSPSIR | Cyt | Cyt |
| Acetate kinase | EEI59236 | 2 | LAYDIFVDR | Cyt | Cyt |
| YycH | EAN09267 | 2 | IFVEGFPVFGTDSR | Ext | CW-Ext |
| Phosphoglucosamine mutase | EAN09943 | 2 | ILVRPSGTEPLLR | Cyt | Cyt |
| 3-phosphoshikimate 1-carboxyvinyltransferase | EAN09098 | 2 | GSHSFVQPTESIDMGNSGTTIR | Cyt-Mem | Cyt |
| 32 glycosylhydrolase | EEV43666 | 2 | LYQYPVEAITSLR | Cyt | Ext |
| Conserved hypothetical protein, putative | EFF35540 | 3 | ISLDELR  EVQWLTK  NNKPIYR  KISLDELR  AELQALYNR  GLQHKPETPK  ELAWLNDYR  SALEALYTSVK  NMTDQNYFLCR  QKELAWLNDYR  DHHYTASWDEVK  NYGWTYEGPAWR  TVYEGDSFATPWK  IGVDNVFISANPIFK  YEGIGWYGADTSKPK  GLRDHHYTASWDEVK  GDFTEESWNNFQTALSNAK  GDFTEESWNNFQTALSNAKK  QQNGVAPMQFNDIVQQAADIR  WGNYEGPAWEAPTSGGHLVYR  IKWGNYEGPAWEAPTSGGHLVYR  VEPTNSVTLNNVTESQNYIFNYTR  EQIVNVTHGDSVTLTAPSIQGYVLDDR  NSPAHNANLLYNNQSILGLGHNFVVDSAGR | Ext | Ext |
| Small GTP-binding protein domain:GTP-binding protein TypA | EAN09390 | 3 | LTFLTPAR  LFGFFGLK  LFGFFGLKR  ATTYSIMSIEER  GELHLSILIENMR  ALEQHITPIVVVNK  GLIGYSTEFLSMTR  VEPTNSPDAWTVSGR  QSDTLDAHTQLQER  EGYELQVSRPEVIEK  REGYELQVSRPEVIEK  VNIMDTPGHADFGGEVER  MVDGVVLVVDAYEGTMPQTR  VQIDTPEEYMGSVIESLSLR  GTVFVEPGTEVYEGMIIGENSR | Cyt | Cyt |
| Setrine protease | EEV54283 | 3 | EVQWLTK  NNKPIYR  AELQALYNR  SALEALYTSVK  NMTDQNYFLCR  DHHYTASWDEVK  NYGWTYEGPAWR  YEGIGWYGADTSKPK  GLRDHHYTASWDEVK  GDFTEESWNDFQTALSNAK  WGNYEGPAWEAPTSGGHLVYR  KFNVTVNHVNADTNAVLSSESK  IKWGNYEGPAWEAPTSGGHLVYR  VEPTNSVTLNNVTESQNYIFNYTR | Ext | Ext |
| Chaperone DnaK protein | EEI60127 | 3 | IAGLEVER  IPAVVEAVR  IIANPEGNR  GFAEDYLGEK  FQLTDIPAAPR  GFAEDYLGEKVEK  AKFDELTADLVER  AVITVPAYFNDAQR  DAGLSQSEIDEVILVGGSTR  KEEVDLRNDVDALLFSVDK  IVNEPTAAALAYGLDKTDRDEK  QALKDAGLSQSEIDEVILVGGSTR | Cyt | Cyt |
| Initiation factor 2:Small GTP-binding protein domain | EAN09509 | 3 | ITGQMIVR  AIGTVIEAR  TTLLDTLR  FVVFEDEK  VQADTEEVDIR  VTLDNLFESLK  QQQSTKPAVPPR  FVVFEDEKTAR  VGTIAGAYVTEGYIR  DGIVIYEGQLASLKR  EAGPATPVEITGLNDVPQAGDR  DKEAGPATPVEITGLNDVPQAGDR  GPVSTLLVQEGTLHVGDPIVVGNTYGR | Cyt | Cyt |
| Dak phosphatase | EAN08621 | 3 | IPYGIVAIAAGK  GNSGVILSQLFR  AVMKPVEGTILTVAR  VHVHTENPGEVLNYGQK  IGEGPTVDSTFDYEEFR  SVTEISASQFQEMVQAGANR  LQHETILEHDEEVNEFAK  NIFMAADQAAEVADIPVAVVPSR | Cyt | Ext |
| DNA gyrase, B subunit | EAN09583 | 3 | EFQAILPIR  FLLENPSVGR  IRELAFLNR  TLLLTLFYR  HPDPQFEGQTK  EGNVYYQEYR  NREFQAILPIR  RAFIEENAHYVK  QVVASLPASPKPSVQR  TGRPAVETVFTVLHAGGK  EYDASQIQVLEGLEAVR  AKEYDASQIQVLEGLEAVR  NAEEELKQVVASLPASPKPSVQR | Cyt | Cyt-Mem |
| Negative regulator of genetic competence ClpC/mecB | EFF32509 | 3 | GVFLPYSPR  AFRPEFLNR  LSEALLGGEIR  QDFENAAQLR  LLELEGLLHER  QTPQGTPTLDSLAR  AQAVLEIAQEEAKR  ALSEVMFGSEDALIR  IILGEVPEDMQEKR  IQVDEPTPEEAEVILQGLR  LIGSPPGYVGYDEGGQLTEKIR | Cyt | Cyt-Mem |
| Pyridoxal-dependent decarboxylase | EAN10847 | 3 | THSVPWHNAGR  IYADQLLASEAK  DLNLNALFIGDK  FYNFLNDLSFK  LNHDVYDYSSYVK  ALLNELVDEHLGWR  GADIPALLGAYILEGSK  AADIIGIGLDQVIPVPVDHNYR  YNVFTENKDYILEEVHSAYK | Cyt | Cyt |
| Conserved hypothetical protein | EAN08754 | 3 | TNQMLQFR  ADLVFYLPK  RVYDHAAIK  VRADLVFYLPK  MGAEPFEAFSER  VALPDGIGLEGHAK  EELGELTEEELAQR  FEHEFYYEAPAASILDIK  SQIEVNEDHISFQIPNKR | Cyt | Mem |
| Formate acetyltransferase | EAN08939 | 3 | DMQFFGAR  LNDIQFER  IITGLPDAYGR  LREEITEQYR  ISAFLDIYIQR  DFIQNNYTEYR  THNQGVFDAYTAEMR  GAYHLNVNVFSNDLLR  IAIESSSIQFENDNQLR | Cyt | Cyt |
| Glutamine-fructose-6-phosphate transaminase | EFR69396 | 3 | NAFYIGR  GFDVDKPR  GSYAFALIDR  VIIQNFAGDIVER  DYLEELAEVYLSDTR  NVTPGLIDGLEKLEYR  LGYPSLTITNVAGSTLSR  WATHGQPSEENAHPHTSQSGR  HGTIALIEEGTPVIGIISEEVTGAHTR | Cyt | Cyt |
| Methionyl-tRNA synthetase, class Ia:Methionyl-tRNA synthetase, beta subunit, C-terminal | EAN08866 | 3 | YGLDALR  GNVVYPEMLVER  SKGNVVYPEMLVER  VNYDLANDLGNLLNR  AVPFGSDGVFTPEDFVSR  APSGHEVELVKEESYFFR  LLEYYEEHPEFIQPESR | Cyt | Cyt |
| Prolyl-tRNA synthetase, bacterial | EAN09503 | 3 | YETYGPNLYR  DFTVLSYEDLR  AGYEVLVDDRNER  QVSAGIYSYLPLANR  SIDEVADFFSVEPQK  SVLFIADEQPVLVLVR  EVPNDAEVLSHQILLR  FVQEGDPSPDNNGVLAFTR | Cyt | Cyt |
| DNA gyrase subunit A | EEV44303 | 3 | MAQPFSYR  GYEIPEYGR  FNVSTVSQTGR  QILENYIEHQR  IALDHIDEIIAIIR  LIEDLTDILARPER  VTEIIKNELADLNQR  NTVDFQSNYDDTEKEPVVLPAR | Cyt | Cyt |
| Peptidase M41, FtsH | EAN10268 | 3 | QILVGRPDVK  SMVTEYGMSDR  IPAGVLLEGPPGTGK  SDVLDPALLRPGR  EDMFEQIVGLLGGR  NAPAIIFIDEIDAVGR  KNAPAIIFIDEIDAVGR  AYSEQVAFEIDQEVRK  VVAQQTPGFAGADLENVLNEAALVAAR  TAEEIIFNVQSTGASNDFEQATALAR | Mem | Mem |
| ClpE | EFF23737 | 3 | AHVIGQDNAVDR  LDVVSLVQGTGIR  YTDEAIEAAATLSNR  GELQMVGATTLNEYR  QLAYELFGSQDSMIR  AQNDPFGFGSLDDLFR  QNRPIGSFLFVGPTGVGK  SVLNQLNNYFTPEFLNR | Cyt | Cyt-Mem |
| MutS2 family protein | EFF27396 | 3 | LLIGVPGR  QYQIAER  YVIPVKQEYR  EAYSYFFEER  ILSELSAELVPHR  SESSSHVPNQLDLR  FIDDLYDSEIEFAR  TLKPGDEVLVTTYGQR | Cyt | Cyt |
| Glycyl-tRNA synthetase | EEV45769 | 3 | GQGLTTDAITFR  FLGEDVTFAHAK  LNEQFVIASPQKR  LEDAEFFYNEDKK  NDQGMLLPHFISAR  IIGEQVGLSEEELMDLQR  FLDENQLTYDTIETFSTPR | Cyt | Cyt |
| Protein of unknown function DUF814:Fibronectin-binding A, N-terminal | EAN09258 | 3 | LLLSAHPSYAR  NGELLTTFLTQVPR  SFDGVFTHAMVNELR  IHQPYENEIVLVIR  QFQGIGFDIAQELTKR  EVVLPNYYEEDRPIK | Cyt | Cyt-Ext |
| Extracellular solute-binding protein, family 5 | EAN09846 | 3 | YDDTFR  NEVLPVNDR  EIAGYTNLGR  EQTSYTYLGFK  EIGLDVQYTTGR  QAMGYALDNNAVGER  AFDEEYQKEAYQK | Ext | Mem |
| Polyribonucleotide nucleotidyltransferase | EFF36260 | 3 | QITEDLEKDVVR  DGLVHISQLANDR  KLDEIRPLSSEVSLLPR  ALAQVIPSEEEFPYTIR  FIHHYNFPQFSVGSTGR  TTWGGRPLQIEVGQLAK | Cyt | Cyt-Mem |
| Septation ring formation regulator | EEW64688 | 3 | QNEQMIPQIR  QHEIPYSEVR  TEVETVEFENR  EFTQFVTLNTSGDPVEAR  EYHYQEALDEIGTALER  VREDEHEYGPALFELQK | Cyt | Mem |
| Asparagine synthase, glutamine-hydrolyzing | EAN09487 | 3 | EFFDQEK  EYFIGHAR  HLPEEWSNR  ALFEADFAKEFFDQEK  GPNSSGEYIDKHVALGFR  LSIIDLEGGTQPIYNEDR | Cyt | Cyt |
| NLP/P60:Mannosyl-glycoprotein endo-beta-N-acetylglucosamidase | EAN10811 | 3 | VTSISDFTPDFAVR  SPIDAVLPTNTTNVQQAIVR  GSSVTFNTLEQDSSGQSYQIR | Ext | Ext |
| Threonyl-tRNA synthetase, class IIa | EAN10081 | 3 | LLSVAGAYWR  IVKENLPIER  QEALEIFASDPYK  IPYQLVVGDKELEDATVNVR  TALGTEETLSTIQLDFLLPER | Cyt | Cyt |
| fibronectin-binding protein A | EEV47622 | 3 | LLLSAHPSYAR  DGELLTTFLTQVPR  SFDGVFTHAMVNELR  IHQPYENEIVLVIR  EVVLPNYYEEDRPIK | Cyt | Cyt-Ext |
| arginyl-tRNA synthetase | EFF21615 | 3 | IYDLLEVR  LNTFDFNLEEVVR  QVGVGAVIFHDLKNDR  FEGETGPYVQYTHAR | Cyt | Cyt |
| RelA/SpoT protein | EAN09121 | 3 | IKWELEDTALR  EYDADLEIYGYNR  KQFNEIYDLLAIR  IAQETLEIYAPLAHR  IATEELGIFAEIYGRPK | Cyt | Cyt |
| DNA mismatch repair protein | EEV48697 | 3 | LALSHPSIAFR  ITVSNLFFNTPAR  ALSQENLIPSAADNLR  LANQIAAGEVVERPASVVK | Cyt | Mem |
| SagA | AAF86217 | 3 | YVYMQVTGR  QKAEAEAEQAR  DIGGWTVPQESAGTK  EAQVSNTSSNYIDAVLNADSLADAIGR | Ext | Ext |
| Phosphoenolpyruvate--protein phosphotransferase | EEI61237 | 3 | AFVTDIGGR  STEELQQIR  ATVEDTAAEEAR  ISLSEQGDEMFR  AVLEGMEGKPVVVR  VSYLYQPYNPSILR | Cyt | Cyt |
| Aspartyl-tRNA synthetase | EEV48334 | 3 | GIEVTLPFPR  DLGGVIFIDLR  QLLMNAGFDR  SEIVAAALGAIR  FAMLLAGEDNIR | Cyt | Cyt |
| SecA protein | EAN08595 | 3 | VLIVDQFTGR  ELGGLAVIGTER  FHAVVQDIKDR  TMATITFQNYFR  GETLDQLLPEAFAVVR | Cyt | Cyt |
| Acetyl-CoA acetyltransferase/HMG-CoA reductase | AAG02444 | 3 | FYQLTPSER  AVSASIHAYAAR  ILTTLAYGLLR  VNIYGGAIALGHPIGASGAR | Cyt-Mem | Cyt |
| ATP-dependent DNA helicase PcrA | EFF20026 | 3 | LAYVGITR  DVDFIGYNR  NYGDFAILYR  AEEALYLTNAFSR  NILSAANQVIENNSNR | Cyt | Cyt |
| CTP synthase | EAN10444 | 2 | TAEAYDGADVVQER  LKDADGILVPGGFGDR  QLFEEQGLVFSGVSPDNR | Cyt | Cyt |
| Small GTP-binding protein domain:GTP-binding protein LepA | EAN09614 | 2 | VDALSFIVHR  ALIFDSIYDSYR  VGDTVTLADNPAAEALPGYR | Cyt | Cyt |
| AAA ATPase, central region:Clp, N terminal | EAN09610 | 3 | GNFKPEFLNR  GELHLIGATTLDEYR | Cyt | Cyt-Mem |
| RNA binding protein S1 | EEV57375 | 2 | AYEQAIGFLR  ADEQAIAIFGENLR | Cyt | Cyt |
| Phosphoglucomutase/phosphomannomutase | EEV42904 | 2 | SPNPEEHSAFEYAIR | Cyt | Cyt |
| Lysyl-tRNA synthetase | EEI59161 | 3 | DVLLFPTMR  EGQIQIYVR  ITYDGQAVDLESDFKR  EFANAFTELNDPIDQR | Cyt | Cyt |
| FAD-dependent pyridine nucleotide-disulphide oxidoreductase | EAN09169 | 3 | WVIPWLQR  YGNVLWSVPLR  AHGFTLWSFEDSVR  VEPEAIQYDLQHLFAR | Mem | Cyt |
| HD domain-containing protein | EEV49044 | 2 | NLLSLAIQR  SESLENYIR | Cyt | Cyt |
| ABC transporter, ATP-binding protein | EFF31524 | 2 | ELQEFIAR  YPFVGFKPER  QYASKEEDDNTFLR | Cyt | Mem |
| Beta-lactamase-like:RNA-metabolising metallo-beta-lactamase | EAN10342 | 2 | NYDPEELIILETGR | Cyt | Cyt |
| Transketolase | EEV41175 | 2 | NIWFGVR  EVYGWEYPDFTVPEEVAAR | Cyt | Cyt |
| Peptidoglycan-binding LysM | EFF34034 | 3 | QYPSFAESFNDNAYVLR RYQLDASYLNGDYSAANQER  RIYVGEQLTIPTSNDSSATTENK | Ext | Ext |
| Metallo-beta-lactamase superfamily protein | EEV42569 | 2 | YFIPVHGEFR  IIFASFASNIFR | Mem | Ext |
| Penicillin-binding protein | EEV43240 | 3 | YLAKGYSR  QYEDVLQGTK  ASELPGVSTGTDWTR  EFGLGTTTGIDLPNESPGISR | Ext | Mem |
| DNA topoisomerase (ATP-hydrolyzing) | EEI60642 | 2 | FQAILPLR  KFQAILPLR  FSYEILAER | Cyt | Cyt-Mem |
| DNA directed DNA polymerase | EEV62180 | 2 | TSHAYLFTGPR  DYAPDPVYITR  ESTISYEPAALNVIAR | Cyt | Cyt-Mem |
| Asparaginyl-tRNA synthetase | EEV42969 | 2 | VFSFGPTFR  ATDYDFLLEQIR | Cyt | Cyt |
| Primosomal protein N | EEV57138 | 2 | GWLEFIESER  SSEHTFQLLTQVSGR | Cyt | Cyt |
| Haloacid dehalogenase-like hydrolase:Cation transporting ATPase, C-terminal:E1-E2 ATPase-associated region | EAN08923 | 2 | LNQPYQEIR  FQEQNEAFSTR | Mem | Mem |
| DNA topoisomerase I | EAN09573 | 2 | QHFTQPPAR  IVGYSISPILWR | Cyt | Cyt |
| Ribonucleoside-triphosphate reductase | EEI61284 | 2 | NEYELAEEYINYR | Cyt | Cyt |
| Excinuclease ABC, B subunit | EAN08843 | 2 | DLVSIQFER  VEFFGDEVER | Cyt | Cyt |

a Gene locus given by blast in the NCBI (http://www.ncbi.nlm.nih.gov/); b subcellular localization predicted by Cellov.2.5 (http://cello.life.nctu.edu.tw) and Gpos-mPLoc (<http://www.csbio.sjtu.edu.cn/bioinf/Gpos-multi>). Cyt, Cytoplasm, CW, cell wall. Ext, extracellular. Mem, membrane.
