# Supplementary material for: Identification of Peptidoglycan-Associated Proteins as Vaccine Candidates for Enterococcal Infections
Source: PLoS One. 2014 Nov 4;9(11):e111880. doi: 10.1371/journal.pone.0111880 (PMC4219796; doi:10.1371/journal.pone.0111880)
Supplement: Table S3 — Summary of all the proteins identified by biotinylation. (DOCX) [file pone.0111880.s003.docx]

**Table S3.** Summary of all the proteins identified by biotinylation.

| Protein name | Gene Locus^a^ | No of replicates | Peptides identify by MS analyses | Subcellular localization ^b^ | |
| --- | --- | --- | --- | --- | --- |
|  |  |  |  | CELLO v.2.5 | Gpos-mPLoc |
|  |  |  |  |  |  |
| Iron-containing alcohol dehydrogenase | EAN09662 | 3 | YDYFR  LVFDYLEK  TNAILLPHIIR  IGGEYGIPHGR  NPIVFAFHPSAQK  NSLLYLEKMENVER  YDYFRADTDYADIAK  TRNPIVFAFHPSAQK  AQGVTQETLDTTVDR  MLIAELEGVGPDYPLSR  AEFQAHQVYIVKPDELQK  MLIAELEGVGPDYPLSREK  GMWMFYEHPDTSFFGAK  NPIVFAFHPSAQKSSAEAAR  IEVFSDVEPNPSTNTVYAGTK  SMIALKTRNPIVFAFHPSAQK  GMWMFYEHPDTSFFGAKQK  TRNPIVFAFHPSAQKSSAEAAR  SAYSTGKPALGVGPGNVPAYIEK  ILVNTPSAEGGIGDIYNEMIPSLTLGCGSYGKNSVSR | Cyt-Mem | Cyt-Mem |
| SCP-like extracellular protein | EFF35540 | 3 | ELAWLNDYR  NYGWTYEGPAWR  IGVDNVFISANPIFK  SAEKNNKPIYRLFLPGVK  QQNGVAPMQFNDIVQQAADIR  WGNYEGPAWEAPTSGGHLVYR  KFNVTVNHVNADTNAVLSSESK  VEPTNSVTLNNVTESQNYIFNYTR  IKWGNYEGPAWEAPTSGGHLVYR  NSPAHNANLLYNNQSILGLGHNFVVDSAGR | Ext | Ext |
| Low affinity penicillin-binding protein 5 (PBP5) | EFF35784 |  | FATGYAPGSTFK  DSSWGSYQVTR  SGLEMAFDKDLR  WQKDSSWGSYQVTR  NVIGETALQTIVPDLR  TVEQFVQALNKGDYNK  IIDGATPELPAGATIQEVDGR  SALSEKEILDKYQNIYGAADVK | Ext | Ext |
| Ribosomal protein L2, bacterial and organelle form | EAN09405 | 2 | KAPVSPWGQPALGYKTR  NKDNVVATVQTIEYDPNR  ATIGSVGNEQHELINIGKAGR  GSVMNPNDHPHGGGEGKAPIGR | Cyt-Mem | Ext |
| Ornithine carbamoyltransferase | EAN09541 |  | MKESVFQGR  MFDGIEFRGFSQK  AIMAATLGNLFIPRV | Cyt | Cyt |
| Amino acid ABC transporter, permease protein, 3-TM region, His/Glu/Gln/Arg/opine | EAN08842 | 3 | IILPQAIR  LQGFNIR  SLGLSYNR  GEYEGIDVK  YFDAADQLYDGLK  GGINAVPVGQMEASR  KGQNPELLNMFNEGLK  SYDFSDSYFDSGIQLAVK  GQNPELLNMFNEGLKELKR  VGTESAEFLSDHESEYGYSIK  KDVYVIASDSAFAPFEFQNAK  KDVYVIASDSAFAPFEFQNAKGEYEGIDVK | Mem | Mem |
| Glyceraldehyde-3-phosphate dehydrogenase | EEI59791 |  | FNGTVEVHEGSFNVNGK  AIGLVIPELNGKLDGAAQR  IQDVDGIEVVAINDLTDAK | Cyt-Mem | Cyt-Mem |
| Basic membrane lipoprotein | EFF34523 | 3 | TIFGIGYK  VWVIGVDR  NFTLTSTLK  KVWVIGVDR  WGKDQGLSR  AVGTVVEDLAQK  FPGGEHTVYGLK  GEVIDRFDAGFK  SFNQSAWEGLEK  VLNQYAGDFSAPDK  EDGVGLTEGQLSDEAK  VLNQYAGDFSAPDKGR  EDGVGLTEGQLSDEAKK  SSSDTTTAALITDTGGVDDR  VWVIGVDRDQSDEGEYTLNGEK | Ext | Mem |
| Flavocytochrome c | EEV42250 | 3 | ATAIEFYDQK  SAYLIFDQGVR  IGGNSVAEIIIFGR  SAYLIFDQGVRDR  INTNTEVLDKENQPIR | Ext | Ext |
| PTS system mannose/fructose/sorbose family IID component | EAN10754 | 3 | LAVAWR  SNVSKLSSKDLR  STFIQGSWNYER | Mem | Mem |
| Peptidoglycan-binding protein LysM | EFF34034 | 3 | RYQLDASYLNGDYSAANQER  HQSTENNETQEPWEQPIYDTDDEVSSR  AGITTDQLFELNGLDPNNFMLYPGQELR | Ext | Ext |
| Enolase | EAN08760 | 3 | SIITDVYAR  AVDNVNNIIAEAIIGYDVR  GMVPSGASTGEYEAVELR  AAADYLEVPLYHYLGGFNTK  SGETEDSTISDIAVATNAGQIK  LTDVLGDKVQLVGDDLFVTNTQK | Cyt | Cyt |
| Penicillin-binding protein, 1A family | EFR77957 | 2 | VGGELYYKDQYTAR  HIAEDVTLGNNLAVNTSR  NVYTDGLEIYTNLDLDAQK | Ext | Ext |
| FMN-binding protein | EAN09170 | 3 | YIPELNDQFLK  TYIPTLNDEFVK  SGTNPEKYIPELNDQFLK | Ext | Ext |
| Ketose-bisphosphate aldolase, class-II:Fructose-1,6-bisphosphate aldolase, class II | EAN10443 | 2 | PVVSGAEFLKAAR  QAPVLIQTSMGAAK | Cyt | Cyt |
| D-alanyl-D-alanine carboxypeptidase | EFF35669 | 3 | ANIFVIGWR  RVAEFFGNL  AAEAAGFPLVMVSAYR  SVSSQQQVFEQNVQDVMSR | Mem | Mem |
| Ribosomal protein L5 | EEI61165 | 3 | LVSVSLPR  FNYSSVMQTPKVEK | Cyt | Cyt |
| PTS system protein | EEV44776 | 2 | IIVVSDAVSKDDLR  VDSRLLHGQVATAWTK | Cyt | Cyt |
| Phage minor tail protein | EEV47247 | 2 | NQLDQMSDSSK  NGTLTLPDMLNK | Ext | Ext |
| PpiC-type peptidyl-prolyl cis-trans isomerase | EFF34785 | 3 | DTYKEYIR  NNLAFEAGLK  LESSNQSLVQR  SFHPEVEAQIIK  GGTITVSDFYDEAK  SLGDTFESQLEAAGYTK  GGTITVSDFYDEAKLESSNQSLVQR | Ext-Mem | Ext-Mem |
| Aminodeoxychorismate lyase | EEI61390 | 3 | NIAQVFFNR  IAIPEGYDIDQIAER | Cyt | Cyt |
| Extracellular solute-binding protein | EEV50964 | 3 | FYAGLR  YDDTFR  NEVLPVNDR  EQTSYTYLGFK  EIGLDVQYTTGR  QAMGYALDNNAVGER  AFDEEYQKEAYQK | Ext | Ext |
| Protein kinase:PASTA | EAN10248 | 3 | FDFQNDQTAIR  ALDPNVPQALENVILR | Mem | Mem |
| Formate acetyltransferase | EAN08939 | 2 | IITGLPDAYGR  ISAFLDIYIQR | Cyt | Cyt |
| Ribosomal protein L15, bacterial form | EAN09421 | 2 | LGFEGGQTPLFR | Cyt | Cyt |
| DNA-entry nuclease | EEI59681 | 3 | LSDGQWLYNR  VTPHFEGDELVAR | Ext | Ext |
| Penicillin-binding protein 1b | EAN08787 | 3 | IYGFVGGR  IVNATNEGSNTFQTVR  NYSQNQNNHAFDTER | Ext-Mem | Ext |
| Translation elongation factor Tu | EEI61151 | 2 | LLDYAEAGDNIGALLR  GITISTAHVEYETDTR  KLLDYAEAGDNIGALLR | Cyt | Cyt |
| Protease IV homolog | AAD29142 | 2 | EQYNHQNFLTQLK  EQYNHQNFLTQLKK  IYDGNQAVENGLVDQIGYSEDALDSLKK | Mem | Mem |
| Peptidase M41, FtsH | EAN10268 | 2 | NAPAIIFIDEIDAVGR  TAEEIIFNVQSTGASNDFEQATALAR | Mem | Mem |
| Ribosomal protein S3 | EEV48626 | 2 | SEGYSEGTVPLHTLR  WYAEKEYAEFLHEDLR | Cyt | Cyt |
| Glutamine synthetase type I | EAN10445 | 2 | NIYVMDEEER  LVPGYEAPVYVAWSGR | Cyt | Cyt |
| Glycosyl transferase | EEV52587 | 3 | VGGELYYKDQYTAR  LYTQDGELFEDLGAEKR | Ext | Ext |
| DNA directed RNA polymerase | EEV49133 | 3 | SVGELLQNQFR  LGIQAFEPVLVQGR | Cyt | Cyt |
| Arginine deiminase | EAN09542 | 2 | ESMFTEYILK  AALGLDDLVLIPTGNGDEIVAPR | Cyt | Cyt |
| ApbE-like lipoprotein | EAN09171 | 2 | KPSQEEIDQALK  ILKDPYSDEQFLLGTYVR | Mem | Mem |
| NLPA lipoprotein | EAN09985 | 2 | DNSPYVNIIATR  DGATIITSNSESDWGR | Mem | Mem |
| Alkyl hydroperoxide reductase/ Thiol specific antioxidant/ Mal allergen | EAN09700 | 2 | AWHDTSDAIGKI  FDVLDEEQGLAQR | Cyt | Cyt-Ext |
| Gp32 | EFF22181 | 2 | NQFEENQPAQVVK  VGDAEYTLNSVELTDER | Mem | Ext |
| Extracellular solute-binding protein, family 5 | EAN09846 | 2 | FYAGLR  YDDTFR  TSYIEFNQR | Ext | Ext |
| Lysis protein | EEI60331 | 2 | METENPETLDR  IEGVDDQKEYEAR | Ext | Ext |
| Extracellular solute-binding protein, family 3 | EAN08986 | 2 | WFGEDVTPR  LGAQNGSSGYDVFTK | Cyt-Mem | Mem |
| Periplasmic solute binding protein | EAN10630 | 2 | ISGVSPDQEPTP  EVPVLFVETSVDSR  AKEVPVLFVETSVDSR | Cyt-Mem | Mem |

a Gene locus given by blast in the NCBI (http://www.ncbi.nlm.nih.gov/); b subcellular localization predicted by Cellov.2.5 (http://cello.life.nctu.edu.tw) and Gpos-mPLoc (<http://www.csbio.sjtu.edu.cn/bioinf/Gpos-multi>). Cyt, Cytoplasm, CW, cell wall. Ext, extracellular. Mem, membrane.
