# Supplementary material for: Identification of Peptidoglycan-Associated Proteins as Vaccine Candidates for Enterococcal Infections
Source: PLoS One. 2014 Nov 4;9(11):e111880. doi: 10.1371/journal.pone.0111880 (PMC4219796; doi:10.1371/journal.pone.0111880)
Supplement: Table S4 — Summary of all the proteins identified by at least two of the three extraction methods and predicted to have both cytoplasmic and extracytoplasmic location. (DOCX) [file pone.0111880.s004.docx]

**Table S4.** Summary of the proteins identified by at least two of the three extraction methods and predicted to have both cytoplasmic and extracytoplasmic location.

| Protein name | Gene Locus^a^ | | Subcellular localization ^b^ | | Extraction method | | |
| --- | --- | --- | --- | --- | --- | --- | --- |
|  |  |  | CELLO v.2.5 | Gpos-mPLoc | Biot^*^ | Tryp^§^ | HpH^$^ |
| Ribosomal protein L2, bacterial and organelle form | EAN09405 | | Cyt-Mem | Ext | + | + | + |
| Glyceraldehyde-3-phosphate dehydrogenase | EEI59791 | | Cyt-Mem | Cyt-Mem | + | + | + |
| Ribosomal protein S9 | EAN10220 | | Cyt | Mem-Cyt-Ext | - | + | + |
| 50S ribosomal protein L24 | EFF30351 | Mem-Cyt-Ext | | Cyt | - | + | + |
| Nucleoside 2-deoxyribosyltransferase | EAN09913 | | Cyt | Ext | - | + | + |
| Dak phosphatase | EAN08621 | | Cyt | Ext | - | + | + |
| Ribosomal protein L2 | EEV41714 | | Cyt-Ext | Ext | - | + | + |
| Ribosomal protein S11 | EAN09427 | | Cyt-Ext | Cyt | - | + | + |
| Ribosomal protein L20, bacterial and organelle form | EAN08876 | | Cyt-Ext | Mem | - | + | + |
| Malonyl CoA-acyl carrier protein transacylase | EAN10060 | | Cyt-Mem | Mem | - | + | + |
| Sporulation initiation inhibitor protein soj | EHM33068 | | Cyt-Mem | Cyt-Mem | - | + | + |
| 50S ribosomal protein L4 | AFK57682 | | Cyt-Mem | Cyt-Mem | - | + | + |
| Acetyl-CoA carboxylase carboxyl transferase, β-Subunit | EAN10066 | | Mem | Cyt | - | + | + |
| FAD-dependent pyridine nucleotide-disulphide Oxidoreductase | EAN09169 | | Mem | Cyt | - | + | + |
| Ribosomal protein L17 | EAN09429 | | Cyt | Mem | - | + | + |
| ABC transporter | EAN08750 | | Cyt | Mem | - | + | + |
| Putative ABC transporter | CAD21830 | | Cyt | Mem | - | + | + |
| Chromosomal replication initiator protein, DnaA | EAN09579 | | Cyt | Mem | - | + | + |
| Acetyl-CoA carboxylase, biotin carboxylase | EAN10065 | | Cyt | Mem | - | + | + |
| ClpX, ATPase regulatory subunit | EAN09535 | | Cyt | Mem | - | + | + |
| Septation ring formation regulator | EEW64688 | | Cyt | Mem | - | + | + |
| DNA mismatch repair protein | EEV48697 | | Cyt | Mem | - | + | + |
| ABC transporter, ATP-binding protein | EFF31524 | | Cyt | Mem | - | + | + |
| DNA gyrase, B subunit | EAN09583 | | Cyt | Cyt-Mem | - | + | + |
| ClpE | EFF23737 | | Cyt | Cyt-Mem | - | + | + |
| Polyribonucleotide nucleotidyltransferase | EFF36260 | | Cyt | Cyt-Mem | - | + | + |
| AAA ATPase, central region:Clp, N terminal | EAN09610 | | Cyt | Cyt-Mem | - | + | + |
| HAD-superfamily hydrolase | EEV43241 | | Cyt | Cyt-Mem | - | + | + |
| GntR family transcriptional regulator | EEI60711 | | Cyt | Cyt-Mem | - | + | + |
| DNA-directed RNA polymerase | EAN09428 | | Cyt | Cyt-Mem | - | + | + |
| Cell division transporter substrate-binding protein FtsY | EAN09850 | | Cyt | Cyt-Mem | - | + | + |
| UDP-N-acetylmuramate--alanine ligase | EAN08897 | | Cyt | Cyt-Mem | - | + | + |
| Cell division protein FtsA | EAN10731 | | Cyt | Cyt-Mem | - | + | + |
| Sodium-transporting two-sector ATPase | EAN10388 | | Cyt | Cyt-Mem | - | + | + |
| Glutamyl-tRNA(Gln) amidotransferase A subunit | EAN09524 | | Cyt-Mem | Cyt | - | + | + |
| PTS system fructose subfamily IIA component:PTS System sorbose subfamily IIB component | EAN08784 | | Cyt-Mem | Cyt | - | + | + |
| NADH oxidase | EEV50506 | | Cyt-Mem | Cyt | - | + | + |
| Beta-ketoacyl synthase | EAN10062 | | Cyt-Mem | Cyt | - | + | + |

a Gene locus given by blast in the NCBI (http://www.ncbi.nlm.nih.gov/); b subcellular localization predicted by Cellov.2.5 (http://cello.life.nctu.edu.tw) and Gpos-mPLoc (<http://www.csbio.sjtu.edu.cn/bioinf/Gpos-multi>). CW, cell wall. Ext, Ext. Mem, membrane. *Biot; Biotinylation. § Tryp; Trypsin shaving. $HpH; Elution at high pH.
